# Supplementary material for: Crowd control: Reducing individual estimation bias by sharing biased social information
Source: PLoS Comput Biol. 2021 Nov 29;17(11):e1009590. doi: 10.1371/journal.pcbi.1009590 (PMC8659305; doi:10.1371/journal.pcbi.1009590)
Supplement: S1 Appendix — Further details about the experimental design are provided (including the questions asked), as well as figures and tables supporting the statistical analysis and the main discussion. Fig A: Experimental procedure for an example question. Fig B: Correlation between median estimate and correct answer for general knowledge VS numerosity questions and for very large VS moderately large quantities. Fig C: Significance analysis of the differences between slopes in all panels of Fig 1 as well as of these slopes being lower than 1. Fig D: Narrowing of the distributions of estimates after social information sharing in Fig 2 and analysis of its significance. Fig E: Probability density function (PDF) of personal estimates Xp for all conditions combined. Fig F: Probability density function (PDF) of the fraction of instances with S = 0 for each participant and each question. Fig G: Significance analysis of the differences in Pg, mg and σg between treatments in Fig 4. Fig H: 〈S〉 against τ and 〈σ〉 for the model without similarity effect. Fig I: 〈S〉 against τ, when D < 0 and D > 0, for the model without similarity effect. Fig J: Collective and individual accuracy against τ for the model without similarity effect. Fig K: 〈S〉 against τ and 〈σ〉 for the model without asymmetry effect. Fig L: 〈S〉 against τ, when D < 0 and D > 0, for the model without asymmetry effect. Fig M: Collective and individual accuracy against τ for the model without asymmetry effect. Fig N: Significance analysis of the improvements in collective and individual accuracy in Fig 12. Fig O: Significance analysis of the difference in improvement in collective accuracy between treatments in Fig 12. Fig P: Significance analysis of the difference in improvement in individual accuracy between treatments in Fig 12. Fig Q: Significance analysis of the improvement in individual accuracy in Fig 13. Fig R: Collective accuracy against τ when D < 0 and when D > 0. Fig S: Significance analysis of the improvement in individual ac [file pcbi.1009590.s001.pdf]

Appendix for the article entitled: Crowd control: reducing individual estimation bias by sharing biased social information

Bertrand Jayles[1,2,*], Clément Sire[3], Ralf H. J. M. Kurvers[1]

[1]Center for Adaptive Rationality, Max Planck Institute for Human Development, Berlin, Germany

[2]Institute of Catastrophe Risk Management, Nanyang Technological University, Singapore, Republic of Singapore

[3]Laboratoire de Physique Théorique, Centre National de la Recherche Scientifique (CNRS), Université de Toulouse – Paul Sabatier (UPS), Toulouse, France

1 Experimental design

Participants were 216 students, distributed over 18 groups of 12 individuals, from the Bielefeld University, taking an Introductory Biology course (16-18 April 2018). Prior to participation, all participants signed an informed consent form and the experiment was approved by the Institutional Review Board of the Max Planck Institute for Human Development (A 2018/11).

Each of the 12 subjects—in each of the 18 groups—was confronted with 36 estimation questions (see the list in section 2) on a tactile tablet (Lenovo TAB 2 A10-30). Each question was asked twice: first, subjects provided their personal estimate E_{p}. Next, they received as social information the estimate(s) of one or more group members (*i.e.* other subjects in

*Corresponding author – bertrand.jayles@ntu.edu.sg

the same room at the same time), and were asked to provide a second estimate E_s. As a reminder, their personal estimate was also shown during the second answering of a question. Supplementary Fig A below illustrates how social information was displayed on the tablets: on the right side of the screen was a blue panel showing all pieces of social information, sorted in increasing order. All tablets were controlled by a central server, and participants could only proceed to the next question once all individuals provided their estimate. A 30 seconds count down timer was shown on the screen to motivate subjects to answer within this time window, although they were allowed to take more time.

When providing social information, we varied (i) the number of estimates selected (1, 3, 5, 7, 9, or 11), and (ii) the selection procedure (Random, Median, and Shifted-Median). In the Random treatment, subjects received random estimates from their 11 group members. In the Median treatment, we presented the estimates of which logarithm was closest to the median of the logarithms of the 12 personal estimates. Indeed, the logarithmic scale is consistent with the logarithmic perception of numbers [43]. In the Shifted-Median treatment, subjects were provided the estimates of which logarithm was closest to a shifted (overestimated) value of the median of the logarithms of the 12 personal estimates (see Main Text, Material and Methods). The participants were not aware of these different treatments.

Importantly, in all treatments, subject did not receive their own estimate as social information. In total, there were 6 different numbers of estimates selected $\times$ 3 treatments = 18 unique conditions. In every session, the 36 questions were randomly assigned to six blocks of six questions. Across groups, the order of the blocks, and the questions within a block, were randomized. A block always contained each number of estimates to be shown (1, 3, 5, 7, 9 and 11) once and was assigned one of the three treatments (Random, Median or Shifted-Median). Each group experienced two blocks of each treatment, and thus each of the 18 unique conditions twice. The randomization was constrained in such a way that at the end of the whole experiment, *all* 36 questions were asked once in *all* 18 different conditions, resulting in 36 estimates (1 per question) $\times$ 12 subjects = 432 estimates ($\times$2: before and

after receiving social information) per condition.

Students received course credits for participation. Additionally, we incentivized them based on their performance P, defined as:

$$P_i = \frac{1}{2}\left(\text{Median}_q \left|\log\left(\frac{E_{\mathrm{p}_{i,q}}}{T_q}\right)\right| + \text{Median}_q \left|\log\left(\frac{E_{\mathrm{s}_{i,q}}}{T_q}\right)\right|\right),$$

where i and q index individuals and questions, E_p and E_s are estimates before ("personal") and after ("second") receiving social information, and T is the correct answer to the question at hand. This performance criterion measures, for each individual, the median distance (in terms of orders of magnitude) of their estimates to the corresponding correct answers to all questions, averaged over the two estimates (before and after receiving social information). The payments were defined according to the distribution of performances measured in [20]:

- $P_i < 0.4$: 5€ ($\sim$ 20% of subjects)
- $0.4 \leq P_i < 0.5$: 4€ ($\sim$ 30% of subjects)
- $P_i \geq 0.5$: 3€ ($\sim$ 50% of subjects)

2 List of questions

Below is the list of questions used in the experiment and the corresponding true values T. In the original experiment, the questions were asked in German. Questions were a mix of general knowledge and numerosity, i.e., estimating the number of objects (e.g. marbles, matches, animals) in an image. Images were shown for 6 seconds. 18 questions were taken from a previous study [20], and 18 were new (shown in italic). Questions 21 and 32 were the same in [20], but were asked in different units, such that the true answer and corresponding estimates were substantially different. Therefore, we considered these as new.

1. What is the population of Tokyo and its agglomeration? $T = 38,000,000$

2. What is the population of Shanghai and its agglomeration? $T = 25,000,000$

3. What is the population of Seoul and its agglomeration? $T = 26,000,000$

4. What is the population of New-York City and its agglomeration? $T = 21,000,000$

5. What is the population of Madrid and its agglomeration? $T = 6,500,000$

6. What is the population of Melbourne and its agglomeration? $T = 4,500,000$

7. *How many ebooks were sold in Germany in 2016? T = 28,100,000*

8. How many books does the American library of Congress hold? $T = 16,000,000$

9. How many people died from cancer in the world in 2015? $T = 8,800,000$

10. *How many smartphones were sold in Germany in 2017? T = 24,100,000*

11. *What was the total distance of the 2016 Tour de France (in kilometers)? T = 3,529*

12. *How many insured cars were stolen in Germany in 2016? T = 18,227*

13. Marbles 1: How many marbles do you think are in the jar in the following image? $T = 100$

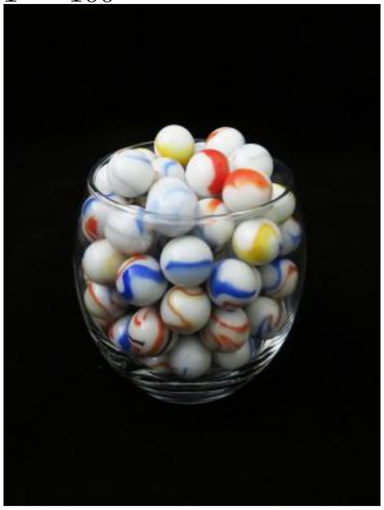

14. Marbles 2: How many marbles do you think are in the jar in the following image? $T = 450$

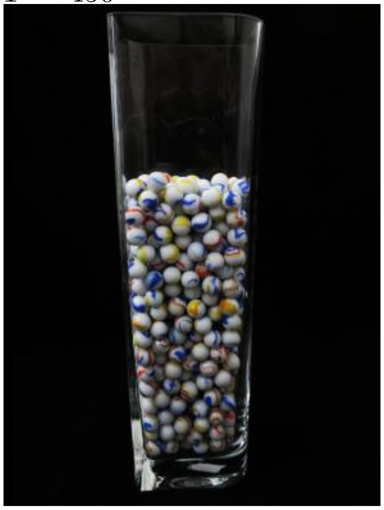

15. Matches 1: How many matches do you think are present in the following image? $T = 240$

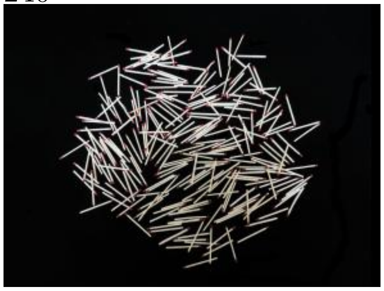

16. Matches 2: How many matches do you think are present in the following image? $T = 480$

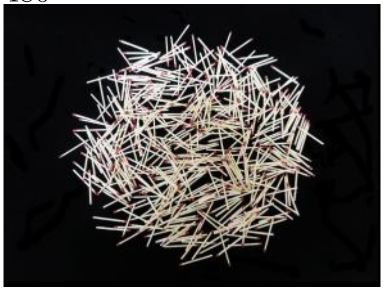

17. How many people identify as indigenous in Mexico? $T = 6,000,000$

18. How many cars were registered in Germany in 2016? $T = 45,071,000$

19. What is the diameter of the Sun (in kilometers)? $T = 1,391,400$

20. What is the distance between Earth and the Moon (in kilometers)? $T = 384,400$

21. How many stars does the Milky way hold? $T = 235,000,000,000$

22. How many kilometers is one light-year (in billion kilometers)? $T = 9,460$

23. How much is the per-day income of Mark Zuckerberg (in dollars)? $T = 4,400,000$

24. How many cells are there in the human body (in billion cells)? $T = 100,000$

25. How many bees do you think are in this picture? $T = 976$

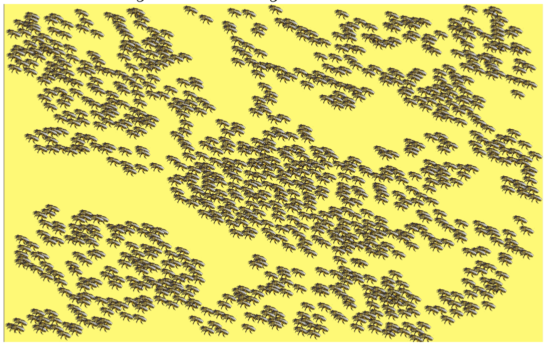

26. What is the average annual salary of a player in the Bundesliga (in euros)? $T = 1,456,565$

27. How many gnus do you think are in this picture? $T = 483$

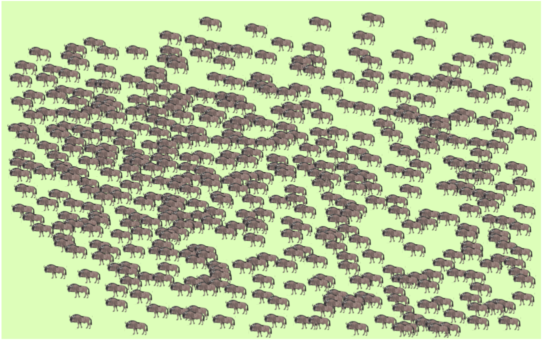

28. How many bikes do you think there are in Germany? $T = 62,000,000$

29. What is the distance from planet Mercury to the Sun (in kilometers)? $T = 58,000,000$

30. What is the total length of the metal threads used in the braided cables of the Golden Gate Bridge (in kilometers)? $T = 129,000$

31. What is the mass of the pyramid of Cheops (in tons)? $T = 5,000,000$

32. *How much did the building of the Burj Khalifa tower in Dubai cost (in dollars)? $T = 1,500,000,000$*

33. *What is the average salary for players at Bayern Munich (in euros)? $T = 5,460,000$*

34. *What is the distance from Berlin to New-York (in kilometers)? $T = 6,188$*

35. *How many tourists were recorded in France in 2016? $T = 82,600,000$*

36. *How many UFO sightings have been reported to the National UFO Reporting Center in its history? $T = 90,000$*

3 Supplementary figures

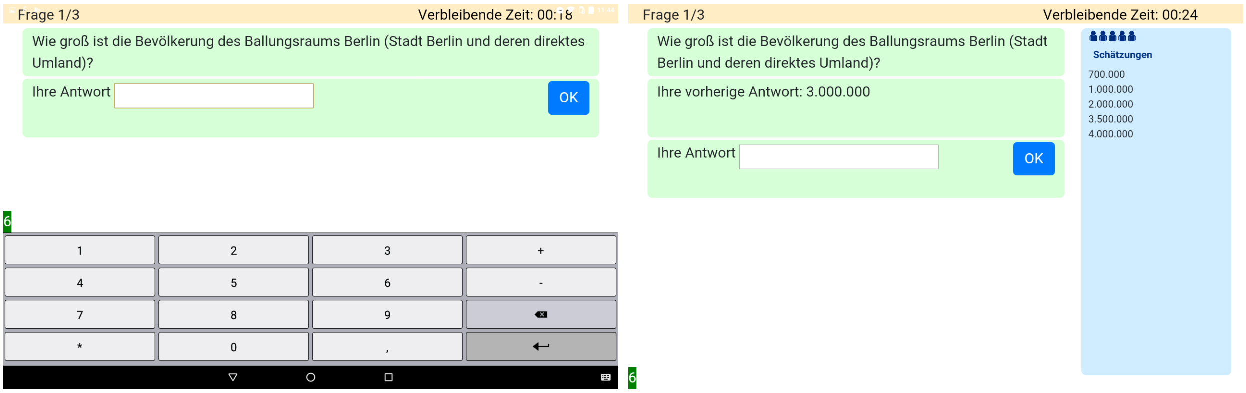

Fig A: **Experimental procedure for an example question.** The left panel shows the first screen in which subjects had to provide their personal estimate. The question was asked on the first line, and the answer could be typed on the second line, using a keyboard that appeared when clicking on "Ihre Antwort" ("Your answer" in German). Subjects submitted their estimates by pushing the "OK" button. A timer was displayed in the top right corner of the screen to remind subjects to answer within 30 seconds. The right panel shows the second screen in which subjects could revise their estimate after observing answers from other group members (in this example 5 answers). As a reminder, the original question, as well as the subject's personal estimate were shown. Subjects provided their second estimate in the same way as the first one and the countdown timer was again set on 30 seconds.

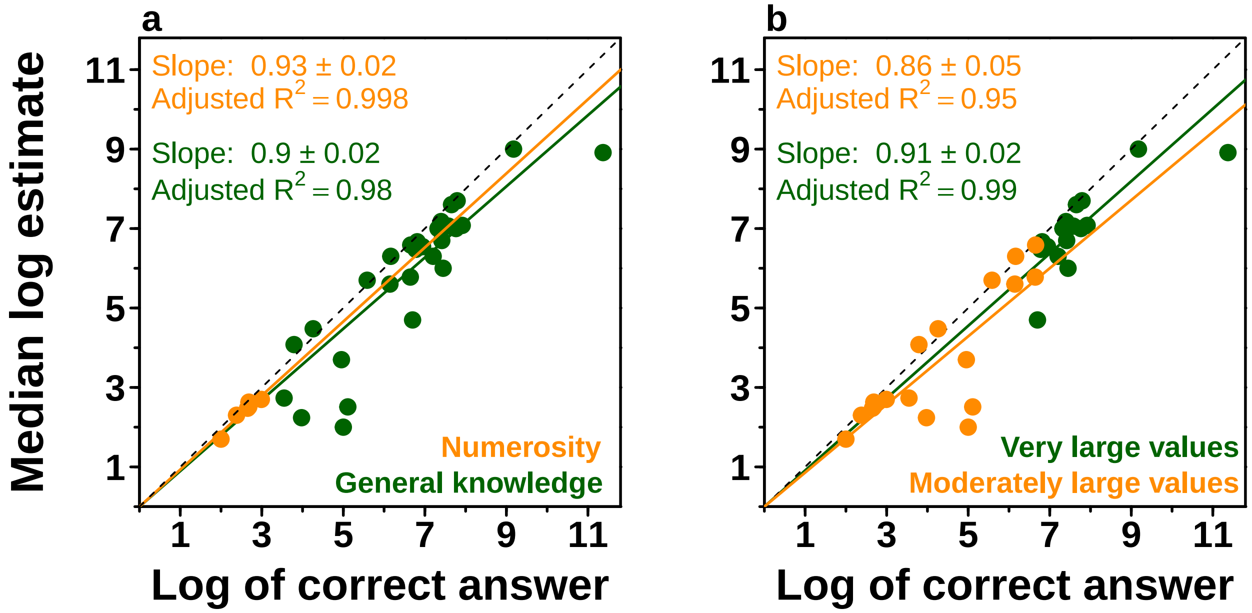

Fig B: Correlation between median estimate and correct answer for general knowledge VS numerosity questions and for very large VS moderately large quantities. Median of the log-estimates against the logarithm of the correct answer for the 36 questions asked in our experiment (one dot per question). (a) Green colors represent general knowledge questions, and orange numerosity questions, i.e., estimating the number of objects in an image. The slopes of the linear regression lines are 0.9 and 0.93 respectively, suggesting a similar relationship for both classes; (b) Green colors represent the 18 questions with the largest true values, and orange the 18 questions with the smallest true values. The slopes of the linear regression lines are 0.91 and 0.86 respectively, suggesting that the degree of underestimation is robust across different magnitudes.

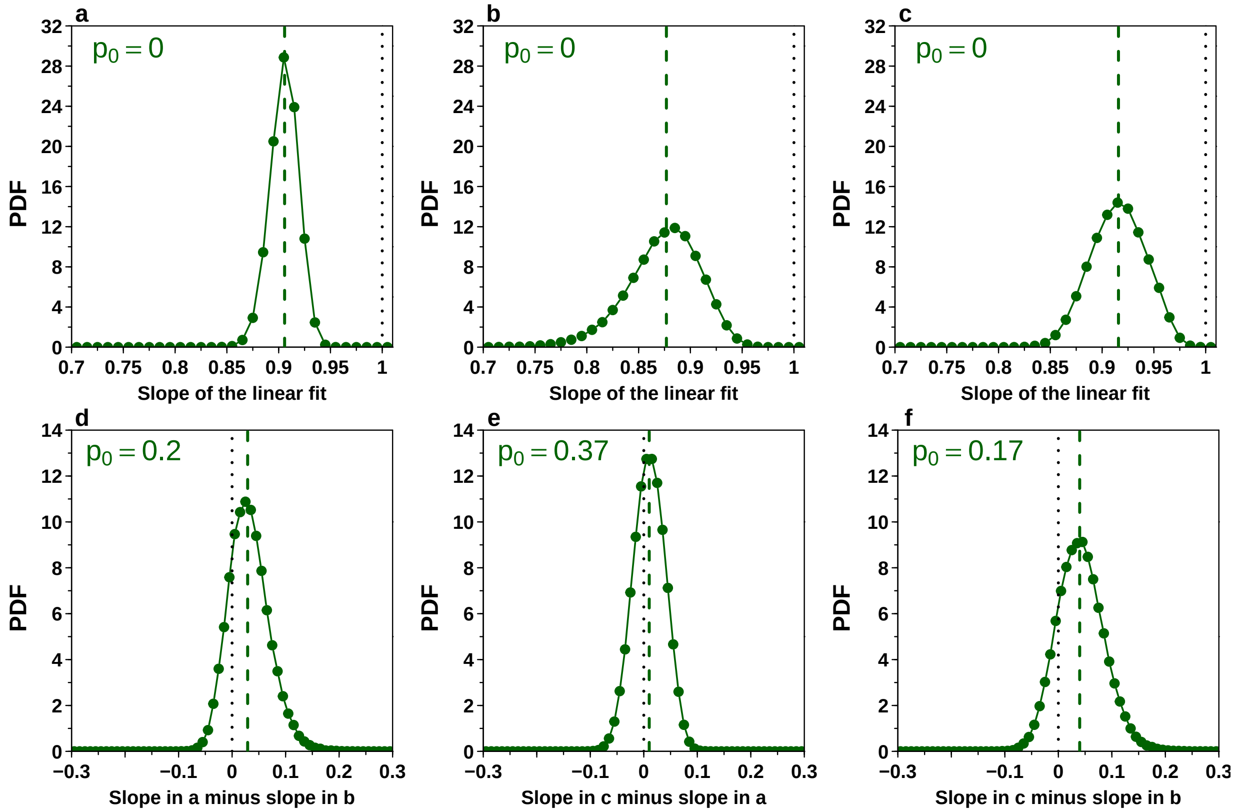

Fig C: **Significance analysis of the differences between slopes in all panels of Fig 1 as well as of these slopes being lower than 1.** Top row: Probability density function (PDF) of the bootstrapped slopes in (a) Fig 1a, (b) Fig 1b and (c) Fig 1c. The slopes are significantly lower than 1 in all cases (the reference line at 1 is shown as a dotted line). Bottom row: Probability density function (PDF) of the bootstrapped differences between the slopes in (d) Fig 1a and Fig 1b, (e) Fig 1c and Fig 1a and (f) Fig 1c and Fig 1b. The slopes are not significantly different from each other (the reference at 0 is shown as a dotted line). Dashed lines show the median slope (top row) or difference in slope (bottom row).

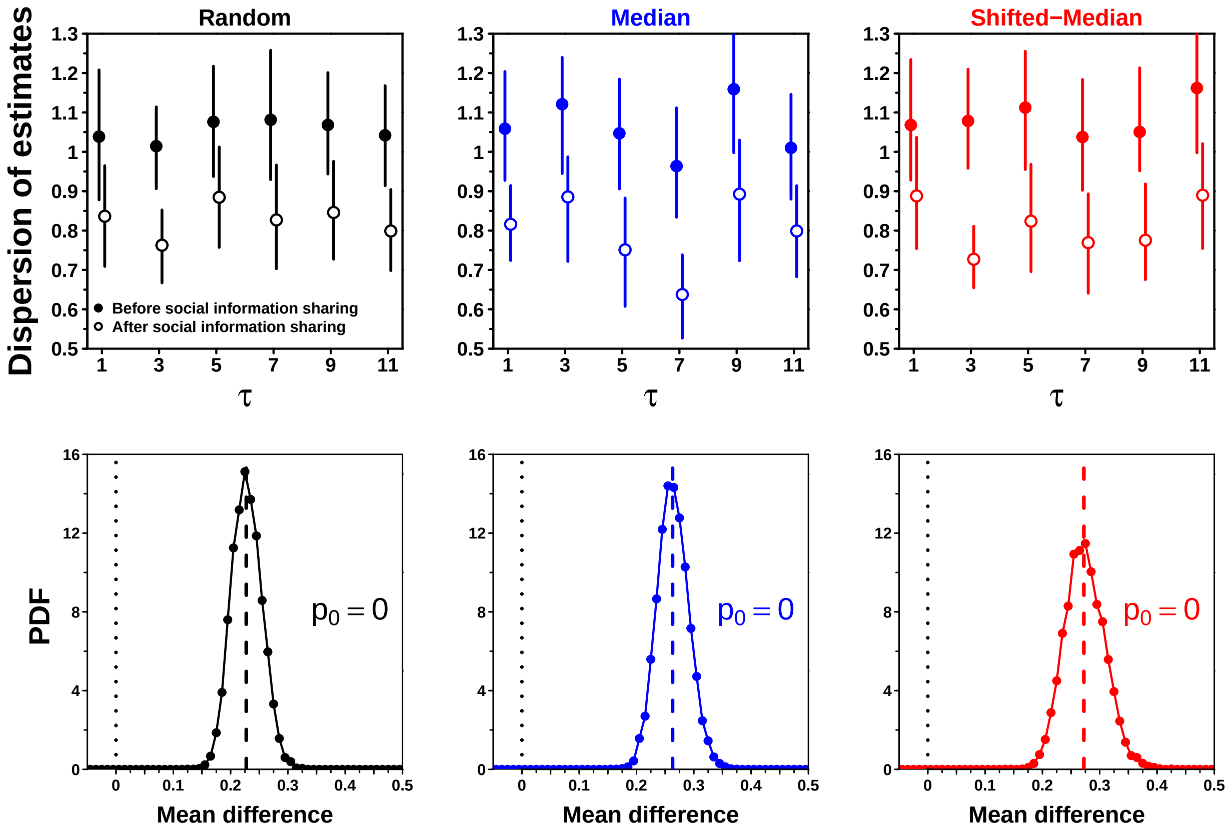

Fig D: **Narrowing of the distributions of estimates after social information sharing in Fig 2 and analysis of its significance.** Top row: Dispersion of estimates, defined as the average absolute deviation of estimates from their median, before (filled dots) and after (empty dots) social information sharing for all values of τ in the Random (black), Median (blue), and Shifted-Median (red) treatments. The dispersion decreases – i.e., the distribution of estimates narrows – after social information sharing in all conditions. Bottom row: Probability density function (PDF) of the bootstrapped differences between the average values (over all values of τ) of the dispersion of estimates before social information sharing and that after social information sharing, in the Random (black), Median (blue) and Shifted-Median (red) treatments. The average decrease (i.e., the narrowing of the distribution) over all values of τ is highly significant in all treatments ($p_0 = 0$). Dashed lines show the median value of the bootstrapped mean improvement, and the reference at 0 is shown as a dotted line.

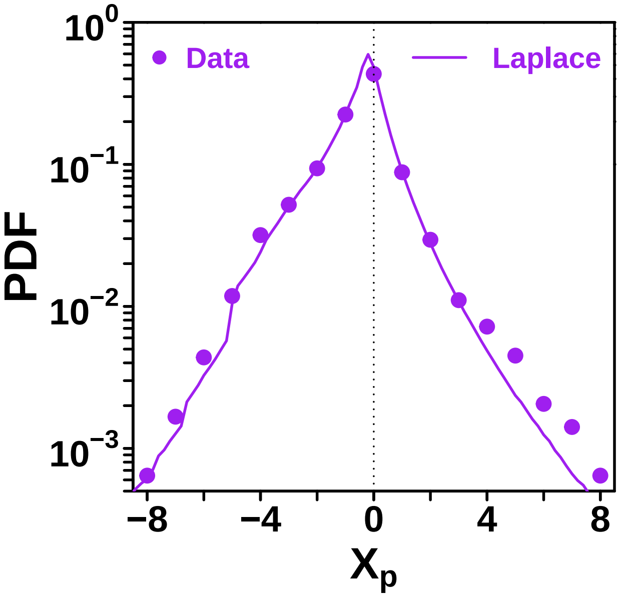

Fig E: **Probability density function (PDF) of personal estimates X_{p} for all conditions combined.** Dots are the data and the line model simulations, based on Laplace distributions for each question.

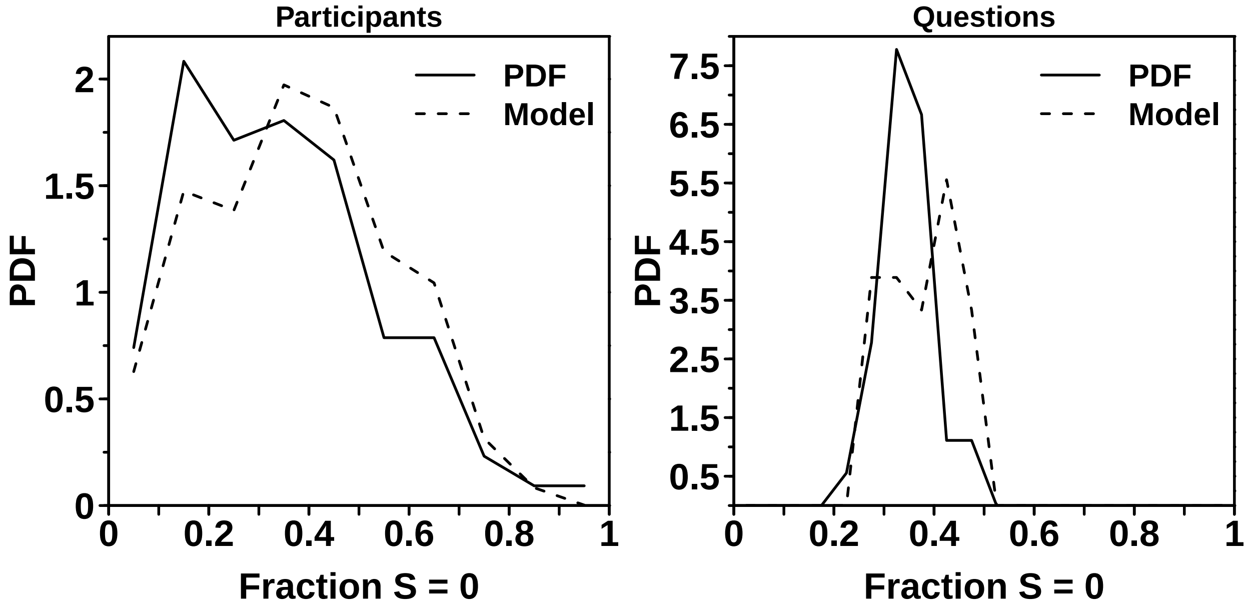

Fig F: Probability density function (PDF) of the fraction of instances with $S = 0$ for each participant and each question. We also present the model predictions (dashed lines).

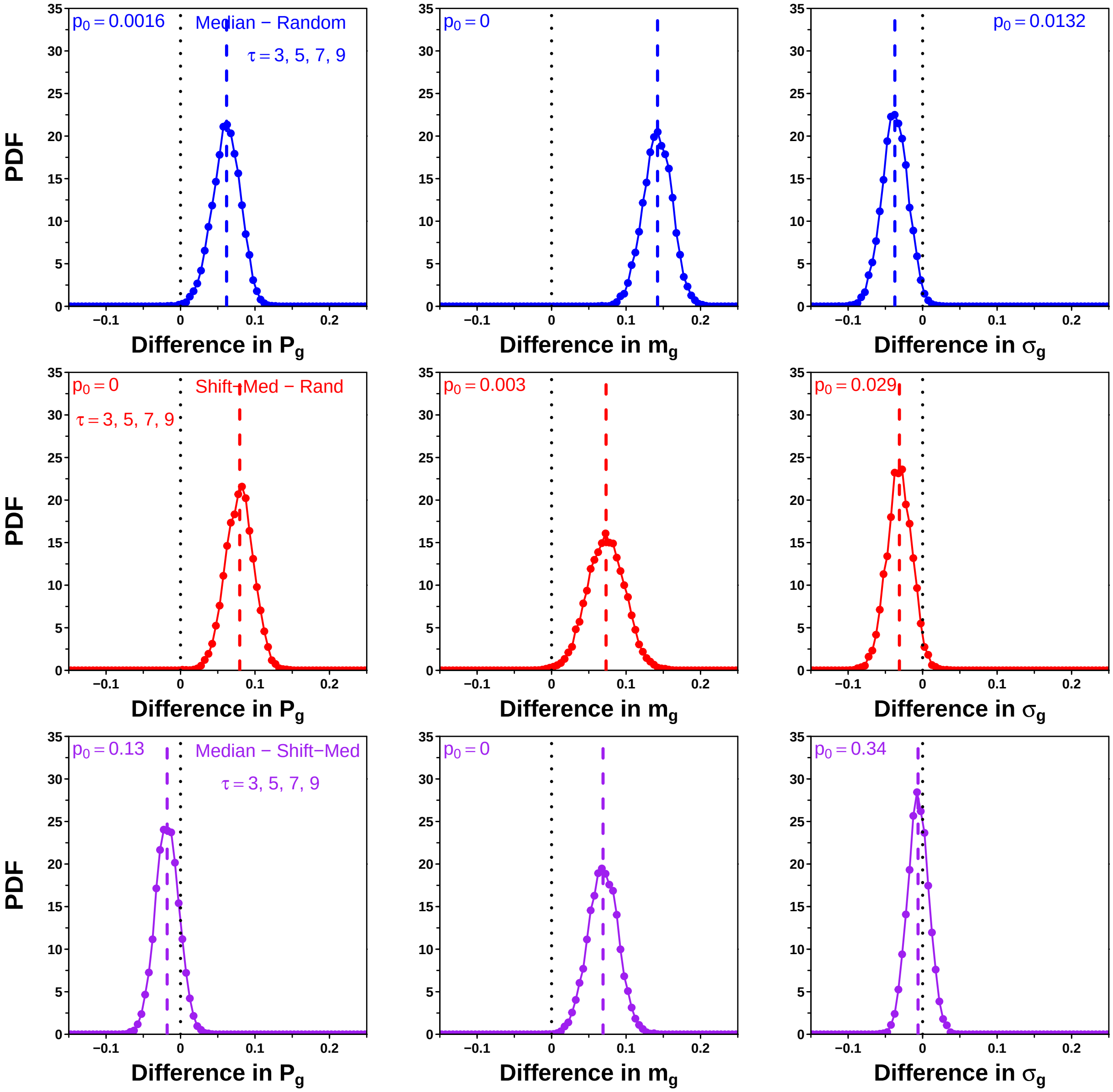

Fig G: **Significance analysis of the differences in P_g, m_g and σ_g between treatments in Fig 4.** Probability density function (PDF) of the bootstrapped differences between the average values (over the intermediate values of τ, i.e., $\tau = 3, 5, 7, 9$) of P_g, m_g and σ_g in the Median and Random treatments (blue), in the Shifted-Median and Random treatments (red), and in the Median and Shifted-Median treatments (purple). P_g and m_g are significantly higher in the Median and Shifted-Median treatments than in the Random treatment. σ_g is significantly lower in the Median and Shifted-Median treatment than in the Random treatment. m_g is significantly higher in the Median treatment than in the Shifted-Median treatment, but not P_g and σ_g. Dashed lines show the median differences, and black dotted lines are the reference value 0.

Model without similarity effect: Figures S8 to S10

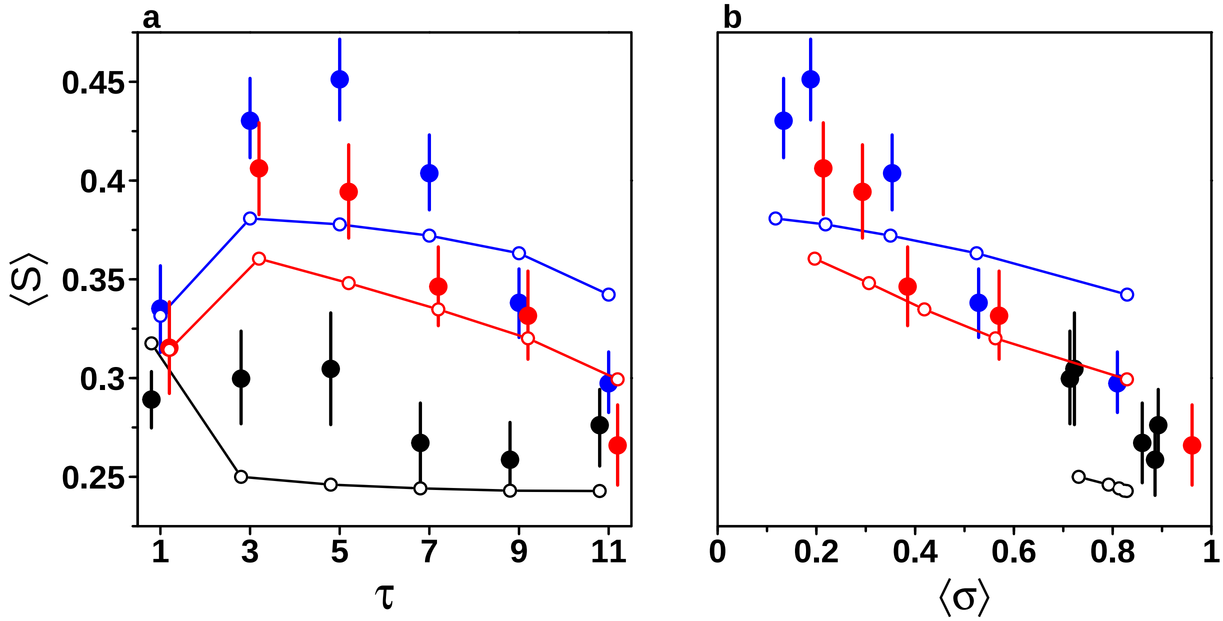

Fig H: **$\langle S \rangle$ against τ and $\langle \sigma \rangle$ for the model without similarity effect.** Average sensitivity to social influence $\langle S \rangle$ against (a) the number of shared estimates τ and (b) their average dispersion $\langle \sigma \rangle$, in the Random (black), Median (blue), and Shifted-Median (red) treatments. Filled dots are the data, while empty dots and solid lines are simulations of the model without the similarity effect. This model underestimates the inverse-U shape in panel a and the decrease of $\langle S \rangle$ with $\langle \sigma \rangle$ in panel b.

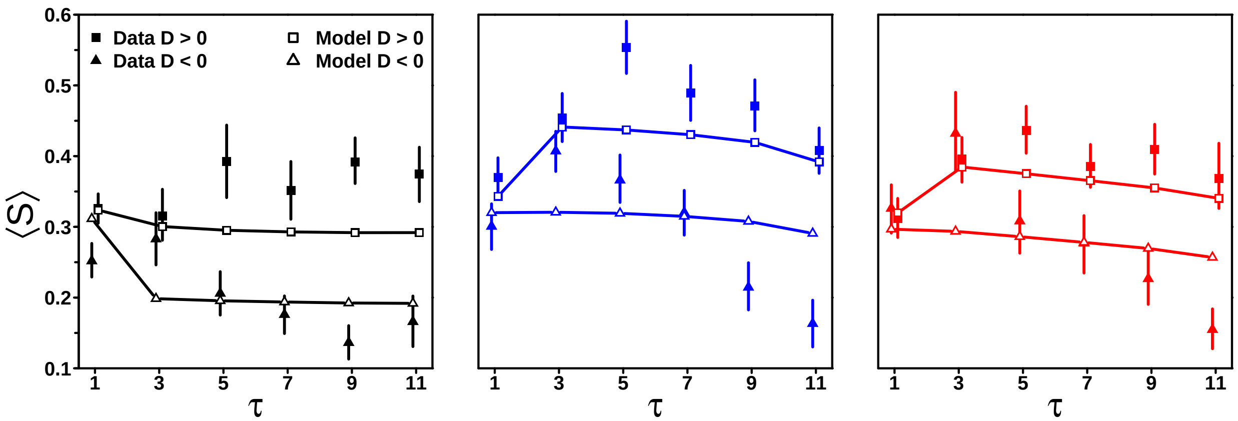

Fig I: $\langle S \rangle$ against τ, when $D < 0$ and $D > 0$, for the model without similarity effect. Average sensitivity to social influence $\langle S \rangle$ against the number of shared estimates τ in the Random (black), Median (blue), and Shifted-Median (red) treatments. The population was separated into subjects' answers where the average social information received M was lower than their personal estimate $X_{\rm p}$ ($D = M - X_{\rm p} < 0$) and subjects' answers where the average social information received was higher than their personal estimate ($D > 0$). Filled symbols represent the data, while solid lines and empty symbols are simulations of the model without the similarity effect. This model is unable to reproduce the empirical results and predicts flatter curves instead.

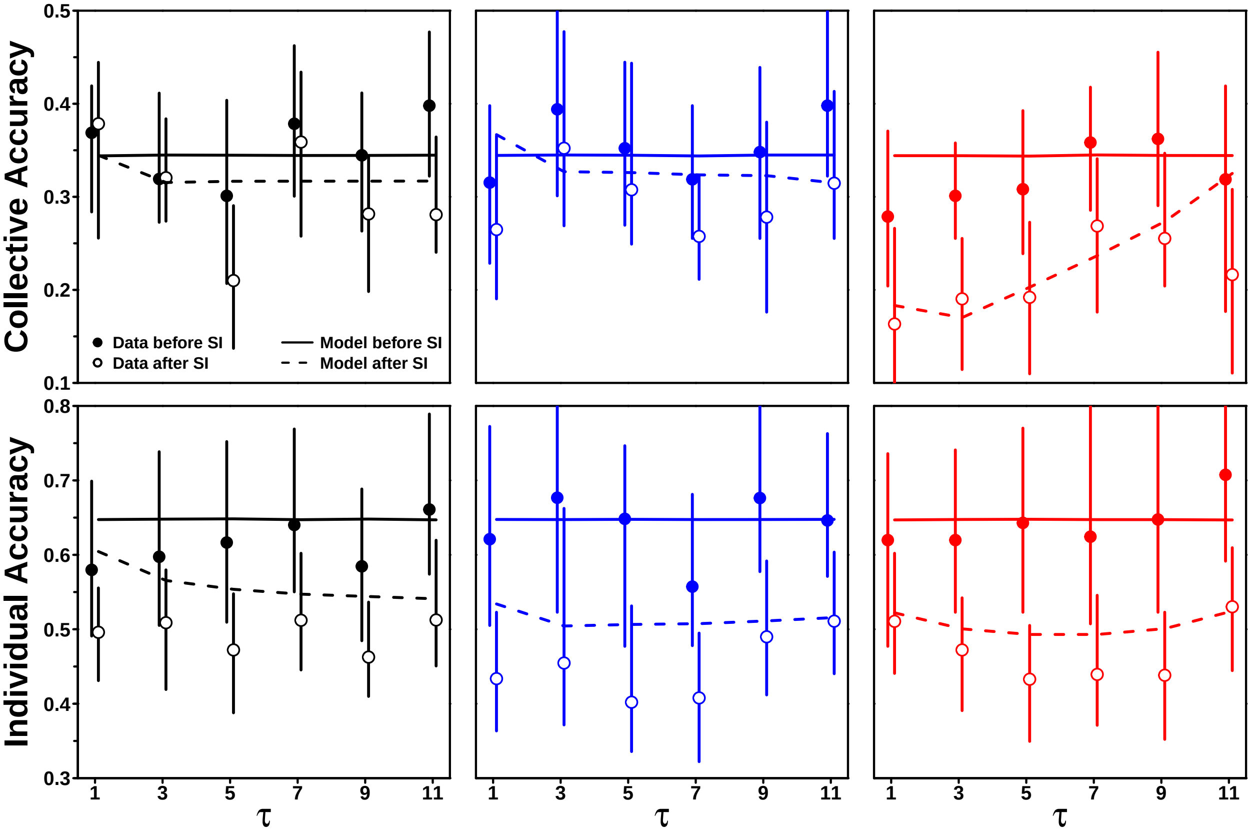

Fig J: Collective and individual accuracy against τ for the model without similarity effect. Collective and individual accuracy against the number of shared estimates τ, before (filled dots) and after (empty circles) social information sharing, in the Random (black), Median (blue), and Shifted-Median (red) treatments. Values closer to 0 indicate higher accuracy. Solid and dashed lines are simulations of the model without the similarity effect, before and after social information sharing, respectively.

Model without asymmetry effect: Figures S11 to S13

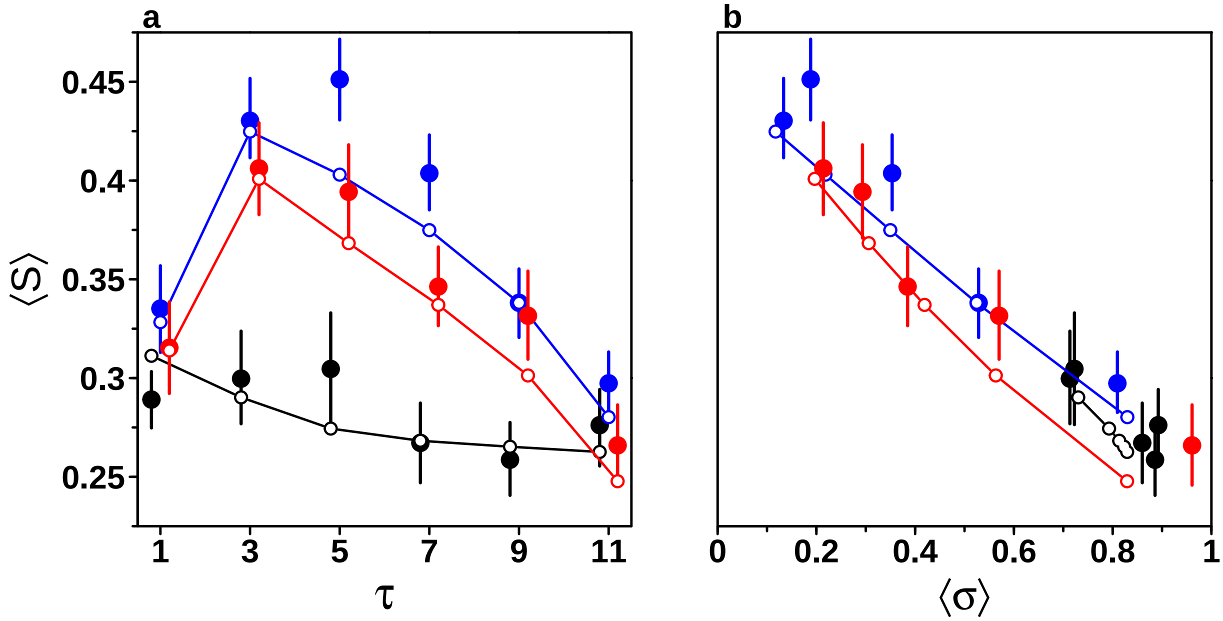

Fig K: **$\langle S \rangle$ against τ and $\langle \sigma \rangle$ for the model without asymmetry effect.** Average sensitivity to social influence $\langle S \rangle$ against (a) the number of shared estimates τ and (b) their average dispersion $\langle \sigma \rangle$, in the Random (black), Median (blue), and Shifted-Median (red) treatments. Filled dots are the data, while empty dots and solid lines are simulations of the model without the asymmetry effect.

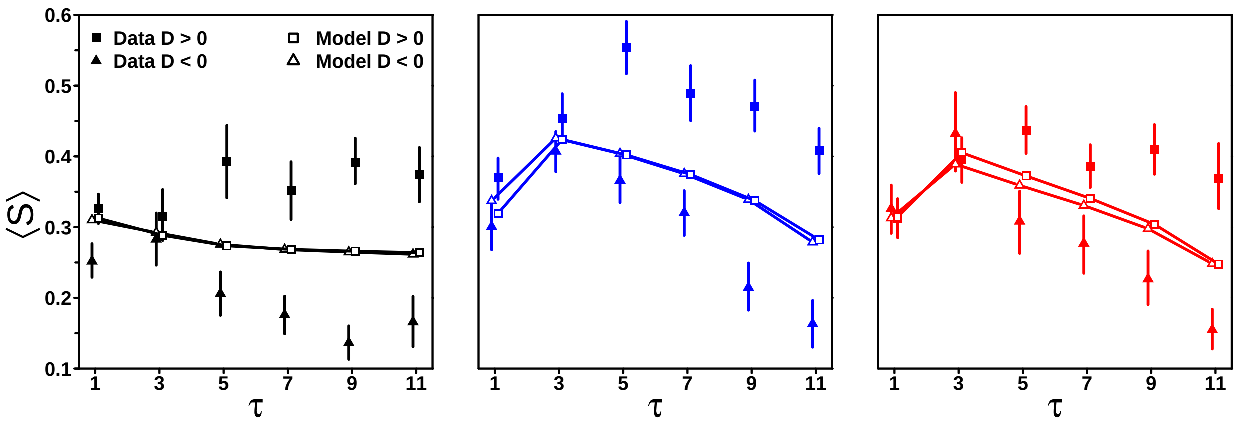

Fig L: $\langle S \rangle$ against τ, when $D < 0$ and $D > 0$, for the model without asymmetry effect. Average sensitivity to social influence $\langle S \rangle$ against the number of shared estimates τ in the Random (black), Median (blue), and Shifted-Median (red) treatments. The population was separated into subjects' answers where the average social information received M was lower than their personal estimate X_{p} ($D = M - X_{\mathrm{p}} < 0$) and subjects' answers where the average social information received was higher than their personal estimate ($D > 0$). Filled symbols represent the data, while solid lines and empty symbols are simulations of the model without the asymmetry effect. This model is unable to reproduce the empirical discrepancy between $\langle S \rangle$ when $D < 0$ and when $D > 0$.

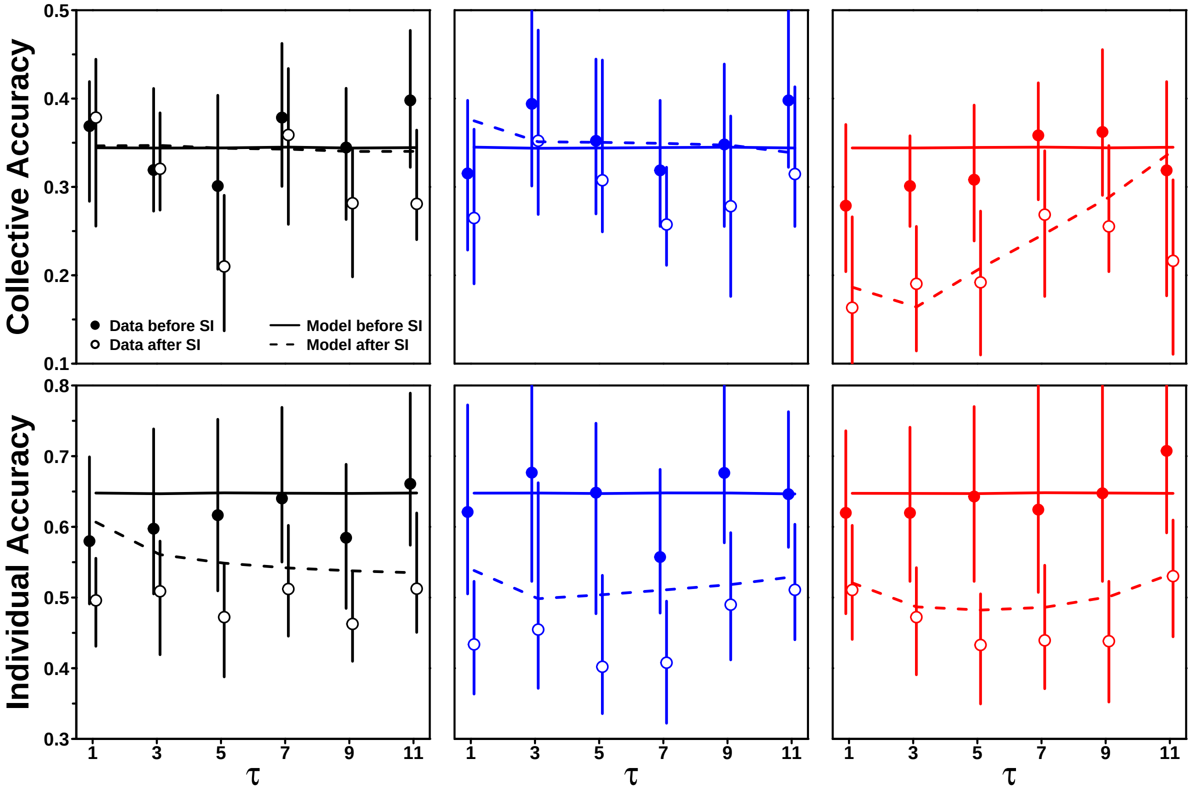

Fig M: Collective and individual accuracy against τ for the model without asymmetry effect. Collective and individual accuracy against the number of shared estimates τ, before (filled dots) and after (empty circles) social information sharing, in the Random (black), Median (blue), and Shifted-Median (red) treatments. Values closer to 0 indicate higher accuracy. Solid and dashed lines are simulations of the model without the asymmetry effect, before and after social information sharing, respectively. This model is unable to reproduce the improvement in collective accuracy in the Random and Median treatments.

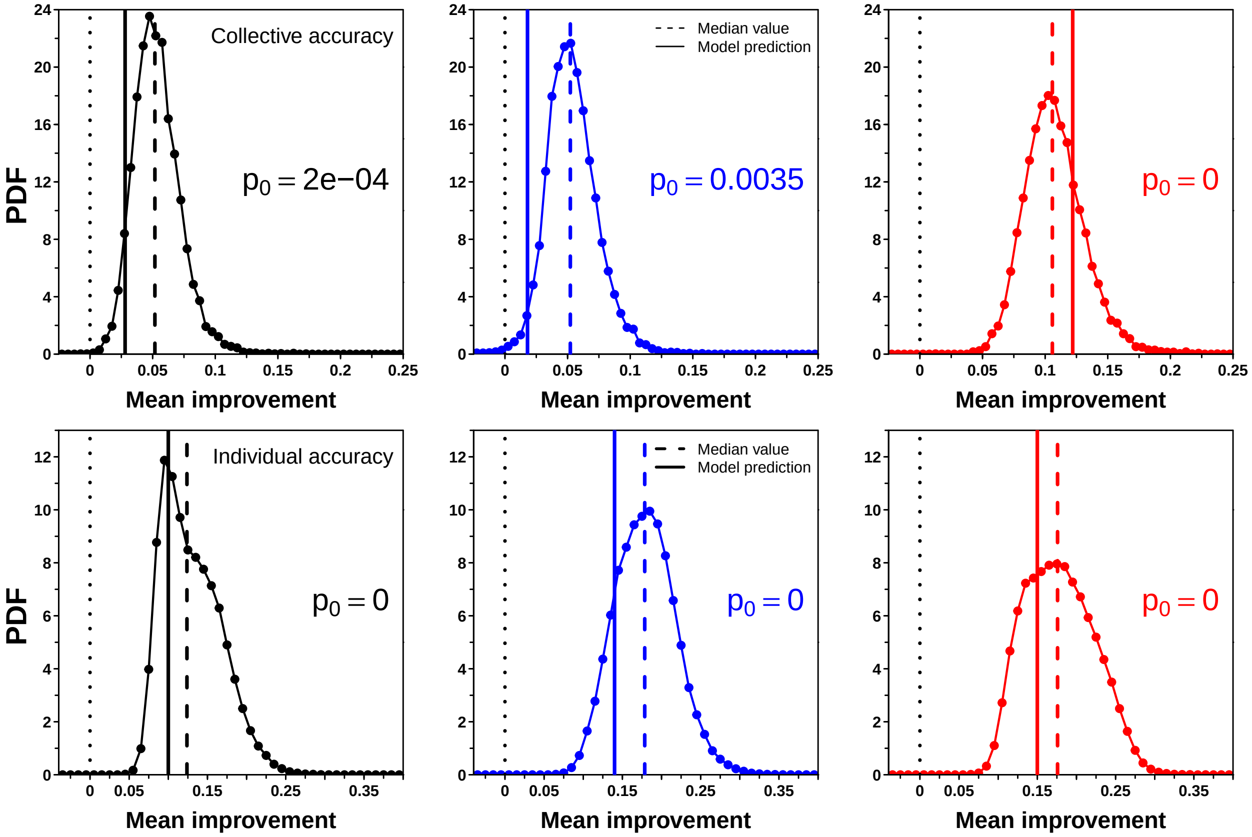

Fig N: **Significance analysis of the improvement in collective and individual accuracy in Fig 12.** Probability density function (PDF) of the bootstrapped average (over all values of τ) improvements (see proper definition of improvements in the main text) in collective (top row) and individual (bottom row) accuracy, in the Random (black), Median (blue) and Shifted-Median (red) treatments. Improvement in collective accuracy is significant but not large in the Random and Median treatments, while it is substantial and highly significant in the Shifted-Median treatment. Improvements in individual accuracy are highly significant in all treatments. Dashed lines show the median value of the bootstrapped mean improvements, and solid lines the values predicted by the model. The black dotted lines are the reference value 0.

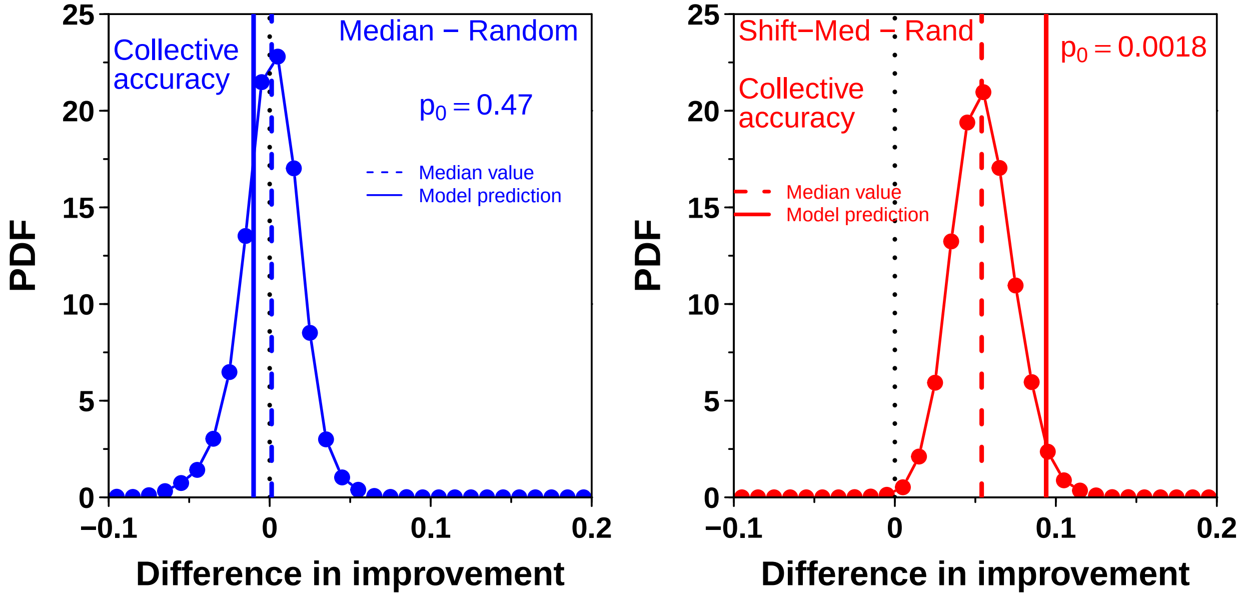

Fig O: **Significance analysis of the difference in improvement in collective accuracy between treatments in Fig 12.** Probability density function (PDF) of the bootstrapped differences in average (over all values of τ) improvement in collective accuracy between the Median and Random treatments (blue), and the Shifted-Median and Random treatments (red). Improvements in the Median and Random treatments are not significantly different. However, the improvement is significantly higher in the Shifted-Median treatment than in the Random treatment. Dashed lines show the median differences in improvement, and solid lines the values predicted by the model. The black dotted lines are the reference value 0.

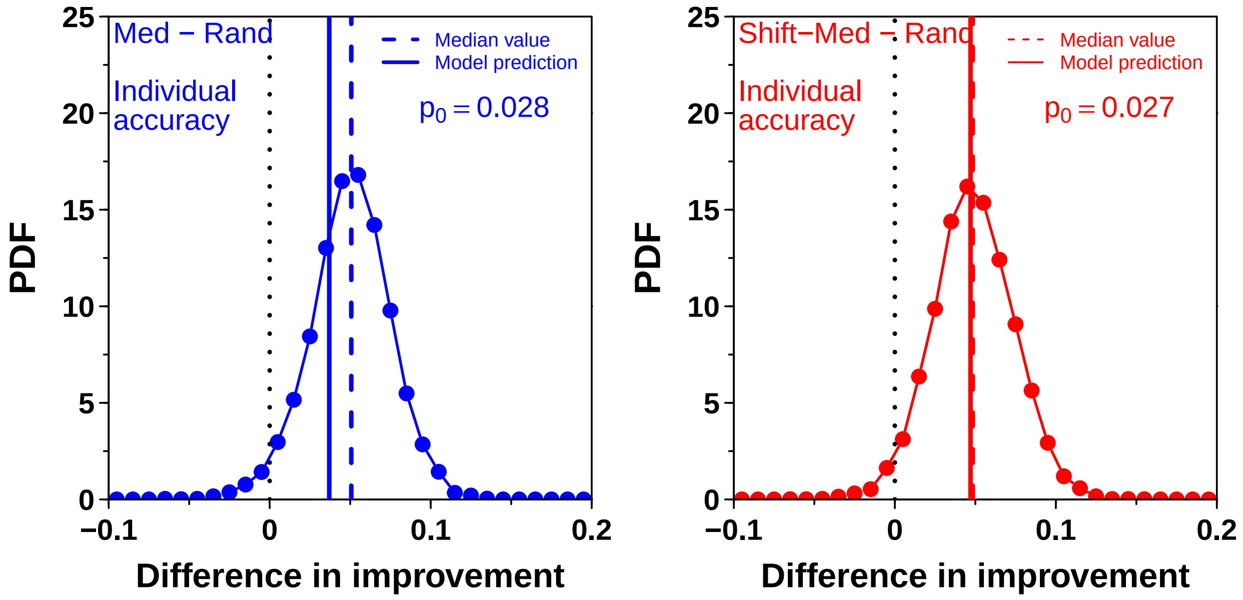

Fig P: **Significance analysis of the difference in improvement in individual accuracy between treatments in Fig 12.** Probability density function (PDF) of the bootstrapped differences in average (over all values of τ) improvement in individual accuracy between the Median and Random treatments (blue), and the Shifted-Median and Random treatments (red). Improvements in individual accuracy are mildly (but significantly) higher in the Median and Shifted-Median treatments than in the Random treatment. Dashed lines show the median differences in improvements, and solid lines the values predicted by the model. The black dotted lines are the reference value 0.

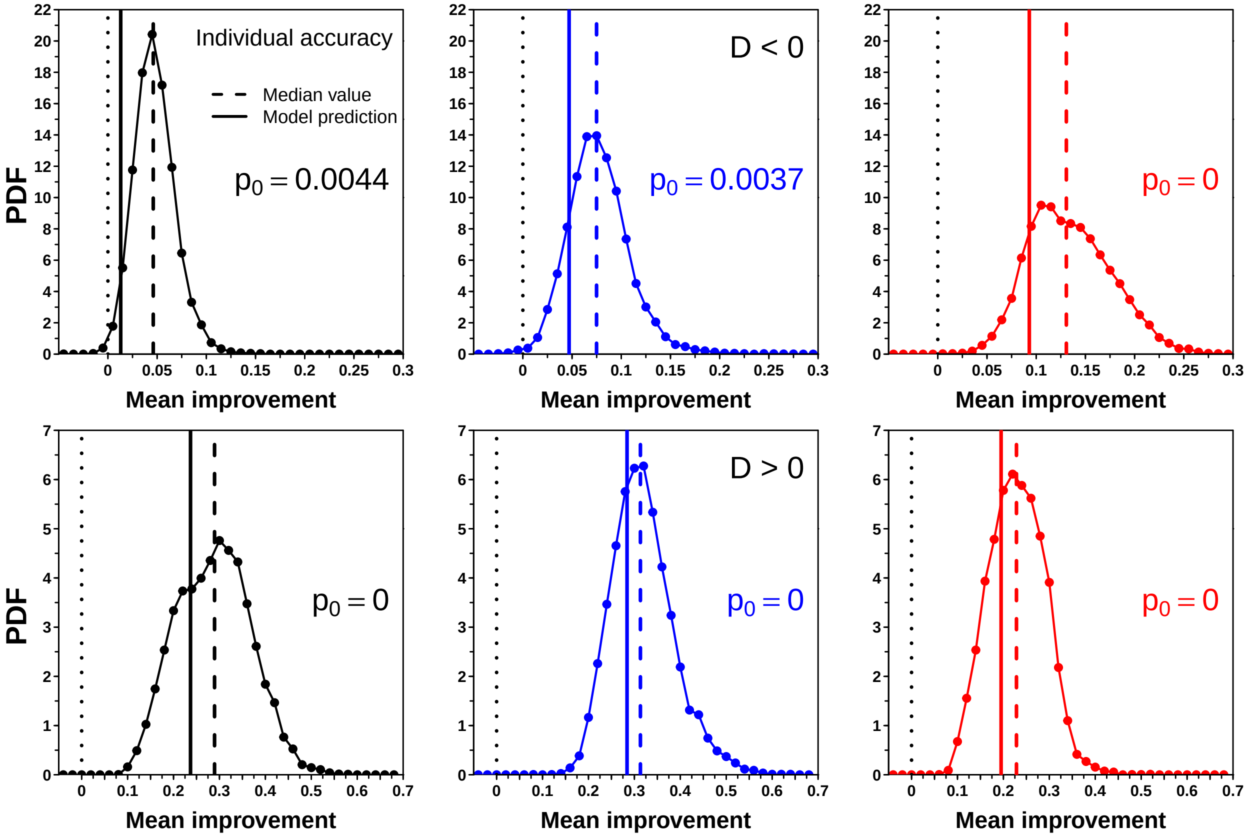

Fig Q: **Significance analysis of the improvement in individual accuracy in Fig 13.** Probability density function (PDF) of the bootstrapped average (over all values of τ) improvements in individual accuracy when $D < 0$ (top row) and $D > 0$ (bottom row), in the Random (black), Median (blue) and Shifted-Median (red) treatments. Improvements are mild but significant when $D < 0$ in the Random and Median treatments, and strong and highly significant in all four other cases. Dashed lines show the median value of the bootstrapped mean improvements, and solid lines the values predicted by the model. The black dotted lines are the reference value 0.

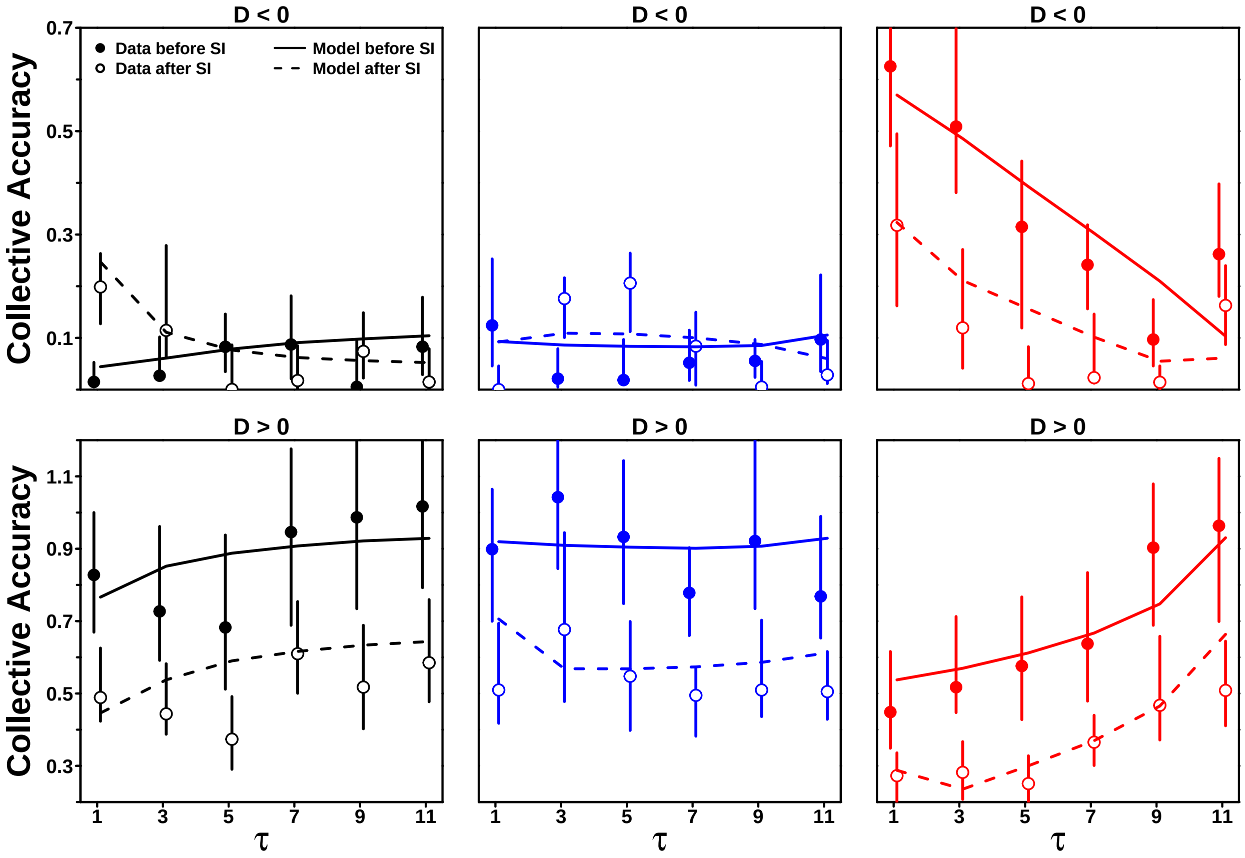

Fig R: Collective accuracy against τ when $D < 0$ and when $D > 0$. Collective accuracy against the number of shared estimates τ, before (filled dots) and after (empty circles) social information sharing, in the Random (black), Median (blue) and Shifted-Median (red) treatments. The population was separated into subjects' answers where the average social information received M was lower than their personal estimate X_p ($D = M - X_\mathrm{p} < 0$) and subjects' answers where the average social information received was higher than their personal estimate ($D > 0$). Solid and dashed lines are model simulations before and after social information sharing, respectively.

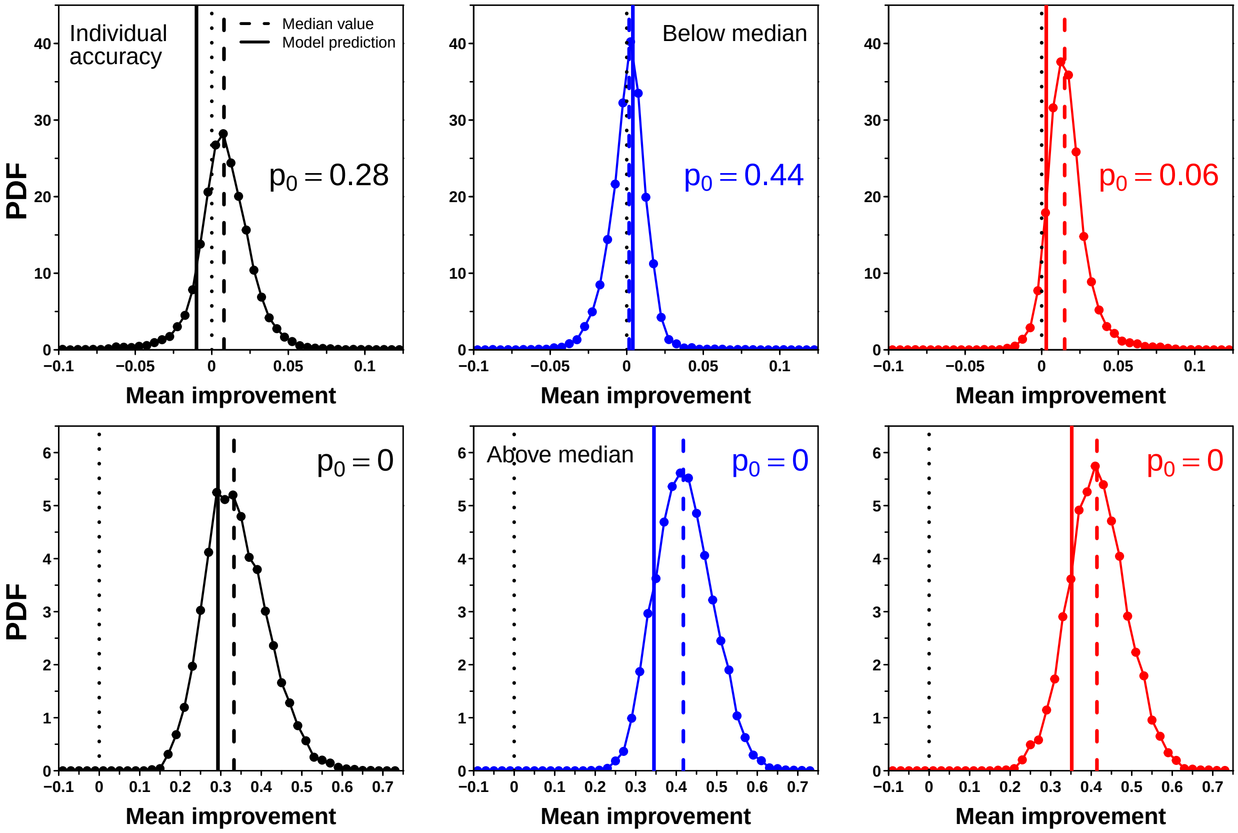

Fig S: **Significance analysis of the improvements in individual accuracy in Fig 14.** Probability density function (PDF) of the bootstrapped average (over all values of τ) improvements in individual accuracy in the Random (black), Median (blue) and Shifted-Median (red) treatments. In each condition, the subjects' answers were separated according to their corresponding value of S with respect to the median of S. The top row shows the below median case, and the bottom row the above median case. We observe no significant improvements in individual accuracy in the Random and Median treatments in the below-median case, and a mild improvement (although barely significant) in the Shifted-Median treatment in this case. Improvements are, however, substantial and highly significant in the above-median case. Dashed lines show the median improvements, and solid lines the values predicted by the model. The black dotted lines are the reference value 0.

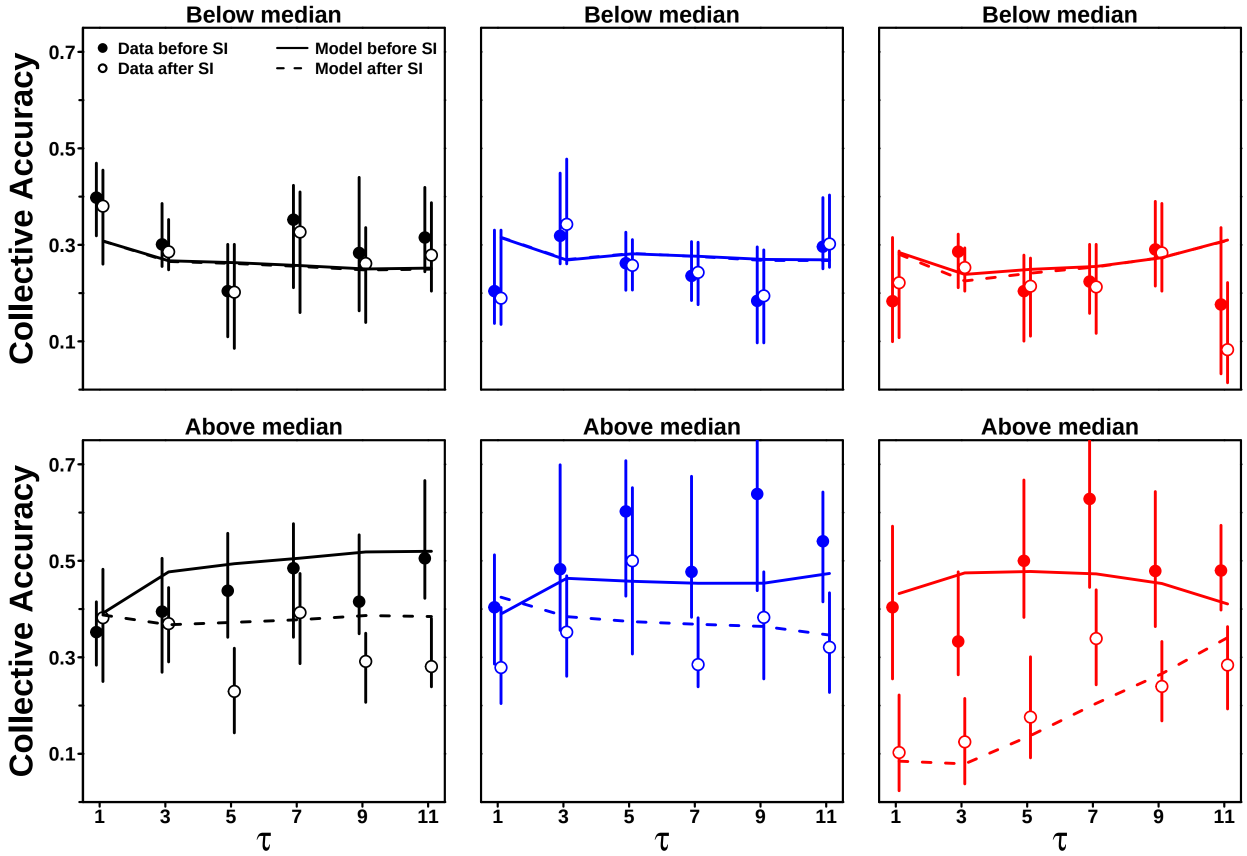

Fig T: Collective accuracy against τ when S is below and above Median(S). Collective accuracy against the number of shared estimates τ, before (filled dots) and after (empty circles) social information sharing, in the Random (black), Median (blue), and Shifted-Median (red) treatments. In each condition, the subjects' answers were separated according to their corresponding value of S with respect to the median of S. Solid and dashed lines are model simulations before and after social information sharing, respectively. When S is lower than the median, the subjects tend to keep their initial estimate, and individual accuracy therefore does not change. When S is higher than the median, the subjects tend to compromise with the social information, resulting in high improvements.

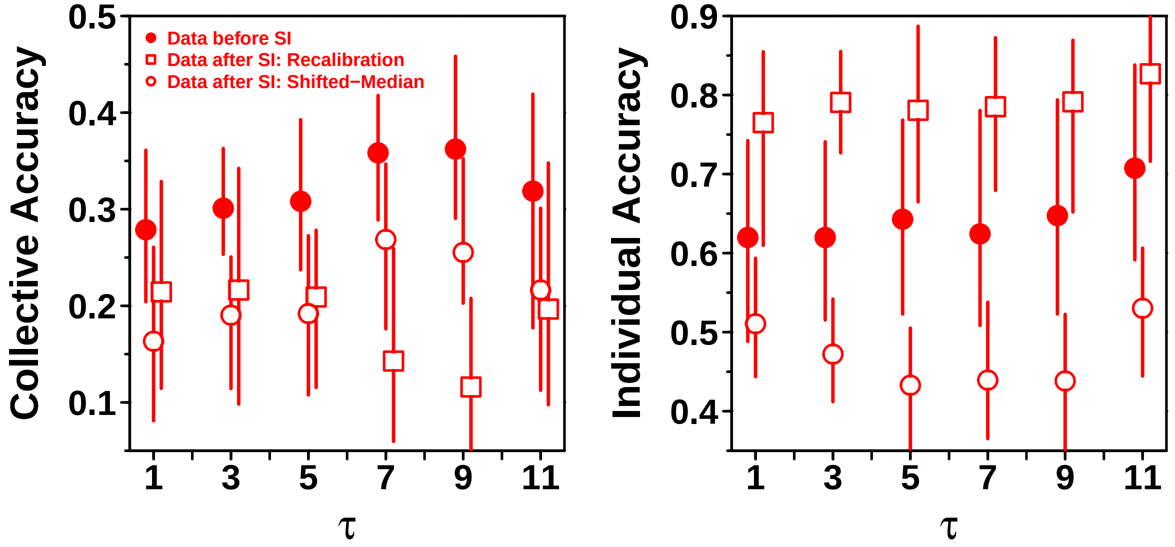

Fig U: **Collective and individual accuracy against τ in the Shifted-Median treatment compared to a simple recalibration of initial estimates.** Collective and individual accuracy against the number of shared estimates τ, before (filled dots) and after (empty circles) social information sharing, in the Shifted-Median treatment. Squares denote the results of the recalibration of personal estimates (see Discussion for details). Collective accuracy improves similarly with this recalibration method as in the Shifted-Median treatment. However, individual accuracy decays with the recalibration method, while it improves substantially in the Shifted-Median treatment.

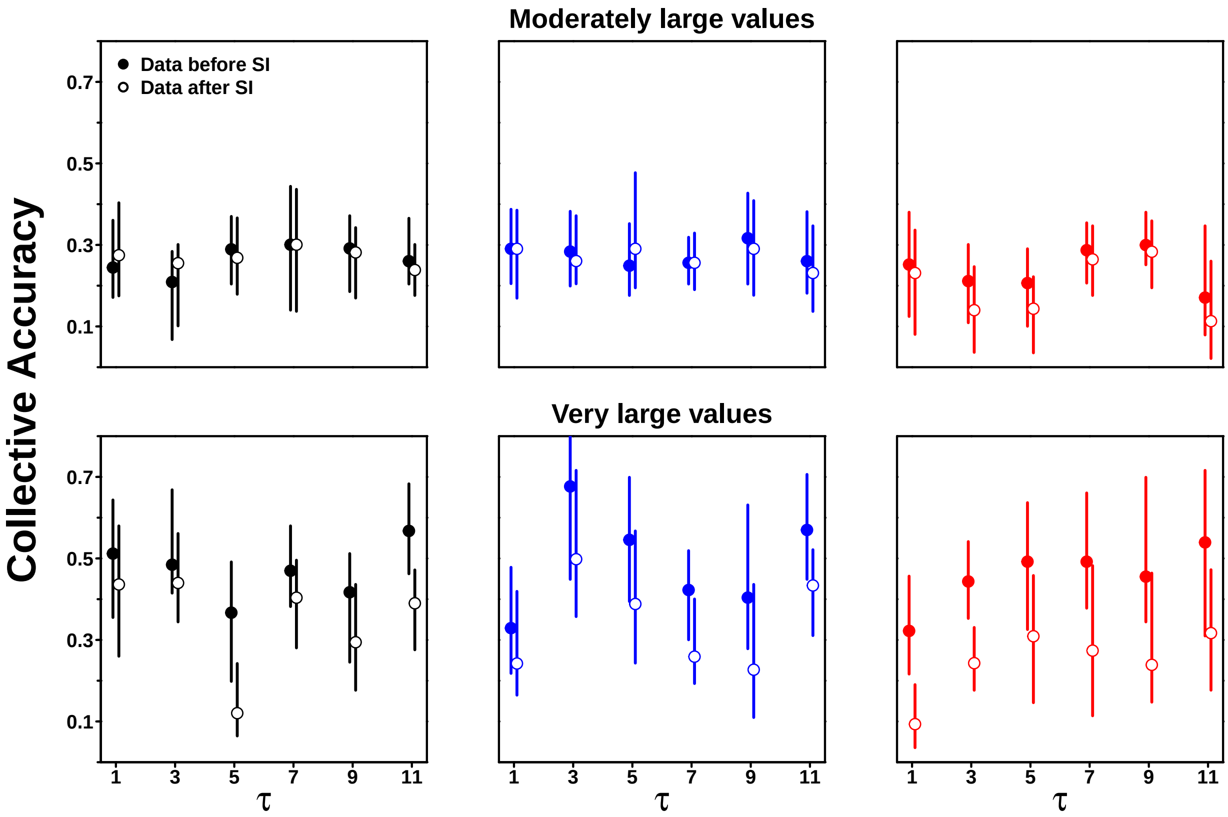

Fig V: **Collective accuracy against τ for moderately large and very large quantities.** Collective accuracy against the number of shared estimates τ, before (filled dots) and after (empty circles or squares) social information sharing, in the Random (black), Median (blue) and Shifted-Median (red) treatments. Top/bottom panels indicate the results of the half of our questions with lowest/highest true values. Before social information sharing, collective accuracy is higher (i.e., closer to 0) for moderately large values than for very large values, but improves more in the latter than in the former.

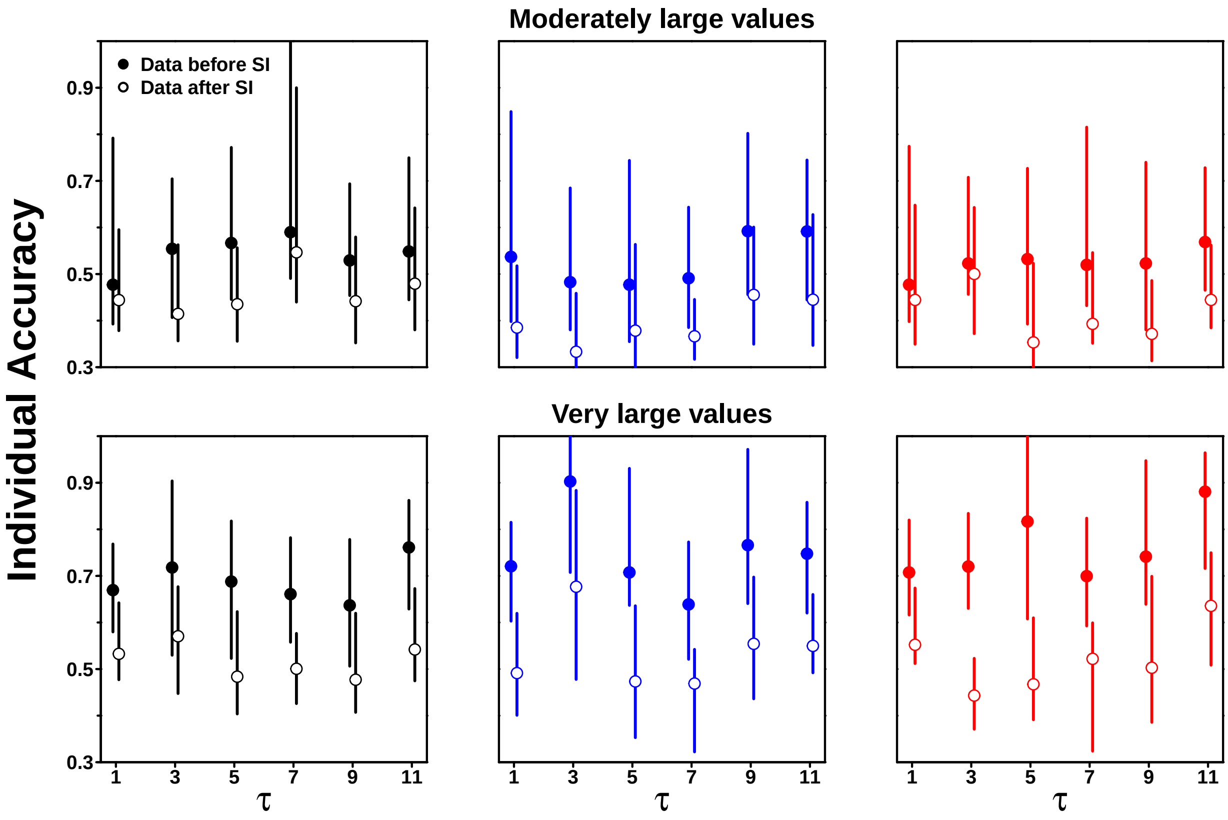

Fig W: **Individual accuracy against τ for moderately large and very large quantities.** Individual accuracy against the number of shared estimates τ, before (filled dots) and after (empty circles or squares) social information sharing, in the Random (black), Median (blue) and Shifted-Median (red) treatments. Top/bottom panels indicate the results of the half of our questions with lowest/highest true values. Before social information sharing, individual accuracy is higher (i.e., closer to 0) for moderately large values than for very large values, but improves more in the latter than in the former.

4 Supplementary tables

Treatment	τ	$D > 0$ (%)	$D < 0$ (%)	Standard deviation
Random	1	50.4	49.6	1.9
Random	3	52.2	47.8	2.3
Random	5	52.9	47.1	2.0
Random	7	52.0	48.0	1.7
Random	9	47.9	52.1	2.2
Random	11	52.6	47.4	1.8
Median	1	49.9	50.1	0.6
Median	3	49.5	50.5	1.5
Median	5	45.5	54.5	2.1
Median	7	49.3	50.7	1.6
Median	9	48.0	52.0	1.4
Median	11	54.7	45.3	2.1
Shifted-Median	1	76.4	23.6	2.0
Shifted-Median	3	71.3	28.7	1.9
Shifted-Median	5	67.3	32.7	2.1
Shifted-Median	7	63.8	36.2	2.2
Shifted-Median	9	57.6	42.4	2.0
Shifted-Median	11	52.0	48.0	2.3

Table A: Distribution of cases when the social information provided to an individual was higher ($D > 0$) or lower ($D < 0$) than their personal estimate, for each combination of treatment and number of estimates received τ. The proportions are roughly equal in the Random (with a slight dominance of the $D > 0$ cases) and Median (with a slight dominance of the $D < 0$ cases) treatments, while the social information is much more often higher ($D > 0$) in the Shifted-Median treatment. This trend decreases as τ increases until proportions are roughly equal at $\tau = 11$.

Figure	Description	Treatment	Goodness-of-Fit	Relative error
Fig 5	$\langle\sigma\rangle$ VS τ	Random	0.57	0.07
Fig 5	$\langle\sigma\rangle$ VS τ	Median	0.59	0.06
Fig 5	$\langle\sigma\rangle$ VS τ	Shifted-Median	0.63	0.08
Fig 10a	$\langle S\rangle$ VS τ	Random	1.11	0.07
Fig 10a	$\langle S\rangle$ VS τ	Median	1.28	0.05
Fig 10a	$\langle S\rangle$ VS τ	Shifted-Median	0.52	0.03
Fig 10b	$\langle S\rangle$ VS $\langle\sigma\rangle$	Random	0.84	0.06
Fig 10b	$\langle S\rangle$ VS $\langle\sigma\rangle$	Median	1.4	0.06
Fig 10b	$\langle S\rangle$ VS $\langle\sigma\rangle$	Shifted-Median	0.57	0.04
Fig 11	$\langle S\rangle$ VS τ $(D > 0)$	Random	1.35	0.14
Fig 11	$\langle S\rangle$ VS τ $(D > 0)$	Median	1.7	0.14
Fig 11	$\langle S\rangle$ VS τ $(D > 0)$	Shifted-Median	1.19	0.11
Fig 11	$\langle S\rangle$ VS τ $(D < 0)$	Random	1.7	0.18
Fig 11	$\langle S\rangle$ VS τ $(D < 0)$	Median	1.26	0.13
Fig 11	$\langle S\rangle$ VS τ $(D < 0)$	Shifted-Median	1.18	0.14
Fig 12	Collective accuracy before SI	Random	0.43	0.09
Fig 12	Collective accuracy before SI	Median	0.36	0.08
Fig 12	Collective accuracy before SI	Shifted-Median	0.54	0.1
Fig 12	Collective accuracy after SI	Random	0.66	0.13
Fig 12	Collective accuracy after SI	Median	0.7	0.13
Fig 12	Collective accuracy after SI	Shifted-Median	0.56	0.17
Fig 12	Individual accuracy before SI	Random	0.42	0.06
Fig 12	Individual accuracy before SI	Median	0.39	0.05
Fig 12	Individual accuracy before SI	Shifted-Median	0.25	0.04
Fig 12	Individual accuracy after SI	Random	0.91	0.09
Fig 12	Individual accuracy after SI	Median	0.79	0.11
Fig 12	Individual accuracy after SI	Shifted-Median	0.43	0.06
Fig 13	Individual accuracy before SI $(D < 0)$	Random	0.76	0.077
Fig 13	Individual accuracy before SI $(D < 0)$	Median	1.08	0.14
Fig 13	Individual accuracy before SI $(D < 0)$	Shifted-Median	0.69	0.13
Fig 13	Individual accuracy after SI $(D < 0)$	Random	1.01	0.11
Fig 13	Individual accuracy after SI $(D < 0)$	Median	1.24	0.21
Fig 13	Individual accuracy after SI $(D < 0)$	Shifted-Median	0.98	0.17
Fig 13	Individual accuracy before SI $(D > 0)$	Random	0.5	0.1
Fig 13	Individual accuracy before SI $(D > 0)$	Median	0.62	0.09
Fig 13	Individual accuracy before SI $(D > 0)$	Shifted-Median	0.45	0.08
Fig 13	Individual accuracy after SI $(D > 0)$	Random	0.74	0.11
Fig 13	Individual accuracy after SI $(D > 0)$	Median	0.84	0.13
Fig 13	Individual accuracy after SI $(D > 0)$	Shifted-Median	0.58	0.1
Fig 14	Individual accuracy before SI (Below median)	Random	0.46	0.06
Fig 14	Individual accuracy before SI (below median)	Median	1.63	0.13
Fig 14	Individual accuracy before SI (below median)	Shifted-Median	1.1	0.14
Fig 14	Individual accuracy after SI (below median)	Random	0.7	0.09
Fig 14	Individual accuracy after SI (below median)	Median	1.54	0.15
Fig 14	Individual accuracy after SI (below median)	Shifted-Median	1.07	0.14
Fig 14	Individual accuracy before SI (above median)	Random	0.38	0.05
Fig 14	Individual accuracy before SI (above median)	Median	0.43	0.08
Fig 14	Individual accuracy before SI (above median)	Shifted-Median	0.71	0.12
Fig 14	Individual accuracy after SI (above median)	Random	0.83	0.11
Fig 14	Individual accuracy after SI (above median)	Median	0.78	0.1
Fig 14	Individual accuracy after SI (above median)	Shifted-Median	0.45	0.1

Table B: **Goodness-of-Fit and relative error between the data and the model.** The Goodness-of-Fit (GoF; see main text), defined as $\sqrt{\frac{1}{N_\tau}\sum_\tau \frac{(O_\tau - M_\tau)^2}{{C_\tau}^2}}$, is analogous to the reduced χ-squared (where errors follow Gaussian distributions), and compares the accuracy of the model predictions to the observed fluctuations in the data. Reasonably accurate model predictions or fits should lead to a value of the GoF of order 1, which is the case in all our figures. The relative error, defined as $\frac{1}{N_\tau}\sum_\tau \frac{|O_\tau - M_\tau|}{|M_\tau|}$, is also provided.
