# Supplementary material for: Crowd control: Reducing individual estimation bias by sharing biased social information
Source: PLoS Comput Biol. 2021 Nov 29;17(11):e1009590. doi: 10.1371/journal.pcbi.1009590 (PMC8659305; doi:10.1371/journal.pcbi.1009590)
Supplement: Attachment — Submitted filename: Review_PCOMPBIOL_D2000065.pdf [file pcbi.1009590.s002.pdf]

Review of manuscript PCOMPBIOL-D-20-00065

Title: Debiasing the crowd: selectively exchanging social information improves collective

decision-making

General Comment: I will state first that I am not in the area of social choice, and my expertise is more in sequential design of experiments and Bayesian Optimization, which are techniques that could have an important role in the work presented by the authors.

The paper looks into the problem of underestimation bias in a setting with a group of people is asked to provide an estimate and subsequently is provided with several estimations from the remainder of the group (a subset). The authors use three mechanism to select how to exchange information: a random mechanism, a median driven mechanism, and a shifted median exchange, which considers the overestimation bias and compensates for it using a factor γ. The authors proceed optimizing this factor and identify three modeling factors to be considered to study the impact of the amount of exchanged information onto the individual accuracy and collective accuracies. These aspects are *herd, asymmetry, and similarity*.

The paper was an incredibly interesting read, I believe the authors make a good case on the contribution of their work compared with the available literature, which appear to be herd, asymmetry and similarity. I can appreciate the modeling value in this. However, there are some aspects which I believe the authors should clarify to make the paper contribution clearer and, possibly, stronger.

- What is the effect of the specific population being chosen? Are there aspects of the model that can indicate different outcomes in terms of the accuracy, based on the population? Can we have populations that are over and underestimating?
- It is unclear whether the theory being developed in this paper may apply to non quantitative cases. The authors refer in multiple places to recommendation systems, but more details should be provided on how the very idea of underestimation bias that the authors use to define the coefficient γ, can be extended to cases where no quantitative measures are available.

In the following some more detailed comments are provided.

Detailed comments:

Comment #1: On page 4-5, the authors discuss the experiment design and the over-compensation mechanism. There is an underlying message in this part that starting with bad estimated we can improve accuracy by mixing the bad estimates in an effective way. This concept is the heart and soul of *boosting* in statistics and machine learning. Do the authors think there is somewhat some relevance of the boosting literature that could lead to a statistical justification for the choice of the size of the set and the weight to give to each of the selected information?

Comment #2: Always related to the experiment, the authors select a homogeneous population age-wise. What it the impact of this choice? When we talk about news and social media the population will be way more heterogenous. Could this represent an issue?

Comment #3: The authors claim that γ can be derived without knowledge of the true value of the measure. However, it was not clear to me how $\gamma = 0.9$ was derived. It seems to me that $\gamma = 0.9$ requires

to be set the knowledge of the true value. Do the authors mean that since they optimize for γ they actually do not need to assume a value for the truth?

Comment #4: page 6, line 161-162. Could the observed effects be associated with the specific choice of the source be shared? Is there a possibility to identify "better evaluators" that have higher individual accuracy that the others can tend to? If not would that be possible to bias towards? This is very common in recommendation systems or crowd sourced search engines, where people that post responses are evaluated by the algorithm and if they are consistently right, they get "higher weight". Can this mechanism be considered? Or here we can specifically solve underestimation bias?

Comment #5: page 6, line 161. The authors say that the shifted median collective accuracy is basically concave in the amount of data being exchanged. However, do the authors mean that the relative improvement of shifted against the rest is decreasing with the number of data being shared?

Comment #6: Shouldn't the y-axis in Figure 2 be the relative improvement and not the absolute improvement?

Comment #7: Page 10, line 232. So do the authors mean that the herding effect is a by-product of the model? Can the authors expand this explanation?

Comment #8: Optimal γ. The fact that the authors are finding a $\gamma \neq 0.9$ and could be substantially different, may be a disprove of the logarithmic perception model assumption?

Comment #9: Figure 5.b. I find quite interesting that the size of the group and the value of τ^* (i.e., the best amount of information to share for a specific group size) is very different between collective and individual accuracy. Does this imply that these two are in trade off? The authors seem to suggest at the beginning of the paper that this is not the case, but could they dedicate some explanation to this fact?

Comment #10: Discussion. A possible limitation is that the very concept of underestimation bias appears to be inherently connected to having quantitative estimates, how can this concept for example be extended to cases such as ranking and recommendation systems? I have no problem with the fact that the authors may be looking into a very specific type of bias, but then I think it would be fair to better qualify the statements in the conclusions about the future applications.
