# Supplementary material for: Crowd control: Reducing individual estimation bias by sharing biased social information
Source: PLoS Comput Biol. 2021 Nov 29;17(11):e1009590. doi: 10.1371/journal.pcbi.1009590 (PMC8659305; doi:10.1371/journal.pcbi.1009590)
Supplement: Attachment — Submitted filename: PCOMPBIOL-D-20-00065_Comments_to_Authors.pdf [file pcbi.1009590.s003.pdf]

This report refers to PLOS Computational Biology submission PCOMPBIOL-D-20-00065, “Debiasing the crowd: selectively exchanging social information improves collective decision-making”. After reviewing the manuscript in detail and performing an independent search to verify some of the authors’ claims regarding the novelty of their present work, it is my belief that the paper makes worthwhile contributions to the theory of (human) collective decision-making. However, the manuscript lacks key details in various parts, which make it difficult to contextualize and quantify the true impact of these contributions. Upon the considerations and suggested edits listed below, it is my opinion that the paper should undergo a major revision to address the considerations listed in the below paragraphs.

The key considerations are as follows:

- <u>Missing details about the estimation activities</u>. The estimation experiments performed as part of this research are listed in the appendix, but there is little information about them within the body of the paper. This lack of detail should be addressed because the principal message/applicability of the paper should be self-contained and, more importantly, because the underestimation bias is not relevant to every type of estimation task. Indeed, there are a number of cognitive overestimation biases—estimating the likelihood of certain events (e.g., see reference [27] of manuscript), travel times, phantom patterns in data, etc.—that would be exacerbated by shifting up the collective estimate. The authors are thus asked to provide the general characteristics of their estimation activities up front and from these to describe the types of estimation activities for which their approach is likely to be most relevant later in the paper. They should also mention other estimation tasks where it may not be advisable to apply their techniques and/or where a shift of the median in the opposite direction may be the preferred option. On a related note, the authors are asked to specify the equation for the median shift up front (the derivation can remain in the appendix) and to specify for what types of activities the shift is expected to be applicable.

- <u>Novelty of introduced effects</u>. The authors introduce different mechanisms underlying the integration of multiple pieces of social information. To measure the effectiveness of these mechanisms, the authors introduce *effects* which may be known by other names in other fields and/or which may clash with existing terminology. For instance, the introduced *herding effect* seems to be related to the well-known *bandwagon effect*, which is the tendency of individuals to follow what others are doing, especially when a lot of other people are doing it (Simon, 1954). Moreover, a *herd(ing) effect* exists in psychology (Moldovan, S. 2010), economics (Banerjee, 1992) and epidemiology (John and Samuel, 2000). The authors are asked evaluate the appropriateness of their effect names and to clarify any relevant similarities/differences with other existing similar concepts.

- <u>Accuracy of estimates</u>. There two issues with the way that the accuracy of estimates is presented that obfuscates what was achieved in the experiments.
  First, the magnitude of the ground truth T for the questions that were asked is widely varying (from 100 to billions). Hence, aggregating the errors over all the questions—which is what I believe is done to summarize the results, but please correct me if I’m misinterpreting this—would naturally lead to an overrepresentation of the errors from the questions with the largest magnitudes, even if the reported statistics are the logs of the aggregate statistics. Here I would suggest dividing the questions into groups based on magnitude ranges of T (or providing results of individual questions) and separating the

results accordingly and to report the absolute deviation of the collective and individual estimates to *T* in addition to the logarithms of these quantities.
Second, the relative improvement calculation seems to be used in an absolute sense rather than in a relative sense. Intuitively, I would expect this quantify to be calculated based on the difference between the E_S and E_P divided by E_P. The authors are asked to specify how they calculate relative improvement and potentially modify this term to emphasize that it is based on the incremental similarity to *T* rather than to the change from E_P.

- Relevance of effects. On page 10, the authors claim that the measured effects act independently to improve the collective improvement patterns. How did they determine this? Did they do so by considering each of them separately and their different combinations? Such a design would allow a definitive ranking of the importance of the effects, but I believe the text refers to just one sequence of adding them one after another.

- Other minor edits. Overall, the paper is well written, but some minor edits should be made to enhance clarity, including:

    - Pg. 1, lines 19-20; pg. 2, lines 34-36: The authors claim that prior knowledge on the underestimation bias can be leveraged to select the estimates which, are most likely to counter its effects. As far as I can tell, subjects may not have prior knowledge of the underestimation bias. In the executed experiments, where subjects told about the underestimation bias is? Additionally, did they know that some of the social information received would be shifted upwards to counteract the bias?

    - Pg. 4, lines 95 and 105. Consider changing “estimates which” to “estimates of which”. I believe the latter captures the authors’ intended meaning; the former phrasing is quite confusing/awkward.

    - Pg. 6 lines 147-149. The difference between the formulas for collective accuracy and individual accuracy is not clear. The norm symbol |.| is placed outside the full expression for the former but inside the median function for the latter, but it is hard to see what the exact implication of this is since it is not stated what norm is being used (and what are the ranges of the indices operated over wihin the equations). Please clarify by stating what norm being used or alternatively by defining the explicit sum, division, and other operations involve.

Banerjee, A. V. (1992). A simple model of herd behavior. The quarterly journal of economics, 107(3), 797-817.
John, T. J., & Samuel, R. (2000). Herd immunity and herd effect: new insights and definitions. European journal of epidemiology, 16(7), 601-606.
Moldovan, S. (2010). Investors Psychology And The Herd Effect On The Financial Markets. Revista tinerilor economişti, (15 spec), 21-26.
Simon, H. A. (1954). Bandwagon and underdog effects and the possibility of election predictions. Public Opinion Quarterly, 18(3), 245-253.
