# Supplementary material for: Crowd control: Reducing individual estimation bias by sharing biased social information
Source: PLoS Comput Biol. 2021 Nov 29;17(11):e1009590. doi: 10.1371/journal.pcbi.1009590 (PMC8659305; doi:10.1371/journal.pcbi.1009590)
Supplement: Attachment — Submitted filename: Response-to-Reviewers.pdf [file pcbi.1009590.s004.pdf]

Answer to Reviewers

Dear Editor,

We are grateful for the opportunity to resubmit our manuscript, and thank you and the reviewers for providing such thoughtful and detailed comments and suggestions, which greatly helped improving our manuscript. Based on the comments, we completely reworked our manuscript. Most importantly, we (i) provided more details on our information sharing methods, thereby clearly indicating its boundary conditions and clarifying how it uses prior knowledge about the underestimation bias, (ii) provided more evidence for the presence of the underestimation bias, while again acknowledging that in other domains there may be no bias or an overestimation bias, (iii) clarified or changed key concepts such as “biases”, “judgments” or “exchanges”, and (iv) entirely reshaped the section where we define collective and individual accuracy, as well as the end of the discussion. Moreover, we added, as suggested, several new figures to the Supplementary Information, as well as a few sentences about Condorcet’s Jury Theorem in the discussion, as suggested by the Editor. At the end of the manuscript we share a link to access the data on figshare. The data will be publicly available upon acceptance.

Please find below a detailed list of our responses to all reviewers’ comments. We took the liberty to relabel all reviewers’ comments, in order to ease the referring to other comments. Line numbers and references refer to the new version of the manuscript.

We believe this revision has substantially strengthened our manuscript and we hope it will be considered acceptable for publication in *PLoS Computational Biology*.

Yours sincerely,

Bertrand Jayles and Ralf Kurvers

Reviewer #1

Jayles and Kurvers investigate how information exchange between group members affects the accuracy of both individual and collective estimations. In particular, they investigate the effect of provision of varying quantities of others’ estimates, and propose a new framework for information exchange based on the shifted-median of previous estimates that improves collective and individual accuracy compared to simply providing estimates from other individuals at random. An agent-based model is used to explore the mechanisms behind this improvement and the dynamics of individual estimate changes.

The central finding in this manuscript is that by ‘leveraging prior knowledge’ about a common underestimation bias, information exchange can be structured to improve collective and individual accuracy. Essentially what this means is that, since we know that people will typically underestimate quantities by a certain proportion, and that they will move towards other estimations they are given, their accuracy can be improved by providing an estimate that is (statistically speaking) likely to be a slight overestimation of the truth.

The study appears well-conducted, and the combination of empirical and modelling work is well constructed. However, as it stands, I do not find the central finding sufficiently significant for publication in PLoS Computational Biology. I would justify this as follows:

1. Although the authors contend that their shifted-median method does not rely on recourse to the truth, this is only true in the sense that the answer to one specific question is unknown. It relies on strong statistical regularities in the relation between individual estimates and the truth. This is what is meant by ‘leveraging prior knowledge about this bias’ in the abstract.

We agree with this observation and also other reviewers pointed out that “leveraging prior knowledge” was imprecise. We have now clarified that our method does not require prior knowledge about the

correct answer to the question at hand, but it requires prior knowledge about the task domain to which the question belongs. The importance of domain knowledge was clarified throughout the text.

2. Where these regularities apply, it would be more straightforward to simply adjust all individual estimates or the collective estimate (however obtained) directly, rather than by the contrived mechanism of providing individuals with estimates from specially chosen other individuals. There is no sense here that the selective exchange of social information could be generated endogenously from within the group, but instead it is imposed by an external agent. This same external agent could instead manipulate either individual or collective estimates directly.

To approximate the correct answer better, it is indeed possible to apply a transformation to the initial estimates, as was done for instance in reference [24]. We would, however, like to point out that our goal in this article was not to find the best way to aggregate independent judgments (such as in Wisdom of Crowds approaches) but rather to study how to boost the accuracy of individuals, and to better understand the processes underlying human social information use.

3. Where these regularities do not apply there is no reason to think that this method would give improved estimations (and could even make them worse). The authors' own introduction reveals that human estimation and decision making is prone to many contradictory biases (e.g. pessimism and optimism, L43-44). It is unlikely that one could reliably know in advance whether the specific context lends itself to underestimation (though if one could, see point 2 above).

We were indeed not clear enough in our definition of biases, as well as about the domain of applicability of the underestimation bias, as also pointed out by other reviewers. This has now been carefully clarified. More details are given in comments #4 and #9 below.

To make a stronger case for the relevance of these results, this manuscript therefore needs a convincing motivation for:

4. The underestimation bias being widespread, important and reliably present, or identifiable in advance (so that the method works and it is known that it will work)

We agree with Reviewer #1 that a pre-requisite for our method to work is to know the domain to which a question belongs. In the domain we study (large quantities in estimation tasks), underestimation is a robust finding as we show in our manuscript based on previous data (Fig. 1) as well as multiple references. We have now clarified better the boundaries of the domain we study and added more references to emphasize the consistency of this bias in this domain (lines 62-65).

However, and as pointed out by other reviewers, we agree that in different domains people, on average, either underestimate, correctly estimate, or overestimate depending on the characteristics of the domain, underlining the importance of domain knowledge. We would like to emphasize that our method can also be used when there is overestimation in a domain, by sharing estimates below the median. As such, our method is not limited to underestimation. We now discuss the potential extension of our method to other domains in the discussion (lines 364-367).

5.a. Why it is important to affect individuals estimations via the provision of selectively chosen estimations from others,

Our goal in this study is to develop strategies to improve people judgments, *in social information sharing contexts*. Such contexts are widespread in modern digital societies, where the pervasive use of social networks and recommender systems has changed how people interact and make decisions, as discussed in the introduction. Our social information sharing paradigm mimics such contexts and is therefore highly relevant to our purpose. To make it clearer, we added "in social information sharing contexts" at the end of the last sentence of the second paragraph (line 56), and in the discussion (lines 361-362).

5.b. rather than either directly manipulating the original estimates

As already mentioned in comment #2, our goal is to boost the accuracy of individuals, and to better understand the processes underlying human social information use.

5.c. or simply providing alternative information to estimators (e.g. "experience suggests you are likely to be underestimating, consider raising your estimate").

Providing alternative information to estimators to boost individuals' accuracies is an interesting suggestion and could potentially work. However, it could also be problematic, as prescriptive information may be misinterpreted, arouse suspicion, or more generally alter participants' behavior. The effectiveness of such methods compared to our method is an open research question and beyond the scope of our work.

Minor points:

6. It would be interesting to compare the accuracy of the mean as both an individual and collective measure as well as the median. The authors contend that the median of group estimates are more reliable (L132): they may certainly be more robust/less variable for small samples, but it would be useful to see if the stronger effect on the mean of the rare large estimates would counteract the underestimation bias.

As suggested, we plotted collective and individual improvements using either the mean or median:

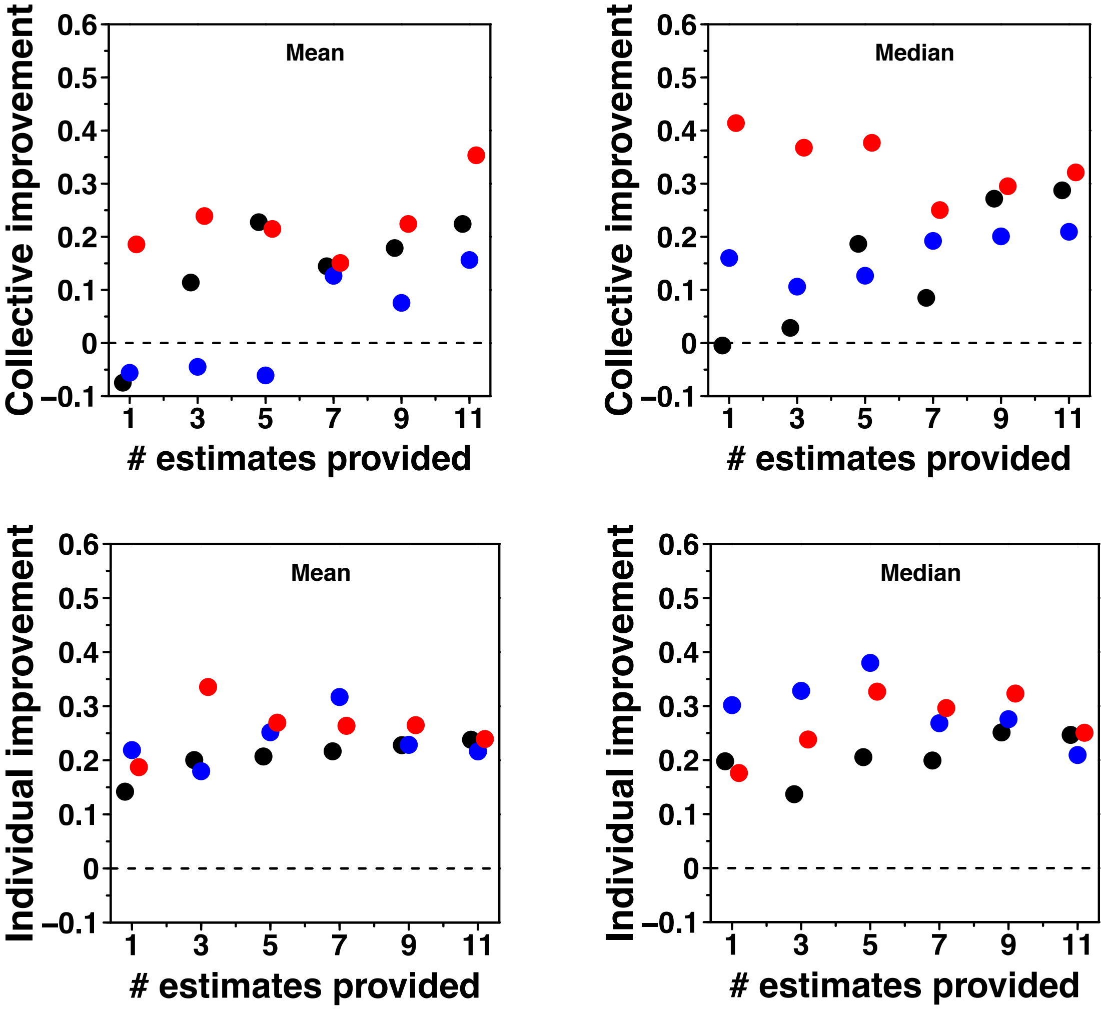

The median outperforms the mean in collective improvement, with little differences observed in individual improvement. Rare large estimates thus do not seem to systematically counteract the underestimation bias. Given the higher performance of the median, we decided to keep this measure in the main text.

It would also be good to see if the method used here works for estimation of quantities that are not strictly positive.

It would indeed be very interesting to investigate whether our method works for negative quantities, or quantities lower than 1 (i.e., negative in log). However, we are not aware of any such studies. We added it as a motivation for future research in the discussion (lines 366-367).

7. The modelling work in the manuscript (largely explained in SI) is detailed and shows a good progression of models to explain features of the empirical data. A further suggestion would be to consider how each effect used in the agent-based models can be justified in terms of rational or adaptive behaviour. At the very least, there is an established statistical literature on information integration that could inform the dependence on dispersion of social information.

We agree that this is an interesting suggestion and discuss this now more extensively in the discussion. The similarity effect is indeed adaptive, as agreement generally is a cue for accuracy. We discuss it with references ([54, 55]) in the discussion (lines 325-327). Also, the herding effect is likely to be adaptive as in most real-life contexts, personal information is limited and often insufficient, such that relying on social information, at least sometimes and partly, is an efficient strategy to make better decisions. We now discuss this on lines 292-295. The asymmetry effect in our experiments was also adaptive as it generally shifted people's estimates toward the truth. We improved the discussion around it, following also suggestions made by Reviewer #3 (lines 308-321).

8. Participants were motivated to be accurate by relatively small financial reward differences based on categories of accuracy. It would be interesting to consider and discuss whether the specific structure of this reward influences the types of estimate received. For example, what if the occasional very large error had a greater or lesser effect on the final reward? Or rewards based on accuracy relative to other participants rather than solely individual accuracy?

We agree that it is interesting to study non-linear reward structures, or reward structures that are relative to an individual's performance in the group. We chose a reward structure that we thought to be the most appropriate to elicit honest answers from participants, i.e., an absolute reward structure where all values are evaluated on equal footing. A relative reward structure seems somewhat risky as this might elicit untruthful reporting of social information. Although these are interesting suggestions, we consider these to be beyond the scope of our manuscript.

9. L44: How can biases be individually rational? I think this requires an example considering that it is unintuitive

Originally, we were not clear enough in our definition of the term "biases", which led to confusing statements. We now provide a clear definition: deviations from assumed rationality in judgment, and removed the examples (optimism, pessimism) that do not necessarily adhere to this definition, thereby clarifying the first few sentences of the introduction (lines 41-43).

10. I felt the manuscript could be clearer in places by using more straightforward English. For example, replacing 'leveraging' with 'using' and 'potentialities' with 'potential'. This is not to criticise the general standard of English, which is otherwise high.

As suggested, we replaced the instances of such words by more common English words.

****
****

<u>Reviewer #2</u>

I will state first that I am not in the area of social choice, and my expertise is more in sequential design of experiments and Bayesian Optimization, which are techniques that could have an important role in the work presented by the authors.

The paper looks into the problem of underestimation bias in a setting with a group of people is asked to provide an estimate and subsequently is provided with several estimations from the remainder of the group (a subset). The authors use three mechanism to select how to exchange information: a random mechanism, a median driven mechanism, and a shifted median exchange, which considers the overestimation bias and compensates for it using a

factor γ. The authors proceed optimizing this factor and identify three modeling factors to be considered to study the impact of the amount of exchanged information onto the individual accuracy and collective accuracies. These aspects are *herd, asymmetry, and similarity*.

The paper was an incredibly interesting read, I believe the authors make a good case on the contribution of their work compared with the available literature, which appear to be herd, asymmetry and similarity. I can appreciate the modeling value in this. However, there are some aspects which I believe the authors should clarify to make the paper contribution clearer and, possibly, stronger.

1. What is the effect of the specific population being chosen? Are there aspects of the model that can indicate different outcomes in terms of the accuracy, based on the population? Can we have populations that are over and underestimating?

The participants in our study were German undergraduate students only. However, studies conducted in different countries point to similar levels of underestimation bias and social information use. For instance, in reference [20], the authors compared France and Japan, as well as men and women, and participants were from different ages and social background. In these studies, similar levels of underestimation bias and social information use were observed. However, we agree that future studies comparing how different (sub-)populations are biased and use social information is an important step, and we added it as a motivation for future research in the discussion (lines 373-379).

That being said, *once the bias and level of social information use in a population is known, our method would apply and work the same*. Adapting the model would be straightforward, by changing the values of the initial bias and the effects' parameters accordingly.

2. It is unclear whether the theory being developed in this paper may apply to non quantitative cases. The authors refer in multiple places to recommendation systems, but more details should be provided on how the very idea of underestimation bias that the authors use to define the coefficient γ, can be extended to cases where no quantitative measures are available.

This is a very interesting comment, that helped us reshape the end of our discussion. Also inspired by comments from other reviewers, we have now clarified the domain of applicability of the underestimation bias (lines 61-65), and hence of our method. In the discussion, we now discuss possible extensions of our method to other domains related to estimations (lines 365-367), as well as to different domains where a true answer does not necessarily exist, such as opinions dynamics (lines 380-387).

In the following some more detailed comments are provided.

Detailed comments:

3. On page 4-5, the authors discuss the experiment design and the over-compensation mechanism. There is an underlying message in this part that starting with bad estimated we can improve accuracy by mixing the bad estimates in an effective way. This concept is the heart and soul of *boosting* in statistics and machine learning. Do the authors think there is somewhat some relevance of the boosting literature that could lead to a statistical justification for the choice of the size of the set and the weight to give to each of the selected information?

We do not really "improve accuracy by mixing bad estimates in an effective way", but rather by sharing relevant social information. Although we are not very familiar with the concept of boosting in the machine learning literature, it seems to be more closely related to Wisdom of Crowds approaches, which aim at approximating the truth from an initial set of estimates, without improving the estimators' accuracy. Our aim is specifically to improve estimators' accuracy. We therefore prefer not to discuss the boosting literature in the text, as it may confuse the reader regarding our methods and goals. In psychology the term "boosting" also has a very different meaning which also could cause confusion.

4. Always related to the experiment, the authors select a homogeneous population age-wise. What it the impact of this choice? When we talk about news and social media the population will be way more heterogenous. Could this represent an issue?

As discussed above in comment #1, different sub-populations may indeed yield different results. However, this is not an issue, in the sense that our method uses prior knowledge about biases to dampen their effects. Provided a bias is known (in a population and a domain), the method can be adapted straightforwardly.

5. The authors claim that γ can be derived without knowledge of the true value of the measure. However, it was not clear to me how γ = 0.9 was derived. It seems to me that γ = 0.9 requires to be set the knowledge of the true value. Do the authors mean that since they optimize for γ they actually do not need to assume a value for the truth?

Our original wording was a bit unclear and has most likely added to this confusion, as also pointed out by several other reviewers. We have now carefully addressed this comment. In summary:

- our method requires domain knowledge (from prior studies) about the tasks (here large quantities in estimation tasks);
- in the present case, this knowledge is that large quantities tend to be underestimated, and that the regression coefficient of this underestimation is γ = 0.9 (Fig. 1);
- this prior knowledge can then be used in *new tasks belonging to the same domain*, for which the true value does not need to be known. Subjects provide their personal estimates, and from these estimates and γ, the shifted-median value is computed and serves as a reference for the selection of the social information.

This has been now thoroughly revised and clarified throughout the text.

6.a. page 6, line 161-162. Could the observed effects be associated with the specific choice of the source be shared?

Because the effects were observed across all three treatments (i.e. across three different specific choices of sources to be shared), we can reasonably assume that the effects are not associated with a specific choice, but reflect key human cognitive mechanisms.

6.b. Is there a possibility to identify "better evaluators" that have higher individual accuracy that the others can tend to? If not would that be possible to bias towards?

Our method actually identifies better evaluators. Indeed, the method selects the estimates that are closest to a value (the shifted-median value) that counters the underestimation bias, and is thus expected to be close to the truth. Our method thus "biases" second estimates towards answers from better evaluators.

6.c. This is very common in recommendation systems or crowd sourced search engines, where people that post responses are evaluated by the algorithm and if they are consistently right, they get "higher weight". Can this mechanism be considered?

Other methods to identify good estimators could indeed be imagined, and have been suggested in past research. For instance, in reference [34] the authors find that individuals who resist social influence are usually more accurate than others, and reference [54] shows that similarity in judgment often correlates with accuracy. And, as suggested by Reviewer #2, evaluation over multiple items can also be used to detect accuracy. However, to what extent such methods, or any other one that one could think of, compares to ours remains an open question.

6.d. Or here we can specifically solve underestimation bias?

As mentioned in comments #2 and #4 above, the same method can be used to solve other biases.

7. page 6, line 161. The authors say that the shifted median collective accuracy is basically concave in the amount of data being exchanged. However, do the authors mean that the relative improvement of shifted against the rest is decreasing with the number of data being shared?

Indeed, the relative advantage of the Shifted-Median treatment compared to both others diminishes when increasing the number of estimates shared. We now mention this in lines 181-182.

8. Shouldn't the y-axis in Figure 2 be the relative improvement and not the absolute improvement?

Reviewer #2 is right, the improvement is relative. We accordingly added "Relative" to the beginning of the caption's title (also in Fig. S2). However, due to a lack of space on the legend axes, we only use "collective/individual improvement" there. We now explicitly state in the main text, lines 167-168, that we refer to "collective/individual improvement" and to "relative improvement in collective/individual accuracy" indifferently.

9. Page 10, line 232. So do the authors mean that the herding effect is a by-product of the model? Can the authors expand this explanation?

The original formulation was indeed confusing. The herding effect is **not** a by-product of the model, as we directly observe it in the data (Fig.3). What we meant is that this effect does not need an explicit expression in the equation of the model, as the "average weight $\langle S \rangle$ given to the social information being strictly between 0 and 1" depends on the parameter values in the model, and especially μ_g (μ_g = 0.5), the mean of the Gaussian part of the sensitivity to social influence S. For instance, if μ_g was negative, then $\langle S \rangle = P_g \mu_g$ would also be negative, and there would be no herding effect. This is now clarified in the main text (lines 261-264) and in the Supplementary information (lines 188-191).

10. Optimal γ. The fact that the authors are finding a $\gamma \neq 0.9$ and could be substantially different, may be a disprove of the logarithmic perception model assumption?

The model predicts that a value of γ which aims to overestimate the truth—rather than approximate it— would yield even better results. The idea is that since not all individuals follow the social information fully, trying to pull their estimates towards the truth improves accuracy (that is what we did), but pulling their estimates even further, towards an overestimation of the truth, would improve accuracy even more. This is a model prediction that, however, does not disprove the logarithmic perception assumption, since the model is based on it.

11. Figure 5.b. I find quite interesting that the size of the group and the value of τ^* (i.e., the best amount of information to share for a specific group size) is very different between collective and individual accuracy. Does this imply that these two are in trade off? The authors seem to suggest at the beginning of the paper that this is not the case, but could they dedicate some explanation to this fact?

The two measures of accuracy are not *a priori* in trade off but, since they are two different measures, they might be if they yield different conclusions. As noted by Reviewer #2, the predicted optimal value of tau is different for collective and individual improvements, such that the question arises which optimal value to choose. Since individual improvement does not vary much with tau (Fig.2b) or gamma (Fig.5a) compared to collective improvement, the value of tau that maximizes collective improvement should be favored, if the goal is to optimize both collective and individual improvements. This is now discussed lines 282-286. Note that the same analysis and conclusion holds for the dependence of individual and collective accuracy on gamma, and is discussed lines 271-273.

12. Discussion. A possible limitation is that the very concept of underestimation bias appears to be inherently connected to having quantitative estimates, how can this concept for example be extended to cases such as ranking and recommendation systems? I have no problem with the fact that the authors may be looking into a very specific type of bias, but then I think it would be fair to better qualify the statements in the conclusions about the future applications.

This is certainly true. As explained in comment #2 above, we have now clarified the boundaries of our method, and discuss more carefully the potential extensions of our method in the two last paragraphs of the discussion.

****
****

Reviewer #3

This is an interesting paper reporting a model and an empirical study of collective judgment. The study seeks to understand the effects of information sharing in groups and in particular the effects of the amount of information shared and the selection process of which pieces of information are shares. A particular point of interest from the authors' perspective is how well can a particular process (which they label "shifted median") designed to counter natural individual judgment "biases" improve the quality of the judgments.

I like the topic and the approach. I think the experiment is well designed and, for the most part, it is well analyzed and clearly reported, but, in my view, this version is not ready for publication. Many of my reservations are related to the writing and presentation style which is imprecise and involves some over generalizations and is, occasionally, sloppy. I will list many of these instances in the order I spotted them in the manuscript, and not necessarily in terms of their importance or severity.

1. There is a basic distinction in the literature between judgments and decisions. *Decisions* involve choices or valuations (usually of competing options) and involve consequences of these actions (often, but not always, monetary). For example – should I invest in A or B? Should I take medication X or Y? How much should I pay for this car, apartment, dress, etc.? *Judgements* are, as the name indicates subjective estimates of quantities, frequencies, probabilities, etc. that carry no such consequences. This paper is all about *judgments and judgment biases*, but the authors often refer incorrectly to *decisions.* This should be corrected throughout the ms.

Reviewer #3 is correct. We replaced "decisions" by "judgments" everywhere, and changed "collective decisions" with "collective judgments and decisions" where appropriate.

2. The authors refer to a lot of *biases* without ever properly defining what they mean. In some sense, without proper contextualization, any empirical regularity can be labeled a bias. In the classical work by Tversky and Kahneman biases are defined with reference to a normative model (probability theory) that dictates how judges should act in various circumstances (and even this approach is subject to criticism as in Costello and Watts, 2014), but in many of the cases listed in the paper, I am unsure why things are labeled biases. Many of them (e.g., Optimism, Pessimism) can be explained by other simpler accounts that are totally "unbiased". This needs to be clearly explained.

At the end of the first sentence, we now provide a definition of the term biases, supported by a reference: deviations from assumed rationality in judgment. We agree that we originally were not very clear in our definition of this term. We removed the examples (optimism, pessimism) that do not necessarily adhere to this definition. The remaining examples given in the introduction (equality bias and ingroup bias) are referenced and follow our given definition.

3. Is there a human tendency to underestimate quantities? I don't think so! I think that it is fair to say that that *human judgment is regressive and people tend to over (under) estimate low (high) quantities* (see, for example reference [29] in the paper). This paper focuses primarily on "large" quantities (see the list in appendix), but fails to state this explicitly (exception line 129) and systematically, and creates the false impression that this is a more general pattern. This needs to be corrected throughout.

We agree with Reviewer #3 that we did not delimitate the domain of applicability enough. We now clarified this throughout the text (see in particular lines 61-65), and changed "underestimate quantities" to "underestimate *large* quantities" throughout the text. We give more details in our answer to Reviewer #1 (comment #4), who made a similar remark.

4. There are multiple references to "human tendencies" (see for example line 22 – 23 in the Abstract). I think every one of these (over?) generalizations should be accompanied by some references to back it up.

By using the word "tendency", our intention was actually to avoid (over)generalization. Tendency here means that a phenomenon is dominant in a population, but not absolute. Reviewer #3's comment suggests that our use of this term was unreferenced. However, every instance was carefully

referenced, except maybe one that we did not judge necessary (ingroup bias). *We added a reference to it in the new version*. Here are more detailed explanations:

- In the abstract and author summary, we did not, as is the custom, add references.
- In the introduction, line 43, the term “tendency” is backed up by a reference.
- In the introduction, line 52, *we added a reference ([14])* when discussing the ingroup bias.
- The first mention of the underestimation bias (“tendency to underestimate quantities”), line 62, was backed up by 5 references (we added three more in the new version [20-27]).
- The herding effect (tendency to partially follow the social information), line 207, is something we show in the article, and is therefore not backed up by references. However, contextual references were added in the original text where necessary.
- In the discussion, the tendency to underuse social information, line 329, is backed up by 3 references.

5. I am puzzled by the use of the term “exchange of information”. Every definition I am aware of, stresses the bi–directionality of any exchange, but in this context people are only receiving information from others and they can revise / adjust / refine their judgements in light of this new information, but they don’t offer anything in return, so there is no “exchange”. It is true, that every subject’s judgments are presented to the others in the group, but it is not clear to me that they know this and that there is any reciprocal thinking involved here. So, I would replace the term exchange with one that describes more accurately the setup.

We agree with Reviewer #3 that the most common usage of “information exchange” indeed implies bi-directionality. We, therefore, replaced in the new version the word “exchange” by expressions such as “estimates selected and presented to the participants”, which accurately describe the setup. In some instances, we used the verb “share” (inspired by Reviewer #3), which does not imply bidirectionality.

6. I think that the three presentation formats are not presented clearly enough, and I think it is worth explaining, what took me a while to recognize, namely that the Median differs from Random simply because it *eliminates extreme values* (essentially, trimming) and presents only the X/11 (X = 1,3,..11) central values of the distribution, and that Shifted Median presents *the same values*, but after a systematic shift.

Following Reviewer #3’s suggestion we added, in the description of these two treatments in the introduction, an additional sentence to give a better intuition of what these selection methods actually do (lines 95-96 and 107-110).

7. The statement on lines 96 – 97 is mathematically wrong (or, maybe not clearly stated): It easy to show many cases where the expected Random choice is closer to the truth than the Median. *Example:* Truth = 10; Assume a group of 6 people such that the 5 potential estimates are 1,2,3,4 and 9 (distances from truth = 9,8,7,6 and 1, respectively). If we choose k = 3 the median selects the estimates 2, 3 and 4 with a mean (and median) distance of 7 from Truth. But, if you consider all 10 (equally likely) different ways to choose 3 of the 5 you get Mean(10 Medians) = 7 and Mean(10 Means) = 6.2! And, if you choose k = 1, the median selects 3 with a mean (and median) distance of 7 from Truth. Under a random choice the mean distance to the truth is, again 6.2! Please clarify / correct.

This sentence was not meant as an absolute statement, but rather as an empirical expectation following the first sentence of the paragraph: “in estimation tasks, median estimates are often closer to the true value than randomly selected estimates (Wisdom of Crowds)”. We agree that our statement was ambiguous, and we modified it by emphasizing that the selected estimates are *expected* to be more accurate on average than in the Random treatment (lines 96-97).

8. In forecasting there is a small literature on *re-calibrating probabilities in aggregation* (see papers by Baron et al and Turner et al). The shifted median is another, simpler, instance of re-calibration with a twist. In forecasting the transformation is done mechanically and externally after the estimation process. Here the judges are exposed to the re-calibrated judgments of their partners. This brings up an intriguing question. If one was to take the estimates from the Median condition and apply the same shifting transformation, how would these recalibrated aggregates compare to those obtained in the Shifted Median condition? Clearly it is easier to recalibrate things statistically / mechanically, but is it also better?

This is indeed an interesting question. Please find below a graph where we show the collective accuracy (not the improvement thereof) of (i) the second estimates in the Shifted-Median treatment (red) and (ii) the first estimates in the Median treatment (blue), recalibrated with the same shifting transformation (i.e., all log estimates were divided by 0.9). Note that lower values indicate a higher collective accuracy. The recalibrated first estimates are actually more accurate than the second estimates in the Shifted-Median treatment.

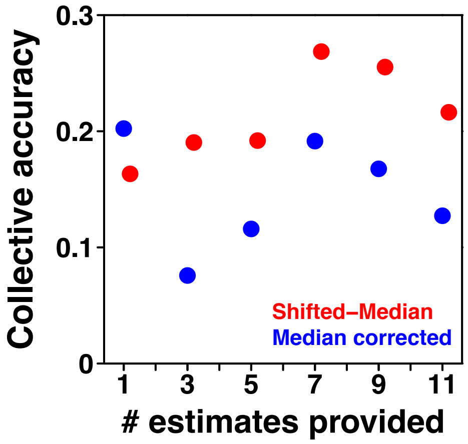

Although this is an interesting finding, this correction does not help participants improve their accuracy, and is therefore more in line with Wisdom of Crowds approaches, which purpose is to find the best way to aggregate initial estimates in order to best approximate the truth (note that a similar correction was suggested in reference [24]). Our method seeks to improve individuals' accuracy. Including this graph might therefore complicate the story line in our already relatively technical manuscript, hence we decided to not include it. We, however, now briefly discuss this in the section "Compensating the underestimation bias" (lines 146-149).

9. I did not fully understand the difference between the collective and individual accuracy measure and I was frustrated by the insufficient and inadequate discussion. I assume that the authors calculate an individual measure for each of the 216 people based on their 36 judgments and that they calculate the collective accuracy for each of the 18 groups based on the group's (12 members X 36 items =) 432 judgments. That is the way I would have done this but I am not sure this is what was done. I would like to see a better and clearer description.

Yes, individual accuracy is a measure of the distance of an individual's estimates from the truth, and collective accuracy is a measure of how far the central tendency of the estimates of the group is from the truth. We acknowledge that this part was not clear enough. We completely revised the beginning of the Results section, in which we introduce individual and collective accuracy, and we believe it is now much easier to understand (lines 156-166).

10. Related: On page 6 in the results section, you write "individual accuracy measures how close individual deviations from the truth are to 0 on average," while technically your individual accuracy measure is an individual's median accuracy across questions (correct?), not the mean.

See above comment; we have completely rewritten this section and believe it is substantially clearer. Individual accuracy is, for a given group in a given condition, the median absolute deviation from the truth of all individuals and all questions. Note that an average expresses the central/typical value in a set of data, such that both mean and median can be called averages.

11. The items (listed in Appendix) are clearly of two types: The majority is based on general knowledge of actual facts such as "What is the population of X?" and a minority ask for a perceptual impression / estimation (e.g., "How many marbles are in the jar"?). It would be nice to present some evidence that the degree of underestimation is similar in the two classes (for example, use different colors for the two in Figure 1) and that the proposed method works equally for both.

Below we plot the relationship between the truth and level of underestimation separately for general knowledge questions (green dots) and numerosity questions (orange dots). The slopes of the regression lines are 0.9 and 0.93 respectively, suggesting that the relationship between the true value and the degree of underestimation is indeed similar in both classes. We added this figure to the Supplementary Information and refer to it in the section "Compensating the underestimation bias" (lines 152-154).

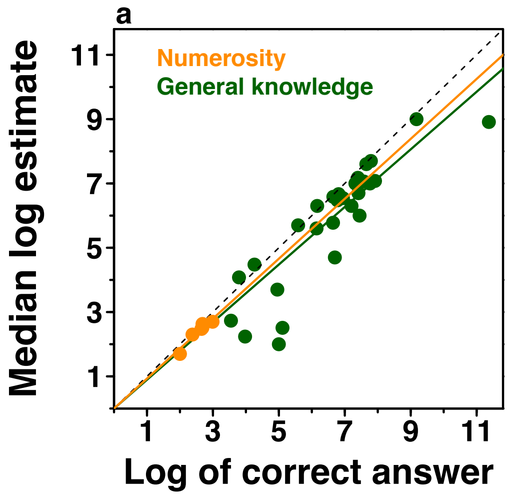

12. Line 166: when k = (n-1) = 11 one expects identical responses under various condition only in the absence of overweighting of one's own original judgment (egocentric weighting), which is often seen in the literature (e.g. Yaniv & Kleinberg, 2000) and clearly in the present data (note that most values of S < 0.5).

The three treatments are indeed expected to be rigorously equivalent when k = 11 (as written in lines 186-188), especially since we used a within subjects design. Since subjects were unaware of the treatments they were undergoing, there is no reason to expect any difference when k = 11, independent of egocentric weighting. This is confirmed by our observations.

13. To make sense of the asymmetry effect and Figure 3, we need to know what is the distribution of cases where the weight of the social information is <, approximately = or > than one's own.

We agree that this information is interesting. We added this as a supplementary table (Table S1), and comment on it in the asymmetry effect section of the main text (lines 220-225). In the Random treatment, D<0 and D>0 are equally likely, in the median treatment, D<0 is more frequent than D>0, and in the shifted-median treatment, D>0 is more frequent than D<0. Yet, in all three treatments people follow the social information more when D>0 than when D<0, so the asymmetry effect itself (i.e., its presence) seems largely independent of the distribution of cases. However, the *consequences* of the asymmetry effect in terms of improvement after social information sharing, are indeed expected to be linked to the frequency of how often D<0 and D>0 occur (line 223).

14. Related: Could a possible explanation for the asymmetric effect be that people, in fact, have some intuition that they tend to underestimate large quantities? Seeing others provide larger estimates than their initial belief may be a sort of cognitive permission to be more liberal with their beliefs about large quantities. While seeing smaller estimates may be seen as typical and expected.

We wholeheartedly agree with this comment, and we already provided this explanation, albeit phrased slightly differently, in the discussion of the manuscript. We rephrased the same idea in words closer to Reviewer #3's suggestion (lines 317-319).

15. Consider the improvements to collective accuracy as predicted by the model in the dashed lines of Figure 2a. Why does the model predict that the random selection method will improve collective accuracy more than the median selection method?

The predicted lesser collective improvement in the Median treatment, as compared to the Random treatment, is a direct consequence of the underestimation bias. In the Median treatment, subjects receive social information that is close to the center of the distribution of estimates. But, *because of the underestimation bias*, the answers that are above the median are on average closer to the truth than those which are below the median. By following the same biased information, the *relative* decay in accuracy for the answers above the median is stronger on average than the relative improvement in accuracy for the answers below the median.

16. This is especially puzzling, since this does not seem to reflect the actual differences between these groups, where it appears the random selection method was more linearly related to the number of estimates exchanged; while the median selection method was flatter across the number of estimates exchanged.

We agree that Fig. 2a gives the visual impression that collective improvement in the random treatment increases more linearly than in the median treatment, mostly because the blue point at tau = 1 is way higher than predicted by the model (and expected by us). Apart from this single point, both treatments increase, as predicted, linearly and it is likely that this single point deviated too much from its expected value due to noise, as often happens with limited samples. Though we tested 216 participants over 36 questions, our sample size per unique treatment combination is still limited. So, we believe we need to treat each single point with caution, but can have much more confidence in the general patterns.

17. A possible intuition for this result might have to do with how the distance effect parameter was treated. It appears that the relationship between distance and belief updates was treated as linear and increasing, but this is not necessarily the universally observed expectation based on the social persuasion literature. For example, Whittaker (1963) found a curvilinear relationship between distance and belief updates. Other studies have found similar results (Fink, Kaplowitz, & Bauer, 1983; Laroche, 1977; Yaniv & Milyavsky, 2007) and Allahverdyan & Galstyan (2014) proposed a formal model incorporating this effect.

We would like to emphasize that the relationship is linear, *once estimates are log-transformed*, meaning that our model is also non-linear on actual estimates, as in the references suggested by Reviewer #3. Importantly, the linear (in terms of log variables) and increasing dependence of S on the distance between personal estimate and social information in our model corroborates earlier findings using a similar paradigm [20, 45]. Fig.S8d supports this linearity rather than contradicts it, although the patterns are much noisier than in the references above mentioned.

18. Looking at figure S6d, it does look like a curvilinear parameter might fit the data better than the linear one proposed. When combined with your model's asymmetry effect, it seems possible that providing random estimates could lead to this somewhat odd result in Figure 2a. Random estimates are more likely to be extreme than median estimates (which are by definition the least extreme available). However, the asymmetry effect parameter means extreme estimates that are lower than the judges' initial estimates get discounted, while ones that are higher do not. This could induce a somewhat artificial correction for the underestimation bias that isn't present in the observed data. Treating the distance parameter as non-linear could potentially correct this and may be worth trying.

We tried Reviewer #3's suggestion, i.e. to define the distance effect as a curvilinear relationship (i.e., curvilinear on logarithms), but only observed marginal changes, and rather in the wrong direction (i.e., a lower model fit than the linear implementation). Moreover, the asymmetry effect is indeed not present (or too weak to be significant) in the data at tau=1, that is why we do not include it either in the model, the asymmetry effect term reading (tau - 1).

19. A somewhat related question is whether there were any effects of bracketing (see Herzog & Hertwig, 2009; Larrick & Soll, 2006; Soll & Mannes, 2011). The authors provide a formalization for how individuals incorporate the social information based on its geometric mean and standard deviation, but do not discuss whether people treat this information differently in cases where the estimates bracket their beliefs (whether their beliefs are within the bounds of the different estimates they receive) or do not. Normatively, in cases where people believe the estimates they receive bracket the truth, they should be more inclined to average and weight that advice fairly heavily; while in cases where the estimates they receive do not bracket the truth they should not (though this normative principle is not always observed in behavior). One could argue that estimates which bracket a judge's initial estimate could be

considered a bracket around their a priori belief about the truth, which would make such brackets qualitatively different from ones that do not. This could also have implications for comparing the random and median conditions. Especially when the number of estimates received is small, the diversity of random estimates may be more likely to bracket a judge's initial beliefs; while the more homogenous median estimates may be less likely to.

As Reviewer #3 points out, the bracketing of estimates around the personal estimate is much more likely to happen in the Random treatment than in the Median and Shifted-Median treatments. However, Fig.3 shows that subjects followed social information more in the Median and Shifted-Median treatments than in the Random exchange. This suggests that if an effect of bracketing exists, it is marginal and exceeded by the similarity effect. From our results it seems more likely that individuals take similarity of advice as a cue for quality of advice, independent of whether this brackets their own belief.

References

Allahverdyan, A. E., & Galstyan, A. (2014). Opinion dynamics with confirmation bias. PloS One, 9(7).
Baron, J., Mellers, B.A. Tetlock, P.E. Stone, E., Ungar, L.H. (2014) Two Reasons to Make Aggregated Probability Forecasts More Extreme. Decision Analysis 11(2):133-145.
Costello, F. & Watts, P. (2014). Surprisingly rational: Probability theory plus noise explains biases in judgment. Psychological Review, Vol 121(3), J 463-480
Fink, E. L., Kaplowitz, S. A., & Bauer, C. L. (1983). Positional discrepancy, psychological discrepancy, and attitude change: Experimental tests of some mathematical models. Communications Monographs, 50(4), 413–430.
Herzog, S. M., & Hertwig, R. (2009). The wisdom of many in one mind: Improving individual judgments with dialectical bootstrapping. Psychological Science, 20(2), 231–237.
Laroche, M. (1977). A model of attitude change in groups following a persuasive communication: An attempt at formalizing research findings. Behavioral Science, 22(4), 246–257.
Larrick, R. P., & Soll, J. B. (2006). Intuitions about combining opinions: Misappreciation of the averaging principle. Management Science, 52(1), 111–127.
Soll, J. B., & Mannes, A. E. (2011). Judgmental aggregation strategies depend on whether the self is involved. International Journal of Forecasting, 27(1), 81–102.
Turner, B.M., Steyvers, M., Merkle, E.C., Budescu, D.V., & Wallsten, T.S. (2014). Forecast aggregation via recalibration. Machine Learning, 95, 261-289.
Whittaker, J. O. (1963). Opinion change as a function of communication-attitude discrepancy. Psychological Reports, 13(3), 763–772.
Yaniv, I., & Milyavsky, M. (2007). Using advice from multiple sources to revise and improve judgments. Organizational Behavior and Human Decision Processes, 103(1), 104–120.
Yaniv. I. & Kleinberg. E. (2000). Advice Taking in Decision Making: Egocentric Discounting and Reputation Formation. Organizational Behavior and Human Decision Processes, 83(2), 260-281.

****
****

Reviewer #4

After reviewing the manuscript in detail and performing an independent search to verify some of the authors' claims regarding the novelty of their present work, it is my belief that the paper makes worthwhile contributions to the theory of (human) collective decision-making. However, the manuscript lacks key details in various parts, which make it difficult to contextualize and quantify the true impact of these contributions. Upon the considerations and suggested edits listed below, it is my opinion that the paper should undergo a major revision to address the considerations listed in the below paragraphs.

The key considerations are as follows:

1. Missing details about the estimation activities. The estimation experiments performed as part of this research are listed in the appendix, but there is little information about them within

the body of the paper. This lack of detail should be addressed because the principal message/applicability of the paper should be self-contained and, more importantly, because the underestimation bias is not relevant to every type of estimation task. Indeed, there are a number of cognitive overestimation biases—estimating the likelihood of certain events (e.g., see reference [27] of manuscript), travel times, phantom patterns in data, etc.—that would be exacerbated by shifting up the collective estimate. The authors are thus asked to provide the general characteristics of their estimation activities up front and from these to describe the types of estimation activities for which their approach is likely to be most relevant later in the paper. They should also mention other estimation tasks where it may not be advisable to apply their techniques and/or where a shift of the median in the opposite direction may be the preferred option. On a related note, the authors are asked to specify the equation for the median shift up front (the derivation can remain in the appendix) and to specify for what types of activities the shift is expected to be applicable.

We agree with Reviewer #4 that we were not precise enough when defining our tasks, and similar comments were made by other reviewers. We now describe more clearly the domain of applicability of our estimation tasks. For a more detailed response, please see our answer to Reviewer #1, comment #4.

As for the equation, it is specified in the Materials and Methods, which are placed after the introduction, and before the results. The equation is very simple and derived from the linear relationship between the median m of the log estimates and the log of the true value T (Fig. 1): $m \sim \gamma \log(T)$ (line 141). For any quantity that belongs to our specified domain (i.e., large quantities in estimation tasks) the expected (log of the) true value can be approximated by the shifted-median value $m' = m/\gamma \sim \log(T)$ (line 145). Moving the equation more upfront would mean putting it in the introduction. We think it is better to keep it in the Materials and Methods, where it is accompanied by the corresponding figure that supports it (Fig.1).

2. Novelty of introduced effects. The authors introduce different mechanisms underlying the integration of multiple pieces of social information. To measure the effectiveness of these mechanisms, the authors introduce *effects* which may be known by other names in other fields and/or which may clash with existing terminology. For instance, the introduced *herding effect* seems to be related to the well-known *bandwagon effect*, which is the tendency of individuals to follow what others are doing, especially when a lot of other people are doing it (Simon, 1954). Moreover, a *herd(ing) effect* exists in psychology (Moldovan, S. 2010), economics (Banerjee, 1992) and epidemiology (John and Samuel, 2000). The authors are asked evaluate the appropriateness of their effect names and to clarify any relevant similarities/differences with other existing similar concepts.

In the discussion, we now added several more references to studies that discuss similar effects, showing that the names that we have chosen are relevant and appropriate. In particular:

- The "herd behavior" described by Banerjee results from the human tendency to copy each other, i.e., our herding effect (ref [52]). See lines 302-304;
- An "asymmetric effect", similar to our "asymmetry effect", has been found in a paper suggested by Reviewer #5 (ref [24]). See lines 321-323;
- Reference [54] shows that decision "similarity" (in their own terms) correlates with accuracy.

3.a. Accuracy of estimates. There two issues with the way that the accuracy of estimates is presented that obfuscates what was achieved in the experiments. First, the magnitude of the ground truth *T* for the questions that were asked is widely varying (from 100 to billions). Hence, aggregating the errors over all the questions— which is what I believe is done to summarize the results, but please correct me if I'm misinterpreting this—would naturally lead to an overrepresentation of the errors from the questions with the largest magnitudes, even if the reported statistics are the logs of the aggregate statistics. Here I would suggest dividing the questions into groups based on magnitude ranges of *T* (or providing results of individual questions) and separating the results accordingly

To investigate this issue, we compare the improvements in collective and individual accuracy for the 18 questions with the smallest true values, and the 18 questions with the largest true values:

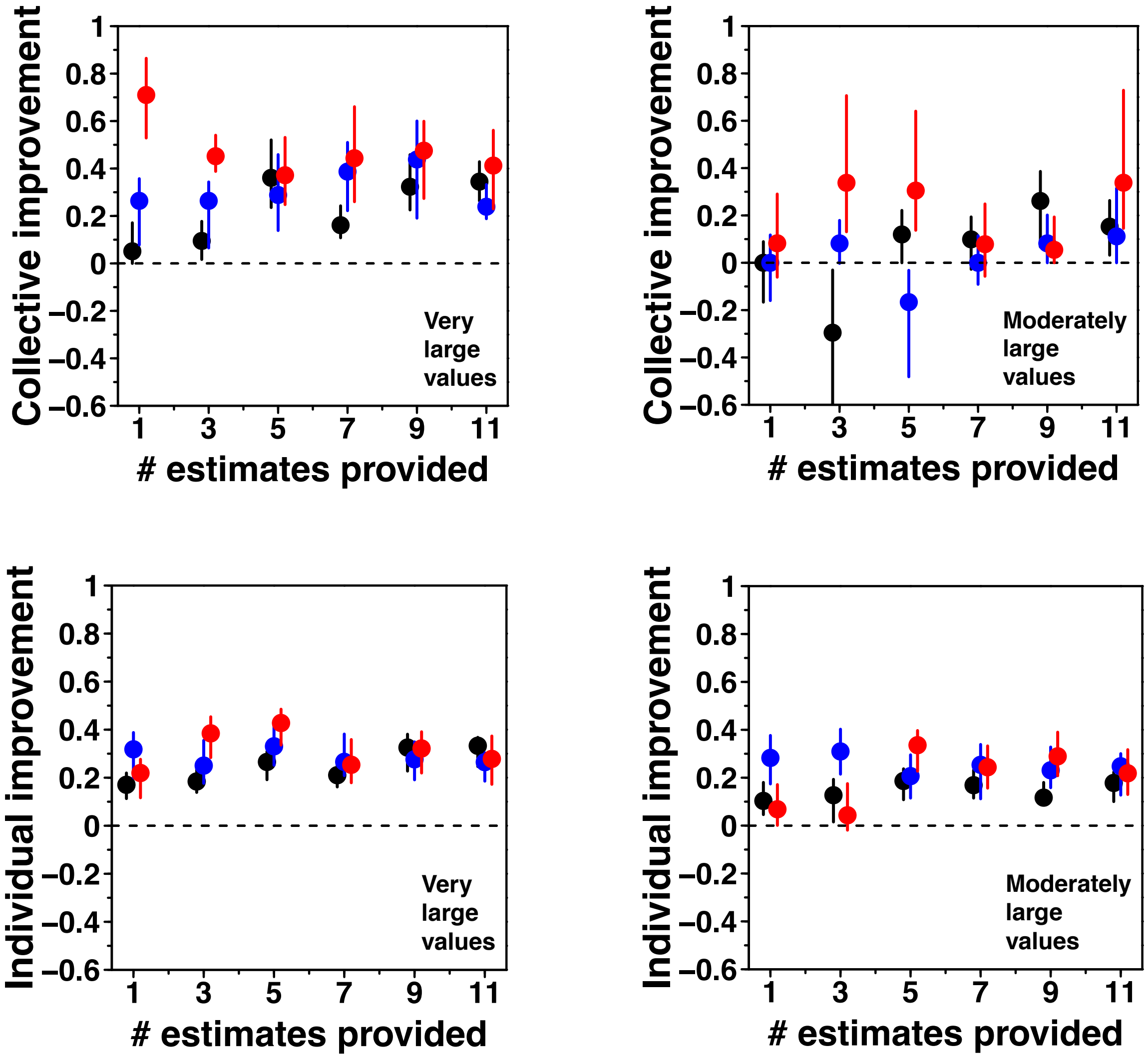

The collective and individual improvements are somewhat higher for questions with large true value, although the levels of underestimation are similar in both cases:

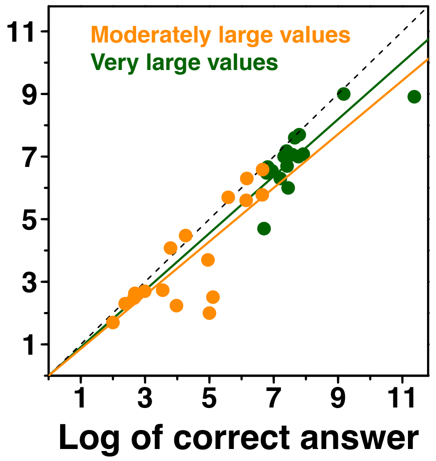

This suggests that the relationship between the median log estimate and the log of the true value may not be sufficient to fully characterize domains, and that other distributional properties could be used to refine our method further. We have included these graphs as Supplementary Figures and discuss potential extensions/refinements of our method in the discussion (lines 367-373).

3.b. and to report the absolute deviation of the collective and individual estimates to *T* in addition to the logarithms of these quantities.

Below we show collective and individual accuracy (and not the improvement thereof), defined as in the main text (i.e., using logs), as a function of the number of estimates provided, for the three different treatments. The closer the values to 0, the higher the accuracy, 0 denoting perfect accuracy.

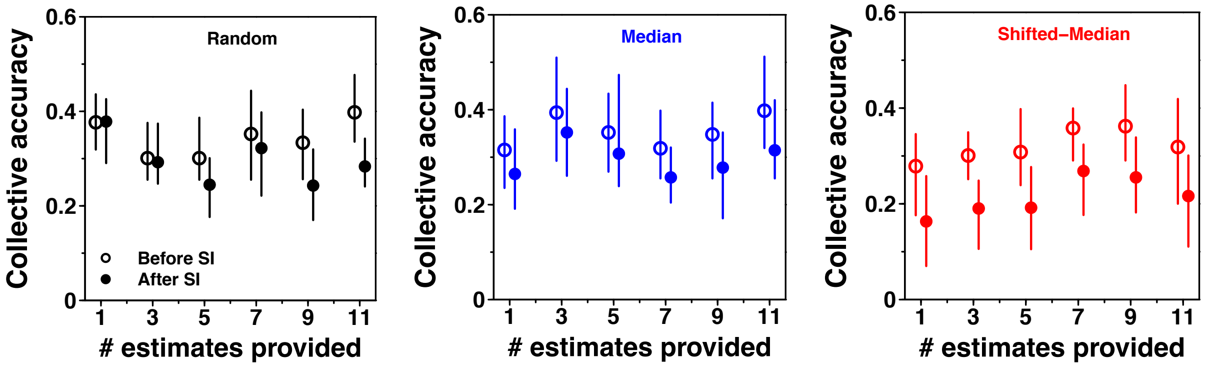

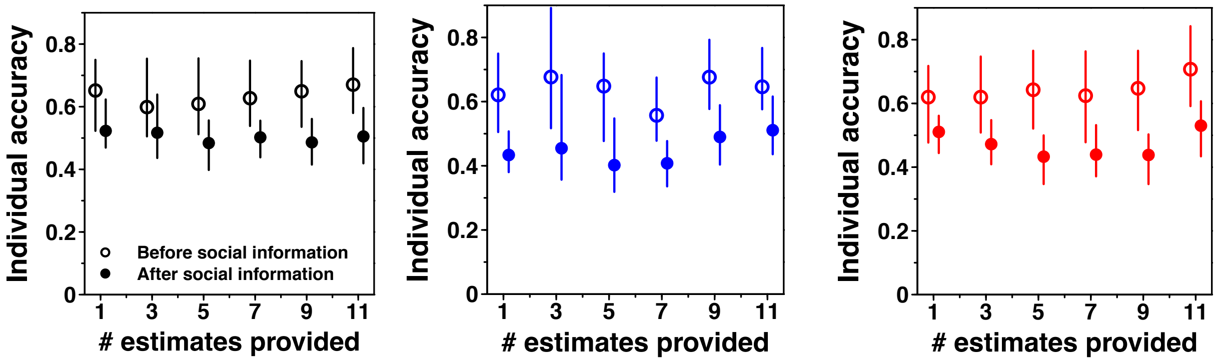

The higher performance of the Shifted-Median treatment is clearly visible in the graphs showing collective accuracy. At the reviewer's request, we now included these graphs in the Supplementary Information and mention them in the main text (lines 178-179).

Below we show the equivalent graphs, but without the log, i.e., the quantities presented are the values: $\text{Median}_{i,q}(E_{i,q} / T_q)$. The benchmark for perfect accuracy is now 1, instead of 0.

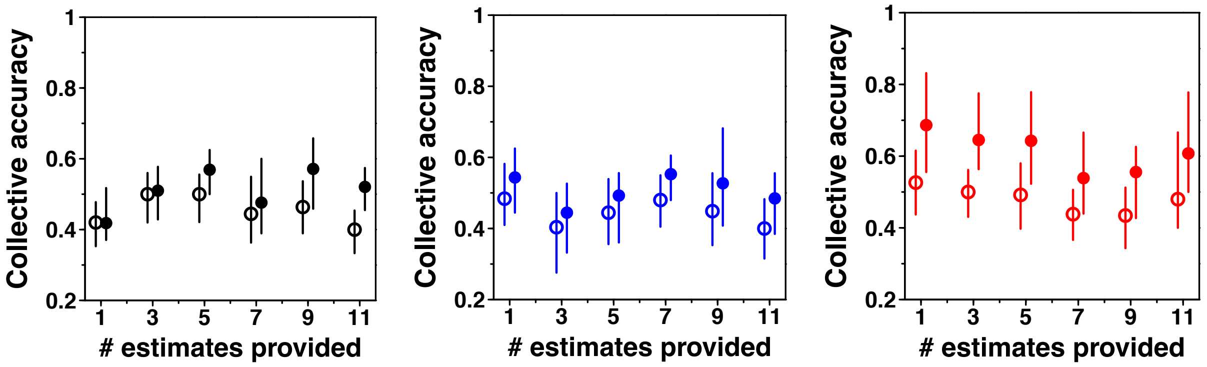

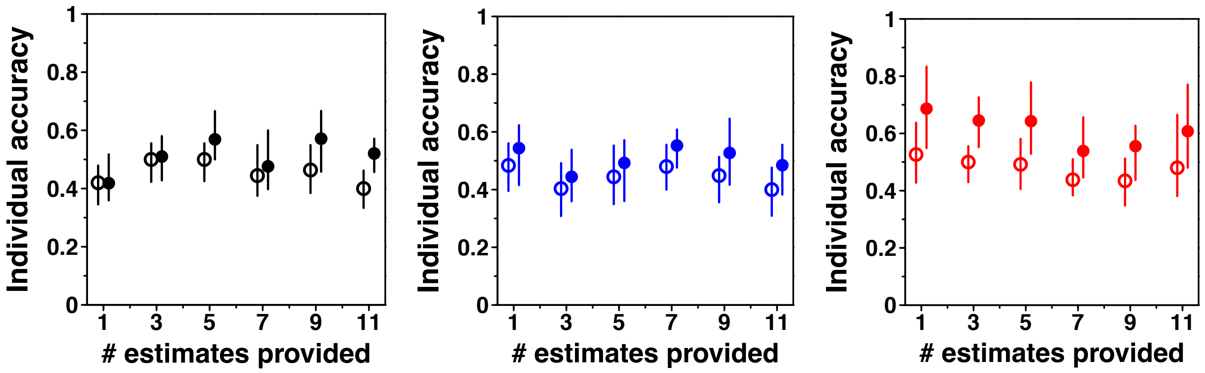

The patterns are highly similar, so we decided to not include these graphs in the manuscript.

3.c. Second, the relative improvement calculation seems to be used in an absolute sense rather than in a relative sense. Intuitively, I would expect this quantify to be calculated based on the difference between the E_P and E_S divided by E_P. The authors are asked to specify how they calculate relative improvement and potentially modify this term to emphasize that it is based on the incremental similarity to T rather than to the change from E_P.

Reviewer #3 also alerted that the paragraph in which we define collective and individual accuracy was not clear enough. We completely revised this section, and believe it is now much clearer. The relative improvement is computed as the difference between collective/individual accuracy before and after social information sharing, divided by collective/individual accuracy before social information sharing. We now state it explicitly at the beginning of the results section (lines 169-171).

4. Relevance of effects. On page 10, the authors claim that the measured effects act independently to improve the collective improvement patterns. How did they determine this? Did they do so by considering each of them separately and their different combinations? Such a design would allow a definitive ranking of the importance of the effects, but I believe the text refers to just one sequence of adding them one after another.

Maybe the sentence starting with “Note that all effects are acting independently, …” in the section describing the model was confusing. This independence of the effects is indeed an assumption of the model, not a claim about the actual effects. We modified the sentence to clarify that this is a model assumption (line 261).

5. Other minor edits. Overall, the paper is well written, but some minor edits should be made to enhance clarity, including:

5.a. Pg. 1, lines 19-20; pg. 2, lines 34-36: The authors claim that prior knowledge on the underestimation bias can be leveraged to select the estimates which, are most likely to counter its effects. As far as I can tell, subjects may not have prior knowledge of the underestimation bias. In the executed experiments, where subjects told about the underestimation bias is? Additionally, did they know that some of the social information received would be shifted upwards to counteract the bias?

Subjects were told no such things, and were only aware of receiving estimates from other group members. Subjects indeed have no prior knowledge about the underestimation bias, otherwise one could reasonably argue that this bias should not exist. The “prior knowledge” in these sentences *refers to the person who selects the estimates*, i.e., the experimenters (or the algorithm built by the experimenters) and *not the subjects*. We now made it clearer throughout the text (see for instance lines 32-34) that this prior knowledge is not leveraged by the participants, but by those who wish to help participants improve their accuracy.

5.b. Pg. 4, lines 95 and 105. Consider changing “estimates which” to “estimates of which”. I believe the latter captures the authors’ intended meaning; the former phrasing is quite confusing/awkward.

Agreed and changed accordingly.

5.c. Pg. 6 lines 147-149. The difference between the formulas for collective accuracy and individual accuracy is not clear. The norm symbol |.| is placed outside the full expression for the former but inside the median function for the latter, but it is hard to see what the exact implication of this is since it is not stated what norm is being used (and what are the ranges of the indices operated over wihin the equations). Please clarify by stating what norm being used or alternatively by defining the explicit sum, division, and other operations involve.

This section was rather unclear in our original submission. As mentioned above, in comment #3.c., the section has been extensively revised in the new version of the manuscript, and is now much clearer. In particular, the norm there refers to the absolute value, which is now explicitly mentioned in the text (lines 161 and 164).

Banerjee, A. V. (1992). A simple model of herd behavior. The quarterly journal of economics, 107(3), 797-817.

John, T. J., & Samuel, R. (2000). Herd immunity and herd effect: new insights and definitions. European journal of epidemiology, 16(7), 601-606.

Moldovan, S. (2010). Investors Psychology And The Herd Effect On The Financial Markets. Revista tinerilor economişti, (15 spec), 21-26.

Simon, H. A. (1954). Bandwagon and underdog effects and the possibility of election predictions. Public Opinion Quarterly, 18(3), 245-253.

****
****

Reviewer #5

In this manuscript, the authors extend previous work, by themselves as well as others in the field, to examine how social influence can affect individual and collective accuracy in estimation tasks. Specifically, here they examine how selecting the how many, and which, estimates to give to participants can improve decision accuracy by counteracting known estimation biases. Among other findings, they find that by providing participants with estimates closer to a modified median can substantially improve collective wisdom. They extend a mechanistic model of social influence incorporating the new phenomena that they identified in this study and find that their model can reproduce, to a large extent, their empirical findings. Furthermore, they use their model to identify an optimal strategy to maximized collective wisdom.

This work is a natural extension of research that has appeared in the past couple of years and makes some important findings by using a clever experimental design that clarifies some outstanding questions related to social influence and the wisdom of crowds. As such, I think that this is an important work that is highly suitable for PLoS Comp Biol. Also, I think that this manuscript is well written and the methods and analyses are sound -- as such, I would recommend publication of this manuscript almost as is. I only have a few minor comments that I hope will improve the clarity of the manuscript:

1. I think the paper Becker et al (2017) Network dynamics of social influence in the wisdom of crowds. PNAS should probably be cited somewhere since it speaks to a lot of the same issues as the present manuscript (how who-influences-who can affect the wisdom of crowds).

This is indeed a very relevant suggestion, and we added it (reference [30]).

2. "our method does not require the a priori knowledge of the truth" (lines 143-144). While I know what the authors mean by this, one could in theory disagree with this statement because the parameterization of their model (i.e., that gamma = 0.9) requires knowledge of some previous truths. However, their method does not require knowledge of the truth for the present estimation task. This could be clarified.

This is absolutely true. The computation of gamma was based on the analysis of previous tasks in the same domain. This is why we talked about “leveraging prior knowledge about the underestimation bias”. The method does not require prior knowledge about the correct answer to the question at hand, but it requires prior knowledge about the task domain to which the question belongs. We now clarified this (lines 111-112).

3. line 192: I would argue that the asymmetry effect was described to some extent in reference 44 of this manuscript: Kao et al (2018) Counteracting estimation bias and social influence to improve the wisdom of crowds. J Royal Society Interface (Disclaimer: since I'm one of the authors of that manuscript, I've signed this review in the pursuit of transparency) In Figure 5a and 5c of that paper, we described a similar effect, where estimates larger than the focal individual's were weighted more heavily than estimates that were smaller. However, the empirical trend in the present manuscript is somewhat different, with a stronger effect size. In any case, it may be useful to point this out somewhere, to show that this asymmetry effect may be robust and widespread (although I refrain from pushing this point too strongly since it is a paper that I'm a co-author on).

After submission, we realized this embarrassing omission. We now added the reference once more in the discussion of the asymmetry effect. This is highly relevant, and indeed suggests that this effect may be robust and widespread (lines 321-323).
