# Supplementary material for: Crowd control: Reducing individual estimation bias by sharing biased social information
Source: PLoS Comput Biol. 2021 Nov 29;17(11):e1009590. doi: 10.1371/journal.pcbi.1009590 (PMC8659305; doi:10.1371/journal.pcbi.1009590)
Supplement: Attachment — Submitted filename: PCOMPBIOL-D-20-00065_R1_Comments_to_Authors.pdf [file pcbi.1009590.s005.pdf]

This report refers to PLOS Computational Biology revised submission PCOMPBIOL-D-20-00065_R1, “Debiasing the crowd: how to select social information for improving collective judgments?”. From my review of both the revised manuscript and the answers document, the authors’ submission has significantly improved. I particularly appreciated the clearer descriptions of the authors’ approach and of the limitations of the results. Having said that, I would like to ask the authors to satisfactorily address two key points that follow from their revision.

- Include a comparison with recalibrated aggregation method suggested by Reviewer #3. While the authors claim in the responses document that their goal is on boosting the accuracy of individuals the paper also highlights prominently the improvements/accuracy of the collective estimates (this is reflected in both the title and in the abstract, for example). In the responses document, the authors already mention that the recalibration method outperforms the shifted median in collective accuracy. The authors should also compare how the recalibration method fares in terms of individual accuracy (i.e., after individual estimates are increased according to γ). Furthermore, based on the aforementioned emphasis on both individual and collective estimates in this paper, I believe this comparison should be explicitly included in the paper—or summarized in the body of the paper but included in the appendix, if it is too lengthy.

- Inclusion of analysis in authors’ response to my “accuracy of estimates” comment (3.a. in the responses document). From the author’s additional figures provided in response to comment 3.a., it seems that benefit of their approach is on collective improvement and accuracy rather than on individual improvement and accuracy (which further motivates my suggestion above). These takeaways were not as clear when all instances were grouped together. Once the instances are separated into instances with ground truths with very large values (call these type-1 instances, for short) and instances with ground truths with moderately large values (call these type-2 instances, for short), this becomes even clearer than with Figure 2. In particular, collective improvement is convincingly superior with the shifted median than with the median for both type-1 and type-2 instances. On the other hand, the median leads to higher individual improvements in type-2 instances in 4 of 6 domain values considered in the plot, while the shifted median leads to higher individual improvements in type-1 instances in 4 of 6 domain values considered in the plot. In fact, more social information seems to diminish the individual improvement of the shifted median for type-1 instances, but it increases it for type-2 instances (I believe the former trend is reported in the paper and responses but not the latter, potentially because the latter trend is lost when combining all instances). Based on these observations, the conclusions about the effect on individual improvement (and similarly for individual accuracy) of the proposed approach warrant further analysis. The authors are asked to analyze and contextualize these results further and to revise their related conclusions, if needed.
