# Supplementary material for: Crowd control: Reducing individual estimation bias by sharing biased social information
Source: PLoS Comput Biol. 2021 Nov 29;17(11):e1009590. doi: 10.1371/journal.pcbi.1009590 (PMC8659305; doi:10.1371/journal.pcbi.1009590)
Supplement: Attachment — Submitted filename: Response to Reviewers.pdf [file pcbi.1009590.s006.pdf]

# Response to the Reviewers

Dear Editor,

We are grateful for the opportunity to revise our manuscript and thank the reviewers for their critical feedback. In the revision, we address all the reviewers' comments. Moreover, and also following several reviewer comments, we decided to restructure the presentation of the results. We now first present the results that will serve to build the model, then describe the model. Finally we use the model to analyze other results that were not used for building the model. Furthermore, we now provide a more thorough statistical analysis. In doing so, we greatly benefitted from the expertise of Dr. Clément Sire. His input was very substantial, therefore, we decided to make him a co-author. We hope this is acceptable, and are happy to provide more detailed information on Dr. Sire's contribution if required.

The storyline and main results have remained the same. However, we would like to point out a few important changes. First, relative improvements in (collective and individual) accuracy have been replaced by collective and individual accuracy before and after social information sharing. Relative improvement is a rather unreliable measure, as it involves a division by a quantity that can be close to 0. Showing individual and collective accuracy before and after social information sharing is more reliable, and also more informative. Second, we removed the predictions of the model at different group sizes and values of gamma (the shifted-median parameter), i.e., the very last figure in the previous version of our manuscript. The paper is already very dense with results, and these predictions were rather speculative. Third, we removed the description of the pilot experiment from the Supplementary Information, and now focus exclusively on the main experiment.

We believe that this revised version has significantly improved the strength and readability of our results, and we hope that you and the reviewers will judge our manuscript acceptable for publication in *PLoS Computational Biology*.

Yours sincerely,

Bertrand Jayles, Clément Sire and Ralf Kurvers

**Reviewer #1: I am grateful to the authors for providing a detailed response to my previous review, and note that they have made substantial revisions to address reviewer comments. I would also note that having read the other reviews, I find that I am somewhat out of step with the other reviewers in my assessment of the interest/importance of the manuscript. I have no technical objections to the work in the manuscript, but below I would like to justify why I continue to believe the results are below the importance threshold for PLoS CB.**

**In their response to reviews the authors have repositioned their study as a test of how individual estimates of unknown quantities can be improved in social contexts. They justifiably note that they did not seek to find out how to best combine independent estimates to obtain collective accuracy. However, the text of the manuscript does not concord with the purported focus on improving individual judgements. The title of the manuscript points to the goal of improving collective judgements as the primary goal, a pattern that is repeated through the abstract (L8-9, L19, L26) and introduction (L55-56, L57-58, L69). In the results, on the occasion that individual and collective improvements are at odds, collective improvement is favoured (albeit at only minor cost to individual improvement, L 272-273). While the innovative experimental treatment (shifted-median) offers clear collective improvements, the benefit at the individual level is far from clear when compared to more standard information exchange (either random or median, Fig 2.). Only in the discussion is the revised goal of individual improvement placed at the fore (L361-362), but this is a departure from the large majority of the material that comes before.**

**As a result of the above, I don't think the study can be evaluated without putting the collective efficacy of the methodology at the forefront; collective improvements are not presented as a happy consequence of individual improvement, but stand out as the most noted and noteworthy results. In this light, I consider that my previous criticism of the manuscript broadly**

**remains: as long as the sharing of carefully chosen social information has to be coordinated by a central agent that knows the full distribution of initial estimates, it does not offer a efficient alternative method for 'improving collective judgements' as offered by the title of the manuscript. Since it is well-established that individuals will adjust their estimates towards those they see from others (on average), it is unsurprising that carefully choosing what they see so as to make it (statistically) more accurate will produce more accurate estimates overall. If this is coordinated centrally it is much the same *as if* individual estimates had been combined in an optimal way. If a mechanism could be designed to facilitate this biased information sharing operate endogenously (thus removing the central organising agent) then this criticism would fall away, but I cannot suggest any way to achieve this, especially as the central coordinator must also decide in advance if this estimation problem is one that belongs to the domain of typical under- or over-estimation.**

We understand Reviewer #1's criticisms, which are at two levels: (i) how the main results are presented (collective VS individual accuracy) and (ii) the main interest of our method (using social interactions versus combining personal estimates).

For the first part, the confusion most likely came from us using several confusing expressions (e.g. "improving the accuracy of individuals"). This may have given the erroneous impression that we are primarily interested in improving individual accuracy. However, we are equally interested in improvements (after social information sharing) in both collective and individual accuracy and designed an experiment to test how the Shifted-Median treatment shapes both. In our results, we indeed find that the Shifted-Median treatment yields substantially higher improvements in collective accuracy compared to the Random and Median treatments, while the gains in individual accuracy are at par with the other two treatments. But also note that gains in individual accuracy are much more substantial when comparing the Shifted-Median treatment to an earlier suggested recalibration method on the personal estimates directly (Suppl. Fig. 12). In the revision, we are more careful in our phrasing of the results. We discuss improvements in both collective and individual accuracy, and explain better that our Shifted-Median treatment successfully counters the underestimation bias, thereby boosting collective accuracy as compared to both other treatments. We removed sentences such as "helping individuals improve their accuracy", to remove the confusion. However, we would like to point out that when improving in collective accuracy, it is – we believe – correct to say that individuals improve in accuracy, even though this is at the collective level.

As for the second part, we agree that a "central-agent-free" mechanism (if ever possible) would be very interesting, but that does not make our proposed mechanism less relevant. Combining personal estimates in an optimal way is useful from an external assessor's point of view (for instance to predict future events in a Wisdom of Crowds-like approach), but does not help improving individual or collective judgments. Social interactions reflect more general life situations, and our method shows that it is possible to share social information among individuals in a group more efficiently than the more classic Random sharing. And in particular, that it is possible to rewire social interactions so as to dampen the effects of cognitive biases. We do not agree that it is uninteresting, and hope to have now better presented our results.

Last, we would like to point out that the interest of our manuscript does not lie solely in the description of a method aimed to improve estimation accuracy. We also shed light on important mechanisms of social information integration, and provide a model that shows the core role of these mechanisms in explaining the empirical observations, which it predicts accurately.
In particular, we provide a detailed account of how people integrate multiple estimates in updating their own, and the strong role played by the dispersion of these estimates. We also respectfully disagree with the sentiment that our results are 'unsurprising'. As our extensive analyses of social information uptake show, the mechanisms at play are quite sophisticated, and the effect of a certain sharing mechanism is not necessarily trivial to anticipate.

**Reviewer #2: I am happy with the responses and revisions of the authors and I think the papers is publishable in the current form.**

---

---

**Reviewer #3: This is an interesting paper reporting a model and an empirical study of collective judgment. The study seeks to understand the effects of information sharing in groups and in particular the effects of the amount of information shared and the selection process of which pieces of information are shared. A particular point of interest from the authors' perspective is how well can a particular process ("shifted median") designed to counter natural individual judgment biases improve the quality of the judgments. I like the topic and the approach. The experiment is well designed and, for the most part, it is well analyzed and clearly reported.**

**The authors addressed seriously most of my reservations and this version is much better. I have few outstanding issues/questions:**

**1. In clarifying the underestimation effect, the authors state "The underestimation bias is a widely documented human tendency to underestimate large quantities (typically larger than 100) in estimation tasks". Their explanation and qualification with regard to the underestimation effect is improved. However, this example of "typically larger than 100" seems a bit odd. Isn't there some degree of scale dependence? Would one expect the bias in describing something as a matter of 120 seconds, but not 2 minutes? The authors proceed to provide domain examples where the underestimation effect could be expected. It strikes me that it might be preferable to expand on one of these examples a bit rather than use a sort of arbitrary, scale-free pseudo-criterion like 100. What it is it about population estimates that makes it susceptible to the bias? Can you perhaps describe, or provide an example of, the distribution and its key properties? It also seems like this would serve as good motivation for the log transformations performed (though this is more clearly explicated in the revised version, which I appreciate).**

We agree that the "100 criterion" is rather arbitrary, and removed it from the text. Instead, we now, as suggested, expand on one example. As example, we present a study using a dot estimation task, which finds evidence for an underestimation bias already when people estimate more than 10 dots in an image. Moreover, we now also present a proposed mechanism of the underestimation bias, namely the internal compression of perceived stimuli (lines 76 - 79).
We, however, kept the motivation for the log transformation separated, as the logarithmic internal representation of numbers may not necessarily be the same phenomenon as the compression.
We may indeed perceive numbers logarithmically, and on top of that, use some non-linear compression that makes us underestimate numbers. We, therefore, kept the explanation for using logarithms at the beginning of the section "Compensating the underestimation bias", where we discuss experimental estimates for the first time (lines 164 - 168).

**2. The definitions of collective and individual accuracy are much improved. One further suggestion is that they could perhaps use slightly more qualitative motivation, more like what was provided in their response letter: "individual accuracy is a measure of the distance of an individual's estimates from the truth, and collective accuracy is a measure of how far the central tendency of the estimates of the group is from the truth." I think this sentence is helpful in what is a critical and potentially confusing distinction, and it is worth including something to this effect in the manuscript.**

We agree and added a sentence describing what collective and individual accuracy represent, in less formal terms (lines 379-380).

**I am puzzled by the prediction regarding the random condition. The idea that people would be insensitive to the number of pieces of advice presented to them is counterintuitive (after all, everyone can do exactly what the authors are doing in the median condition, namely reject/ignore extreme values), and is inconsistent with empirical evidence about the way people aggregate information from multiple sources (e.g., Budescu & Rantilla ,2000; Budescu, Rantilla, Yu & Karelitz, 2003; Budescu & Yu, 2007).**

Looking at our empirical results (new Figure 10), we observe that people are indeed largely insensitive to the number tau of estimates presented to them in the Random treatment. On the contrary, the impact of tau on sensitivity to social influence is very strong in the Median and Shifted-Median treatments. What our results show is that, <u>in the conditions of this experiment</u>, the impact of tau reflects an underlying effect of the dispersion of estimates. Since in the Random treatment, the dispersion is independent of tau (new Figure 5), it follows expectedly that <S> is also independent of tau in this treatment. But our results do not exclude the possibility that different experimental conditions (as e.g. referenced above) do find a possible impact of tau.

**4. Line 296: The tradeoff between bias and other factors in WoC is analyzed in Davis-Stober, Budescu, Dana & Broomell, 2014).**

Thank you for this very relevant reference! We added it at the end of the sentence, line 470.

**5. The authors seek to identify the "optimal" adjustment and refer to how close the shift is to the "true" value. I don't know how seriously to take the notion that there is a "true" value. The target they use is based on the degree of underestimation observed in a study using a particular set of items / questions and I am not sure that it would replicate with different items (imagine asking people to estimate distances to various planets). I think some measure of caution and qualification is needed.**

We agree that the discussion of the last figure of the previous version of our manuscript (i.e., the model predictions at larger group sizes and different values of gamma), and especially claims about optimality, were a bit of a stretch. We decided to remove this part from the manuscript, since we substantially expanded the presentation of the empirical results and model.

**6. The one point I remain somewhat unpersuaded about is the explanation regarding the empirical results in figure 2a. Here is the question and response from the response letter:**

**- Question 16: This is especially puzzling, since this does not seem to reflect the actual differences between these groups, where it appears the random selection method was more linearly related to the number of estimates exchanged; while the median selection method was flatter across the number of estimates exchanged.**

**- Answer: We agree that Fig. 2a gives the visual impression that collective improvement in the random treatment increases more linearly than in the median treatment, mostly because the blue point at tau = 1 is way higher than predicted by the model (and expected by us). Apart from this single point, both treatments increase, as predicted, linearly and it is likely that this single point deviated too much from its expected value due to noise, as often happens with limited samples. Though we tested 216 participants over 36 questions, our sample size per unique treatment combination is still limited. So, we believe we need to treat each single point with caution, but can have much more confidence in the general patterns.**

**While the non-linearity intuition may not have borne out, it still seems like this empirical pattern is more than just a single data point. While $\tau = 1$ may be the most extreme deviation from the model prediction, it looks like the error bars for almost every point in the median condition almost entirely overlap. I'm not sure writing it off as sample size noise in a single treatment condition is fully justified. It is a possible explanation, but beyond the (tested) suggestion about non-linearity, isn't a more straightforward alternative simply that in the median condition most of the benefit of social information comes from that first piece of information? The Wisdom-of-Crowds theory tells us that the median response has the expectation of being the most accurate single piece of social information available. Each**

**subsequent piece of information (by definition not the median) is expected to be less accurate, so while the similarity and herding effects may tell us that more advice may increase the magnitude of the belief update, the actual expected value of the advice cannot improve. There is a necessary tradeoff between the impact and accuracy of extra information in this condition. In other words, there is possible theoretical reason to expect that, in the median condition, increasing τ should have less added benefit.**
**The authors suggestion of caution is warranted. However, the full extent the authors pay to this is to parenthetically note "(though it is unexpectedly high for τ = 1)." I'm not sure this is a sufficient treatment of this result, given how easy it is to come up with intuitive explanations. At the very least, further remarks in the context of replication seem warranted, and it may be possible to test this possible explanation on your data as well with minor modifications to your model.**
**Picky**

Reviewer #3 is right. Actually, we realized that relative improvements were not an adequate measure, as explained at the beginning of this response letter. We now show collective and individual accuracy both before and after social information sharing in Figure 12. This presentation shows that collective accuracy improves mildly in the Random and Median treatments, and roughly at the same rate. Moreover, for tau > 1, improvements in collective and individual accuracy in these treatments do not, or barely, depend on tau.

**• Footnote 2: There is no MLE for the "center" of a distribution; there are MLEs for well-defined statistical parameters (mean, median, mode, etc.)**

The maximum likelihood estimation is a method that allows to estimate the parameters of a probability distribution. For instance, the Laplace distribution is (theoretically) described by its center (often called "location" parameter) and width (often called "scale" parameter). The MLE shows that the best estimator of the location parameter (i.e., the center of the distribution), for an empirical distribution, is the median of the distribution. So, MLE does not estimate, for instance, the median of a Laplace distribution, it rather shows that the median is the most likely estimate of the center of the distribution. We therefore would like to respectfully insist that this sentence/footnote is correct.

**• Line 197: Add ($0 \leq S \leq 1$).**

We actually do not constrain S to remain between 0 and 1. As can be seen in the distributions of S (Figure 3), the probability of $S < 0$ or $S > 1$ is not null. There is no immediate need to impose such constraint in our data or model.

**Thank you for the opportunity to review this thought-provoking paper**

**David Budescu**

**References**


**Budescu. D.V. & Rantilla, A.K. (2000). Confidence in aggregation of expert opinions. Acta Psychologica, 104, 371-398.**

**Budescu, D.V., Rantilla, A.K, Yu, H., & Karelitz. T.M. (2003). The effects of asymmetry among advisors on the aggregation of their opinions. Organizational Behavior and Human Decision Processes, 90, 178 – 194.**

**Budescu, D.V., & Yu, HY. (2007). Aggregation of opinions based on correlated cues and advisors. Journal of Behavioral Decision Making, 20, 153-177.**

**Davis-Stober, C.P., Budescu, D.V., Dana, J., & Broomell, S.B. (2014). When is a crowd wise? Decision, 1, 79-101.**

**Reviewer #4: This report refers to PLOS Computational Biology revised submission PCOMPBIOL-D-20- 00065_R1, "Debiasing the crowd: how to select social information for improving collective judgments?". From my review of both the revised manuscript and the answers document, the authors' submission has significantly improved. I particularly appreciated the clearer descriptions of the authors' approach and of the limitations of the results. Having said that, I would like to ask the authors to satisfactorily address two key points that follow from their revision.**

**- Include a comparison with recalibrated aggregation method suggested by Reviewer #3. While the authors claim in the responses document that their goal is on boosting the accuracy of individuals the paper also highlights prominently the improvements/accuracy of the collective estimates (this is reflected in both the title and in the abstract, for example). In the responses document, the authors already mention that the recalibration method outperforms the shifted median in collective accuracy. The authors should also compare how the recalibration method fares in terms of individual accuracy (i.e., after individual estimates are increased according to □). Furthermore, based on the aforementioned emphasis on both individual and collective estimates in this paper, I believe this comparison should be explicitly included in the paper—or summarized in the body of the paper but included in the appendix, if it is too lengthy.**

We thank the reviewer for this suggestion. We now present a direct comparison of the performance of the Shifted-Median treatment to the recalibration method suggested by Reviewer #3 (Figure S12). This comparison is made for both collective and individual accuracy, and reveals that both methods yield comparable improvements in collective accuracy. However, individual accuracy significantly deteriorates with the recalibration method, while it improves strongly with the Shifted-Median treatment. This is now discussed in lines 499 – 507.

**- Inclusion of analysis in authors' response to my "accuracy of estimates" comment (3.a. in the responses document). From the author's additional figures provided in response to comment 3.a., it seems that benefit of their approach is on collective improvement and accuracy rather than on individual improvement and accuracy (which further motivates my suggestion above). These takeaways were not as clear when all instances were grouped together. Once the instances are separated into instances with ground truths with very large values (call these type-1 instances, for short) and instances with ground truths with moderately large values (call these type-2 instances, for short), this becomes even clearer than with Figure 2. In particular, collective improvement is convincingly superior with the shifted median than with the median for both type-1 and type-2 instances. On the other hand, the median leads to higher individual improvements in type-2 instances in 4 of 6 domain values considered in the plot, while the shifted median leads to higher individual improvements in type-1 instances in 4 of 6 domain values considered in the plot. In fact, more social information seems to diminish the individual improvement of the shifted median for type-1 instances, but it increases it for type-2 instances (I believe the former trend is reported in the paper and responses but not the latter, potentially because the latter trend is lost when combining all instances). Based on these observations, the conclusions about the effect on individual improvement (and similarly for individual accuracy) of the proposed approach warrant further analysis. The authors are asked to analyze and contextualize these results further and to revise their related conclusions, if needed.**

As explained in the introduction of this letter, we replaced the graphs showing relative improvements with graphs showing collective and individual accuracy, before and after social information sharing, in all treatments. We also did this for both type-1 and type-2 instances (new Figures S13 and S14). Expectedly, collective and individual accuracy before social information sharing are higher (i.e., closer to 0) for moderately large values than for very large values. However, collective and individual accuracy improve more for very large values than for moderately large values, and collective accuracy improves most in the Shifted-Median treatment, as expected from Figure 12. Please note that the

patterns in Figures S13 and S14 are noisier than in Figure 12 where all questions are combined. We present these figures in the Supplementary Information, and briefly discuss them in the discussion, lines 512 – 515. We hope this clarified the distinction between individual and collective accuracy.

---

---

**Reviewer #5: I appreciate the authors' efforts to respond to the comments and criticisms of _five_reviewers. I had only minor comments in the last round (reviewer 5), and unsurprisingly the authors have sufficiently addressed them in the current revision. However, I did take the time to read through all of the other reviewers' comments, and the authors' responses to them. I have some comments to the authors' responses to these comments, as well as a couple of new comments to the current version.**

**1. Some of the other reviewers (reviewer 1, comment 2; reviewer 2, comment 3; reviewer 3, comment 8) have mentioned the broader context of interventions that researchers could use to improve collective accuracy. To all of these comments, the authors responded that they seek to improve individual accuracy, rather than collective accuracy, thereby differentiating their paper from others. However, I disagree with this characterization of their paper. Collective accuracy, in addition to individual accuracy, comprises a major part of their results (e.g., Figure 2a, Figure 5). Furthermore, collective accuracy is mentioned multiple times, for example in their abstract and introduction (e.g., "cognitive biases... can impair the quality of collective judgments and decisions", "our restructuring of social interactions... substantially boosted collective accuracy", biases at the individual level can have negative consequences at the collective level").**

Reviewer #1 was similarly confused. We believe the confusion stems from our (confusing) expressions such as “improving the accuracy of individual judgments”. By that, we meant that “people’s” accuracy improved with our method, regardless of whether this improvement was at the individual or collective level. We thus did not seek to improve individual accuracy over collective accuracy, or the other way around. Our aim was (and still is) to investigate how our novel method (i.e. the Shifted-Median treatment) affects both individual and collective accuracy. We find that both actually improve (new Figure 12), though it is correct that the Shifted-Median treatment indeed leads to substantially higher gains in terms of collective accuracy as compared to the Random and Median treatments. But also note that gains in individual accuracy are much more substantial when comparing the Shifted-Median treatment to an earlier suggested recalibration method on the personal estimates directly (Suppl. Fig. S12). We are now more careful with our phrasing, and avoid giving the impression that we care more about one type of accuracy over the other. See also our answer to Reviewer #1.

**Therefore, dismissing these reviewers' comments as irrelevant seems unjustified. I think that the authors could go in two directions:**

**(a) Substantially edit their text and results to really focus on improving individual accuracy, as they claim to solely do.**

In our revision we have resolved this confusion by making it clearer that we are interested in improvements in both individual and collective accuracy after social information exchange.

**(b) Address some of the comments made by the reviewers, specifically, how their methodology compares with the universe of alternative methods to improve collective accuracy.**

We do this by comparing the performance of the Shifted-Median treatment to an earlier suggested recalibration method on the personal estimates (Suppl. Fig. S12).

**To add to this, if the authors are OK with manipulating what social information individuals have access to, then why not just generate fake social information, if the goal is simply to maximize individual/collective accuracy? If the authors now know the rules that individuals on average follow, then they could construct a set of completely fake social information that should push the individual exactly towards the correct answer (on average). Selecting only from the set of real social information seems to be a limitation, especially when group size is small. Moving towards fake social information would provide much more flexibility on the part of researchers. If this is true, then perhaps the "optimising collective and individual improvements" section could be modified with this more general intervention.**
**Or perhaps there is an ethical reason to not completely make up social information? If so, it's not clear to me why carefully selecting what social information an individual sees is so different from just making up the information. Perhaps there could be some discussion about the ethical considerations in these kinds of interventions.**

A recently published paper indeed follows such an approach, investigating the impact of incorrect social information on individual and collective accuracy (see reference [21]). Selectively exchanging social information has, however, different ethical implications than faking information for obvious reasons. For example, in our experiment participants were sitting together in a group and were instructed that they would receive estimates from their group members, which was, indeed, what happened. Were we to use fake information, we would need to have resorted to deception. It seems to us that discussing ethical considerations of this sort is beyond the scope of this paper.
However, we do agree that mentioning and briefly discussing the possibility to use fake information, as we now did, is relevant and interesting (lines 493 – 498).

**2. Are the statements made in lines 180-186 backed up by some kind of statistical or quantitative analysis, or just mad by "visual inspection"? Particularly, the statement that "individual improvement also increases with tau in the Random treatment" seems dubious, as is, to a lesser extent, the statement that "individual improvement is generally higher in the Median and Shifted-median treatments than in the Random treatment."**

We agree with Reviewer #5 that our manuscript originally lacked a thorough statistical approach. Moreover, as explained in the beginning of this letter, relative improvements have been replaced by collective and individual accuracy, shown both before and after social information sharing. Patterns, and in particular improvements, are now clearer.

**The slopes of the linear regression lines in Figure 1 could be printed in the figure panels themselves, which I think would improve the clarity of this figure.**

Agreed and done.
