# Supplementary material for: Crowd control: Reducing individual estimation bias by sharing biased social information
Source: PLoS Comput Biol. 2021 Nov 29;17(11):e1009590. doi: 10.1371/journal.pcbi.1009590 (PMC8659305; doi:10.1371/journal.pcbi.1009590)
Supplement: Attachment — Submitted filename: PCOMPBIOL-D-20-00065_R2_Comments_to_Authors.pdf [file pcbi.1009590.s007.pdf]

This report refers to PLOS Computational Biology revised submission PCOMPBIOL-D-20-00065_R2, “Debiasing the crowd: how to select social information for improving collective judgments?”. From my review of the previous revised version and the original submission, I find that there remain significant concerns regarding claims pertaining to the experimental results. Furthermore, this second revision has introduced additional concerns including questions regarding the novelty of the research with respect to the authors’ recent works. Lastly, I like to comment that this version was burdensome to read based on the new organization and on the way the images were not included in line with text (I suspect the latter was inadvertent).

- To start with the experiments results, it is still not clear that the shifted-median treatment is significantly better than the median treatment. Figure 12 shows that the initial collective and individual estimates (before SI) from the median treatment were of lower quality (and higher variance) than the initial collective estimates from the shifted-median estimate (for example, the former has four solid dots on or above the solid line and the latter has only two). In other words, the sample of participants in the shifted-median treatment performed better individually and collectively than the sample of participants from the median treatment before the respective treatments are applied. This suggests that the improvement in collective estimates is partially explained by differences in the samples and partially by the different treatments. Hence, the observation that the shifted-median treatment is significantly better than the median treatment based on a before-after analysis (or based on relative improvement) may be too strong of a claim. The authors are asked to attenuate these claims and/or to provide conclusive evidence that most of the improvement is due to the treatment instead of the samples.

- Please explain why in page 19, lines 412-415, a comparison is being made between the individual accuracy of the three treatments at different values of τ *before SI*. To my understanding, such differences are due to sampling variability since before SI, the participants in all three treatments have been exposed to the same exact set of questions, without any additional information exchange. The author’s statement at this juncture that “This reversed pattern suggests that the shifted-median values tend, on average, to slightly overestimate the truth” seems to be making a claim about the effectiveness of the shifted-median treatment before SI (i.e., before the different treatments are actually applied).

- On a related note, the authors should be careful with making strong claims when very small samples are involved. In a few of the newly added graphs, the authors further split the 12-participant samples involved in each collective estimate data point into two subsamples of potentially uneven sizes (e.g., those with $D<0$ or $D>0$), meaning the reported statistics of the two observed classes involve 6 or fewer participants; please help clarify this point if I am mistaken about this.

- The second major point is that the novelty of this work should be further motivated. Since the initial submission of this manuscript, the authors have published two works:

- [21] Jayles B, Escobedo R, Cezera S, Blanchet A, Kameda T, Sire C, and Theraulaz G (2020). The impact of incorrect social information on collective wisdom in human groups. Journal of the Royal Society Interface 17(170):20200496
- [48] Jayles B, Sire C, Kurvers HJMR (2020). Impact of sharing full versus averaged social information on social influence and estimation accuracy. https://doi.org/10.31234/osf.io/4n8bh.

Note that these works were not referenced in the original submission, and [21] was mentioned in revision 1; the authors did list the first-author's PhD dissertation, which could have included the content that was eventually used for the two above publications.

I bring up these publications for two main reasons. First, I opine that these articles and their findings are discussed as if they were conceived by unrelated parties, which heightens the aura of external validation (unintentionally or otherwise). For instance, in page 17 line 374, the authors open by saying that "In line with previous works [20, 21, 48] we define, for a given group in a given condition, (i) the collective accuracy as …" (this is only one of various instances throughout the paper); additionally, in response (1b) of Reviewer 5 regarding the suggestion to "generate fake social information", the authors mentioned that, "A recently published paper indeed follows such an approach, investigating the impact of incorrect social information on individual and collective accuracy (see reference [21])." Because two of the authors are part of [21] and all three are part of [48] and the experiments are highly similar (particularly for [48], as described in the next paragraph), it behooves the authors to talk about what these papers (and others led by them cited in the paper) accomplished and how the current paper differs from them. Furthermore, I opine that the authors could further address Reviewer 5's aforementioned question based on their findings from [21].

The second related reason for bringing up the above references is that the experimental design and presentation of results overlaps significantly with [48]. Reference [48] seeks to determine the impact on individual and collective accuracy of three treatments regarding the sharing of social information: receiving τ estimates in ascending order, receiving τ estimates in random order, and receiving the geometric mean of τ estimates. These treatments are different than those form the current paper, but they are not highly dissimilar. In fact, the new organization and presentation of results mirrors closely what is done in [48], including the choice of figures. I would like to clarify that the respective computational results are different in each paper; however, even the level of improvements from the sorted treatment and geometric mean treatment from paper [48] seem to be comparable to the levels of improvements achieved by the median and shifted-median treatments in this paper. As a last related point, it is worth mentioning that 35 of the 36 questions asked of participants in this manuscript are also asked in the 42 questions asked in [48], meaning that a direct comparison of the results is certainly possible. In short, the concern here is that if [48] has been published (or is under review) in another journal, this seems to detract from the novelty of this PCOMPBIOL submission. Both research works address a similar goal of improving collective estimates and consider treatments that could be analyzed conjointly, in my view, to conclusive determine which is the best mechanism for sharing multiple pieces of social information among the 6 different treatments.

On the other hand, if the results of [48] are only part of a dissertation and will not be published in a peer-reviewed journal or conference proceedings, then the novelty of the current submission would not be diminished.

The authors are asked to address the two above points as well as the following minor comments that relate mostly to newly introduced content in this revision:

- Using "Accuracy" vertical axes does not seem very intuitive since higher values means lower accuracy; perhaps "Error" would be a more appropriate name.
- A paragraph is needed before start of Dependence of P_g, m_g, σ_g on τ subsection (page 10) to describe the purposes for each of the statistical analyses. Previous headings were much more intuitive.
- Figure 2 differences do not really show "great improvement"; blue seems at least as good as red -- why not superimpose them on a larger graph? It may be a good idea to superimpose Figure 3 images as well. Blue and red fit curves look nearly identical.
- Is S still defined as $S = \frac{X_S - X_p}{M - X_p}$, as in Revision 1? This was erased in Revision 2 and replaced simply with the statement "where S is defined as the weight subjects assign to M, that we call the sensitivity to social influence."
- The restriction on S to $[-1,2]$ at this stage seems somewhat arbitrary. What was the quality of the results before this restriction? How detrimental were they to the new results?
- What is X_{SI}? It is mentioned in page 11 without definition.
