# Supplementary material for: Crowd control: Reducing individual estimation bias by sharing biased social information
Source: PLoS Comput Biol. 2021 Nov 29;17(11):e1009590. doi: 10.1371/journal.pcbi.1009590 (PMC8659305; doi:10.1371/journal.pcbi.1009590)
Supplement: Attachment — Submitted filename: Response to the Referees.pdf [file pcbi.1009590.s008.pdf]

Response to the Reviewers

Dear Editor,

We are grateful for the opportunity to revise our manuscript and thank the reviewers for their critical feedback. In this revision, we address all the reviewers' comments. In particular, we now quantitatively assess the statistical significance of our main results and the quality of our model's predictions (goodness-of-fit). We also now clearly point out that studies [20, 21, 22 (former 48)] involved at least two of the present authors.

We hope that this revised version, clearer and augmented with robust statistical analyses, will be deemed acceptable for publication in *PLoS Computational Biology*.

Yours sincerely,
Bertrand Jayles, Clément Sire and Ralf Kurvers

Guest Editor

1. The contribution of this paper relative to the Interface article from Jayes et al. (2020) [21] and the preprint from Jayes et al. (2020) [48]. As discussed by Reviewer 4, both of these papers have significant overlap with the methods and aims of this paper. Furthermore, despite the overlap in authorship among the papers, they are referenced in the current manuscript as if they are independent support for the foundational arguments used in this manuscript. Instead, it appears like these papers were work done in parallel (potentially as part of one larger project), and this needs to be made more explicit. See detailed comments by Reviewer 4.

These works were designed and carried out as separate research projects (and in collaboration with other research groups not involved in the present study), with experiments performed separately and distant in time (more than one year between each experiment) and in different countries (France and Germany). The framework is the same (estimation tasks), and the methods similar (analysis of social information use in estimation tasks), but the aim and specificities of each paper is different. In particular, the social information shared and presented to participants in each paper was different and aimed at testing a different set of hypotheses. Moreover, we continuously added new questions (as for example in the current manuscript that uses 18 formerly tested and 18 new questions).

We now start by mentioning in the experimental design that these papers share a similar framework and methods, and that we make regular comparisons between them, in particular in the model section and beyond (lines 167-170).

2. Several reviewers (explicit statements by Reviewers 4 and 5) have pointed out that the empirical arguments made by the authors lack statistical rigor. Some examples:

2.a) The value of gamma has been taken from a linear regression, but there are no data about the (adjusted) R^2 for this regression nor even a p-value indicating that this value of 0.9 is significantly different from 1.0. The value of gamma has been said to be visually similar across three different data sets, but no statistical test was used to justify that these three data sets are likely to have the same value (and that that value is significantly different from 1.0).

We now added, in Figure 1, the standard errors of the slopes as well as the adjusted R^2 values, and performed a statistical analysis to support the statements that slopes (i) are statistically different from 1 and (ii) are not statistically different from one another. Note that we also added the Adjusted R^2 in Figure 6, Figure 7, and Figure S2.

2.b) The experimental design makes use of paired before/after data, but the authors do not

formally use any paired statistical analyses that would incorporate statistical blocking to account for variance that otherwise confounds the comparison across the different treatment groups.

We now added paired statistical analyses to evaluate the significance of the differences between treatments (Figures 4 and 12) and of the improvement in accuracy after social information sharing (Figures 12, 13 and 14). See also our answer to comment 2.d. below.

2.c) The authors claim that the simulation model reproduces the empirical results well, but they do not attempt a formal goodness-of-fit analysis.

We now incorporate a goodness-of-fit analysis (GoF) based on a measure analogous to the reduced Chi-squared, and on relative errors (eq. 4 and 5). The values for all relevant figures are shown in Supplementary Table S2.

2.d) In general, the authors need to consider formal statistical analyses when making inferences about any empirical data -- especially when small numbers of replications are used. Visual arguments (or even arguments focused only on comparing means) are not convincing. Reviewer 5 points out that error bars represent a single standard deviation, which is a significant under estimation of confidence intervals. If we visually approximate confidence intervals by doubling the current error bars, the resulting bars show that different treatment conditions show a large overlap in response. Really showing that there is an effect requires a statistical test in this case. Even there is a significant effect demonstrated, the inferred effect size should be discussed.

We now apply, in Figure 4 and Figures 12 to 14, statistical analyses to quantify, in particular, the significance of improvements in accuracy (see Supplementary Figures S14, S17 and S19) or differences between treatments (see Supplementary Figures S7, S15 and S16). One key point to understand is that we do not compare individual, unrelated values of tau, but *functions* of tau. For instance, while improvements in accuracy may indeed not always be significant at individual levels of tau, it becomes highly significant when considering all values of tau altogether (through the average improvement). Intuitively, while a single value of tau being lower after social information sharing than before can indeed be due to chance, it is unlikely that 6 of them being lower is still due to chance.

In addition, the fact that error bars/confidence intervals overlap for two quantities (for the same treatment and value of tau; like in each panel of Fig. 12) can sometimes be misleading when the two quantities are correlated. Hence, despite a large variability between bootstrap runs for each of them, the distribution of the difference can be very significantly, say, positive (meaning that when one is above its average in a bootstrap run, the other is also above its own average). An illustrative example is two random variables A=g and B=g+1 when g is a Gaussian random variable of variance 1. Despite the fact that the error bars for A and B completely overlap, we have B-A=1, meaning that B>A with perfect statistical confidence.

Our paired statistical analyses of differences of relevant quantities indeed confirm the statistical significance of our main claims.

In addition, some details of the experimental design pose some confusion. As pointed out by Reviewer 4, the authors state at one point that the FIRST choice of an individual seems to be influenced by the number of estimates shown to that individual. However, the experimental design should be such that the first choice by an individual occurs before any other choices are displayed. This may indicate that some rephrasing is needed, or the experimental design needs to be clarified.

The estimates before social information are indeed not affected by the treatment, and any differences between treatments in these estimates are due to sampling variability (Figure 12). Nonetheless, it is still possible to look at certain characteristics of individuals, which can result in observing differences in first estimates. For example, individuals using relatively little social information (below-median S,

Figure 14) provided better first estimates than individuals using larger amounts of social information (above-median S, Figure 14). This is what is shown and explained in Figures 13 and 14 and their related paragraphs. In these figures, we select a subset of the population according to specific criteria, and these subsets may differ in accuracy before social information sharing. Such differences, of course, disappear when considering the whole population (Figure 12). We now added a short paragraph for further clarification (lines 515-519).

3.) As discussed by Reviewer 3, the real value of the model over what is already shown empirically is not clear. As the authors have used the model, it should act as a lens helping to bring clear focus on which of several different hypothetical drivers are likely responsible for differences seen in the empirical data. However, the model does not currently complement the empirical data to provide clarity; it seems to supplement the empirical data and possibly just raise more questions.

We would like to respectfully disagree. As mentioned at the end of the model section: “we also evaluated two simpler models, leaving out either the similarity effect ($\beta'\sigma$ term, see Supplementary Figures S8 to S10) or the asymmetry effect ($D_0 < 0$ and $\beta_- \neq \beta_+$, see Supplementary Figure S11 to S13), to evaluate the importance of both effects in explaining the empirical patterns.”. And as explained in the following sections of our paper, the model without the asymmetry effect is unable to reproduce the patterns of Figure 11 or the improvements in collective accuracy in Figure 12. Likewise, a model without the similarity effect is unable to reproduce the patterns of Figure 10. So, our model does “act as a lens helping to bring clear focus on which of several different hypothetical drivers are likely responsible for differences seen in the empirical data.”

Moreover, the model is of importance for other reasons, as we will argue below.

That said, I can understand how the authors might feel that without a computational model (and in light of the comments I will make in point 4 below), there may not be much reason this article would belong in PLOS Computational Biology. If the model really is the strong point of the paper that is what anchors it to this journal, then its contribution needs to be made more clear. If the model is removed from the paper, then I would recommend the authors lean on the relevance of this study to recommender systems (as they have already done). But, in the end, if the paper really becomes an empirical study of human psychology, it may be a better fit for PLOS ONE instead.

We believe that the model is an essential part of this study. Indeed, the model has the same structure as a model used successfully in three previous papers addressing related issues in estimation task experiments (studies made by partly the same authors [20, 21, 22], which we now clarified). The fact that this model (with only slight modifications; now also including the effect of dispersion) shows a good agreement with the experimental data across these four studies strongly suggests that it is highly *robust* to describe different situations in estimation tasks.

Moreover, we think that a key role of the model (of any model, actually) is to offer an explicit *representation* of the system it describes and to help identify the key ingredients of the system. To illustrate, only by using a modelling approach to analyze our data, were we able to identify the asymmetry effect in social information use. Note as well that it is not trivial that the model, built from Figures 6, 8 and 9, is able to correctly predict Figures 10 to 14 (plus the Supplementary Figures).

Taken together, these arguments lead us to believe that the model is a key component of our work and deserves to be included in the manuscript.

4.) The authors have gone to great lengths within the text of the article to focus on how information from the crowd can be used to reduce bias in the individual. Previous comments from reviewers have focused on how this paper is not about collective intelligence so much as leveraging information from an ensemble of other evaluators to help improve outcomes from the next evaluator. Still, the title of the paper starts with, "Debiasing the crowd," which suggests that the paper is about designing information sharing mechanisms to improve group

outcomes. That is simply not the focus of this paper. The authors might want to consider an analogy to "control variates", a method employed for variance reduction in Monte Carlo methods. In variance reduction, each experimental replication has a multivariate output -- one (X) with a mean that is trying to be inferred, and another with a known mean (Y, Ybar). Control variates use the demonstrated correlation between the two response variables (cov(X,Y), which can be estimated from the data) in a similar way as the "S" variable described by the authors. In particular, the response variable (Y) with the known mean (Ybar) generates a difference from that known mean (Y-Ybar), and that difference can be scaled (with magnitude related to cov(X,Y)) to directly adjust the observed value of the focal variable (X). The authors seem to be asserting that a similar process goes on within the head of an individual when making the second prediction, and their method leverages this to try to reduce the bias in an individual. So, a more accurate title might be something like:

"Crowd Control: How to select social information to improve individual judgment accuracy"

Personally, I prefer titles that state the main results as opposed to posing questions that the reader is promised to find a an answer to within. With that in mind, I might suggest something like:

"Crowd Control: Reducing individual estimation bias by sharing biased subsets of evaluations from others"

That said, I find that one thing diluting the value of this article is that it appears to be two things at once. On one hand, it attempts to be a scientific article making inferences about how humans use social information. On the other hand, it attempts to be a design article suggesting how recommender systems (or other technologically enabled systems) might reduce intrinsic bias in the choices made by their users. I think the article would be improved if the authors would focus on one (and possibly leave the other as a short set of comments in discussion related to broader impacts). My personal recommendation would be to focus on better illuminating the four effects that relate to how humans make use of data from existing raters. Then the design comments could be left for (brief) discussion. If this was the focus, then the title of the paper would not be built around the idea of an action ("Debiasing" or "Control") but instead would be constructed to communicate novel psychological insights (e.g., "Human numerical estimation errors are highly sensitive to...").

We agree with the Guest Editor that the title was a bit misleading. We would like to follow (and slightly amend) this recommendation, and suggest the following title: "Crowd Control: Reducing individual estimation bias by sharing biased social information".

However, we respectfully disagree with the second part of the Guest Editor’s comment: our paper does focus on the mechanisms underlying social information use and their resulting effects on individual and collective accuracy. There is only one sentence in the introduction mentioning recommender systems, and one at the end of the discussion, which is therefore in line with the Guest Editor’s own recommendation: the “design comments” are already “left for (brief) discussion”.

Thank you for your time and efforts on this manuscript. I hope you find these comments to be constructive. Best wishes to you in your efforts to further revise this manuscript, if you choose to do so.

Reviewer #3

This is the third time I am reading this paper that reports a model and an empirical study of collective judgments. The study seeks to understand the effects of information sharing in

groups and in particular the effects of the amount of information shared and the selection process of which pieces of information are shared. A particular point of interest from the authors' perspective is how well can a particular process ("shifted median") designed to counter natural individual judgment biases improve the quality of the judgments. I like the topic and the approach. The experiment is well designed and, for the most part, it is well analyzed and clearly reported. The authors addressed seriously my reservations and this version is better. I still have few outstanding issues/questions:

In the previous draft of the paper, I found the examples the authors used for the magnitude of the overestimation bias somewhat arbitrary (they referenced the number 100 at some point). They took my suggestion and, instead, focused on a single example, using a dot estimation task, but I still find this slightly opaque and would prefer it were expanded a bit. Can the authors provide a brief explanation of the design of the task, with particular emphasis perhaps on the range of dots participants were expected to estimate? Just slightly more context would go a long way here I feel.

We now provide slightly more context about this specific example (line 79-81).

I continue to be puzzled by the prediction regarding the random condition. The notion that people are insensitive to the number of pieces of advice presented to them is counterintuitive (after all, everyone can do exactly what the authors are doing in the median condition, namely reject/ignore extreme values), and is inconsistent with empirical evidence about the way people aggregate information from multiple sources (e.g., Budescu & Rantilla ,2000; Budescu, Rantilla, Yu & Karelitz, 2003; Budescu & Yu, 2007). The authors point out that their results in the random condition confirm this expectation, but this seems to be a bizarre twist of logic. I was not questioning their results, but asking for the justification for the a-priori prediction that runs counter to (at least, some) data. One can't defend / justify a prediction, by simply, saying it was right! Do they imply that the prediction was preceded by the results?

The prediction that – we think – puzzles Reviewer #3 is that collective accuracy would not improve, independent of the number of shared estimates. This is, however, very different from saying that "people are insensitive to the number of pieces of advice presented to them", which we never did. Indeed, we predicted increased improvements in individual accuracy with tau, because subjects would copy each other more. We therefore expected them to be sensitive to tau.

An improvement in collective accuracy implies a shift of the whole distribution of estimates. There was no reason to expect that with randomly shared estimates, hence our prediction. However, we were proven wrong, as the asymmetry effect drove improvements in collective accuracy, although mild. There was no way for us to anticipate the asymmetry effect, such that our prediction was perfectly reasonable. Note that we never "pointed out that our results in the random condition confirm [our] expectation", since we were proven wrong. And there is nothing like the above mentioned "twist of logic", we never "justified a prediction by saying it was right".

That being said, we agree that our original prediction regarding collective accuracy in the Random treatment may have been a bit bold, as it was hard to tell what kind of effect may occur when sharing more than one estimate. Following Reviewer #3's suggestion, we have therefore now reformulated this as follows (including the suggested key references):

"Previous research showed that when individuals in groups receive single, randomly selected estimates, individual accuracy improves because estimates converge, but collective accuracy does not [19, 20]. We hence expected to also find improvements in individual accuracy, but not in collective accuracy, at $\tau = 1$. Furthermore, we expected individual and collective accuracy to increase with the number of shared estimates, as we anticipated subjects to use the social information better with an increasing number of shared estimates [43, 44, 45]".

I think we had this argument in a previous round, but I don't think the claim on lines 114-115 is mathematically correct. If all judgments over (or under) estimate the true value (i.e. all errors have the same sign), it is easy to generate distributions where the expected error (i.e. the error

of a randomly selected judge) is smaller than the error of the median. This needs to be clarified.

We did not intend to imply that this is a mathematical necessity, but rather that this is a common empirical finding; this is the very definition of the Wisdom of Crowds. We now slightly rephrase to make that clearer: "Since median estimates in estimation tasks are **typically (but not always)** closer to the true value than randomly selected estimates (Wisdom of Crowds)…" (lines 116-118).

Line 172: What exactly does "reliable" mean here? Please define and clarify whether this is an empirical or a normative / theoretical argument. BTW Han & Budescu (2019) show the superiority of the median over the mean in a bunch of cases, and cite other papers doing so.

This is indeed a normative/theoretical argument. We modified this paragraph to clarify our point: "Because the distributions of log-estimates are usually close to symmetric, the distance between the centre of these distributions and the truth is often used to measure the quality of collective judgments in such tasks (Wisdom of Crowds) [21]. Although the mean is sometimes used to estimate the centre of the distributions of log-estimates, the median is generally a better estimate of it [51], as most distributions are closer to Laplace distributions than to Gaussian distributions [52]." (lines 176-181). Note that we added Han & Budescu (2019) as new reference [51].

Line 196: Provide some data to make the point: "In X of the 18 distributions considered, the MSE (or some other measure) of the second estimate is smaller that the MSE (or whatever other measure you pick) of the first set of estimates".

Newly added Supplementary Figure S4 shows that the dispersion of estimates after social information sharing in **all 18** conditions is smaller than before sharing. The decrease is highly significant in every treatment. We added the number 18 in line 217.

The model. On line 324 you outline a two-stage process: In a certain fraction of cases judges stick to their estimates (S=0) and in the other cases they draw S from a properly parametrized Normal distribution. As a psychologist, I am bothered by the lack of differentiation between individual and aggregate level theorizing. Figure 3 is based on all judges and all items combined and the spike of S=0 does not differentiate between the two. My question, as a psychologist, is whether the S=0 represents a subset of judges that stubbornly refuse to be influenced by social influence, a subset of items that are so easy that no one looks at the others' estimates, or whether this reflects a uniform / universal tendency of all judges to stick to their estimates in a fraction of cases (e.g., when they feel very sure about them.) It is an easy analysis to run (the % of cases where S=0 for every judge and every item) that would help clarify this point.

That is indeed an interesting distinction. The newly added Supplementary Figure S6 shows the distributions of percentages of cases where S = 0, for every judge (participant) and every item (question), along with the model predictions. We added the following sentences in the main text to describe this figure: "The distribution for participants in Supplementary Figure S6a is broad, but the fair agreement between the model and the experimental data suggests that this variability could mainly result from the probabilistic nature of the distribution of S, and not necessarily from a possible (and likely) variability of the participants' individual probability to keep their personal estimate. On the other hand, Supplementary Figure S6b shows that the variability of the fraction of cases where S = 0 is much lower between questions than between participants, in both experiment and model, although the agreement there is only qualitative." (lines 257-264).

The general discussion: I wish some of the four mechanisms listed would be qualified to reflect the restrictions imposed by the experiment. For example, Schultze, Rakotoarisoa and Schulz-Hardt (2015) show that the distance effect is not always monotonic, and it is possible

to imagine case where people would systematically pay more attention to estimates that are lower than theirs (for example, for very rare and / or very undesirable quantities).

We added the reference suggested, and now better discuss restrictions imposed by the experimental design (lines 553-555 and lines 562-564).

Finally, what was Figure 2, and now Figure 12. The new figure contains more information, but I'm not sure it's more informative. What is persuasive about this figure is essentially the same thing as before: when τ is small, median shifted social information benefits collective accuracy meaningfully more than random social information or the unshifted median. I think this is the most interesting result in the paper and was also clear in the previous version of this figure. I don't see how the reference lines for the model predictions are informative / useful, the solid line. This appears to just be the expected pre-advice accuracy estimate across all questions and conditions. How is the model's prediction, which is based on social information, informative in the absence of social information?

First, note that the main reason for changing former Figure 2 to new Figure 12 is that we realized that relative improvements are a rather unreliable measure because they involve a division by a quantity that can be close to 0.

Next, the model predictions in Figure 12 for accuracy before social information sharing (i.e., the solid lines) are based on the Laplace distributions assumption only, and are therefore independent of the social information. They were also not drawn by calculating the average of the 6 corresponding experimental points or the "average (expected?) pre-advice accuracy estimate across all questions and conditions". Estimates are drawn from Laplace distributions for each individual question, such that all estimates used to draw the lines are simulated by the model, and not experimental estimates. See also our answer to a similar comment made by Reviewer #5.

It is, therefore, not so trivial that such a procedure reproduces the distributions of pre-advice estimates—and thereby pre-advice collective and individual accuracy. Note that this procedure also reproduces the relation between the dispersion of estimates and tau (Figure 5) very well.

Moreover, showing both lines aids in giving a visual representation of the range of improvements in accuracy. Finally, note that the model predictions for the equivalent lines (i.e., before social information sharing) in Figure 13 and 14 are not trivial and reproduce the experimental data well, and are related to social information use (see also our corresponding answer to the Guest Editor and to Reviewer #4).

More critically, how can we make a comparison with the empirical results when the model prediction is independent of the empirical results by condition? The pre-SI empirical results are condition specific and don't correspond particularly well to the reference line, which makes comparison between the post-SI empirical results and model predictions extremely difficult. One way to address this might be to leave the solid line out entirely and condition the post-SI model predictions on the pre-SI empirical results. Without something like this, it is very difficult to interpret how well the model is reproducing the empirical patterns. Perhaps this was a hidden issue in the previous versions of this figure, as I may have been implicitly assuming this was the case. Otherwise, this seems like an apples-to-oranges comparison.

The pre-SI empirical results are not condition specific, since estimates were made before any social influence (variability around the "reference line" is noise inherent to experiments with limited samples). This is true both in the model and in the experiment, such that there is no problem comparing the model with the experimental results (we now also added a goodness-of-fit analysis). This is, therefore, no "apples-to-oranges comparison", as the model and experiment do show the same information.

Overall, the results (e.g. Figure 10 in particular) seem persuasive that the model adequately captures patterns in individual behavior with regard to social information, but Figure 12 stands

out as being difficult to interpret with regard to how well the model is capturing empirical patterns in collective accuracy.

We hope that our new paired statistical analyses and goodness of fit help to better quantify how well the model is capturing empirical patterns in collective accuracy in Figure 12.

Thank you for the opportunity to review this thought-provoking paper

David Budescu

Han, Y. & Budescu, D.V. (2019) A universal method for evaluating the quality of aggregators. Judgment and Decision Making, 14, 395-411.

Schultze, T., Rakotoarisoa, A., Schulz-Hardt, S. (2015) Effects of distance between initial estimates and advice on advice utilization. Judgment and Decision Making,

Reviewer #4

This report refers to PLOS Computational Biology revised submission PCOMPBIOL-D-20-00065_R2, "Debiasing the crowd: how to select social information for improving collective judgments?". From my review of the previous revised version and the original submission, I find that there remain significant concerns regarding claims pertaining to the experimental results. Furthermore, this second revision has introduced additional concerns including questions regarding the novelty of the research with respect to the authors' recent works. Lastly, I like to comment that this version was burdensome to read based on the new organization and on the way the images were not included in line with text (I suspect the latter was inadvertent).

- To start with the experiments results, it is still not clear that the shifted-median treatment is significantly better than the median treatment. Figure 12 shows that the initial collective and individual estimates (before SI) from the median treatment were of lower quality (and higher variance) than the initial collective estimates from the shifted-median estimate (for example, the former has four solid dots on or above the solid line and the latter has only two). In other words, the sample of participants in the shifted-median treatment performed better individually and collectively than the sample of participants from the median treatment before the respective treatments are applied. This suggests that the improvement in collective estimates is partially explained by differences in the samples and partially by the different treatments. Hence, the observation that the shifted-median treatment is significantly better than the median treatment based on a before-after analysis (or based on relative improvement) may be too strong of a claim. The authors are asked to attenuate these claims and/or to provide conclusive evidence that most of the improvement is due to the treatment instead of the samples.

Accuracy before SI is treatment independent. As explained above in response to Reviewer #3's last comment, the variability in first estimates between treatments is due to noise inherent to experiments with limited samples. We now provide paired statistical analyses showing that improvements in collective accuracy are substantially higher in the Shifted-Median treatment than in the Random treatment (new Fig. S15). We also show that differences in improvements in collective accuracy between the Median and Random treatments are, on the contrary, not statistically significant.

- Please explain why in page 19, lines 412-415, a comparison is being made between the individual accuracy of the three treatments at different values of τ *before SI*. To my understanding, such differences are due to sampling variability since before SI, the participants in all three treatments have been exposed to the same exact set of questions, without any additional information exchange. The author's statement at this juncture that "This reversed pattern suggests that the shifted-median values tend, on average, to slightly overestimate the truth" seems to be making a claim about the effectiveness of the shifted-median treatment before SI (i.e., before the different treatments are actually applied).

The estimates before social information sharing are indeed not affected by the treatment, and any differences between treatments are indeed due to sampling variability. Nonetheless, it is still possible to look at certain characteristics of individuals and study if they differed in their first estimates. For example, individuals using relatively little social information (below-median estimates, Figure 14) provided better first estimates than individuals using larger amounts of social information (above-median estimates, Figure 14). This is what is shown and explained in Figures 13 and 14 and their related paragraphs. In these figures, we select a subset of the population according to specific criteria, and these subsets may differ in accuracy before social information sharing. Such differences, of course, disappear when considering the whole population (Figure 12). We now added a short paragraph for further clarification (lines 515-519).

Moreover, the sentence: "This reversed pattern suggests that the shifted-median values tend, on average, to slightly overestimate the truth" does not make a claim about the effectiveness of the shifted-median treatment before SI, but about the position of the shifted-median value (with respect to the truth), which is independent of being before or after SI.

- On a related note, the authors should be careful with making strong claims when very small samples are involved. In a few of the newly added graphs, the authors further split the 12-participant samples involved in each collective estimate data point into two subsamples of potentially uneven sizes (e.g., those with D<0 or D>0), meaning the reported statistics of the two observed classes involve 6 or fewer participants; please help clarify this point if I am mistaken about this.

First, note that in all of our figures, it is not 12 individuals answering, but 216. Each of these individuals experienced every condition (i.e., combination of treatment and tau) twice, such that we have 432 answers per condition. This is not a very small sample. Moreover, the separation into D < 0 and D > 0 is not a participant-based separation, but an estimate-based separation: each participant can sometimes fall in the D < 0 category, and some other times in the D > 0 case. We separate their ***answers*** into two categories, as now explicitly stated in lines 495-497 (we replaced the word "cases" by "answers") and in the caption of Figure 13. So, in Figure 13 (and Figure 14 as well), all data points still include all 216 participants. Only the number of answers per condition differs, and is 216 on average (still not a very small sample).

- The second major point is that the novelty of this work should be further motivated. Since the initial submission of this manuscript, the authors have published two works:

o [21] Jayles B, Escobedo R, Cezera S, Blanchet A, Kameda T, Sire C, and Theraulaz G (2020). The impact of incorrect social information on collective wisdom in human groups. Journal of the Royal Society Interface 17(170):20200496

o [48] Jayles B, Sire C, Kurvers HJMR (2020). Impact of sharing full versus averaged social information on social influence and estimation accuracy. https://doi.org/10.31234/osf.io/4n8bh.

Note that these works were not referenced in the original submission, and [21] was mentioned in revision 1; the authors did list the first-author's PhD dissertation, which could have included the content that was eventually used for the two above publications.

Yes, at the time of first submission to *PCB*, these works were not published in a journal yet. In the original submission, [21] was only part of the first author's PhD thesis, and [48] (which is now [22]) was not even conceived. When [21] was published we cited it, and we also cited [48]/[22] when it was uploaded as a preprint. Note that [48]/[22] has now been published in the Journal of the Royal society Interface, and we now cite it as such. Finally, out of transparency, note that reference [57], which uses estimation tasks and in which two of the present authors were involved, was also recently published in the Proceedings of the Royal Society B. We updated this reference as well. We, however, want to stress that these manuscripts do not jeopardize the novelty of the current work. In none of these works did we study how sharing biased social information (using the underestimation bias) would affect individual and collective accuracy.

I bring up these publications for two main reasons. First, I opine that these articles and their findings are discussed as if they were conceived by unrelated parties, which heightens the aura of external validation (unintentionally or otherwise). For instance, in page 17 line 374, the authors open by saying that "In line with previous works [20, 21, 48] we define, for a given group in a given condition, (i) the collective accuracy as ..." (this is only one of various instances throughout the paper); additionally, in response (1b) of Reviewer 5 regarding the suggestion to "generate fake social information", the authors mentioned that, "A recently published paper indeed follows such an approach, investigating the impact of incorrect social information on individual and collective accuracy (see reference [21])." Because two of the authors are part of [21] and all three are part of [48] and the experiments are highly similar (particularly for [48], as described in the next paragraph), it behooves the authors to talk about what these papers (and others led by them cited in the paper) accomplished and how the current paper differs from them. Furthermore, I opine that the authors could further address Reviewer 5's aforementioned question based on their findings from [21].

It did not occur to us that the way we discussed these studies would "heighten the aura of external validation". This was not at all our intention. If anything, we rather tried to avoid over-emphasizing our involvement in previous studies cited. That was obviously a mistake… We now explicitly state that these studies were carried out by the same authors (lines 167-170). See also our answer to the Guest Editor.

We also further developed our answer to the former comment by Reviewer #5 in relation to fake social information (lines 603-612), as requested.

The second related reason for bringing up the above references is that the experimental design and presentation of results overlaps significantly with [48]. Reference [48] seeks to determine the impact on individual and collective accuracy of three treatments regarding the sharing of social information: receiving τ estimates in ascending order, receiving τ estimates in random order, and receiving the geometric mean of τ estimates. These treatments are different than those form the current paper, but they are not highly dissimilar. In fact, the new organization and presentation of results mirrors closely what is done in [48], including the choice of figures. I would like to clarify that the respective computational results are different in each paper; however, even the level of improvements from the sorted treatment and geometric mean treatment from paper [48] seem to be comparable to the levels of improvements achieved by the median and shifted-median treatments in this paper. As a last related point, it is worth mentioning that 35 of the 36 questions asked of participants in this manuscript are also asked in the 42 questions asked in [48], meaning that a direct comparison of the results is certainly possible. In short, the concern here is that if [48] has been published (or is under review) in another journal, this seems to detract from the novelty of this PCOMPBIOL submission. Both research works address a similar goal of improving collective estimates and consider treatments that could be analyzed conjointly, in my view, to conclusive determine which is the best mechanism for sharing multiple pieces of social information among the 6 different treatments.

On the other hand, if the results of [48] are only part of a dissertation and will not be published in a peer-reviewed journal or conference proceedings, then the novelty of the current submission would not be diminished.

Both studies were designed and carried out as separate research projects, with more than a year in between experiments, testing a very different set of research questions and hypotheses. While it is true that the framework (estimation tasks) and methods (analysis of social information use in estimation tasks) are similar, the aim and specificities of each paper are very different. The data exploited and the research questions in both projects were different, with different storylines, such that we are not convinced that piecing them together as a single paper would have actually been better. Moreover, given that we used different populations and different time periods, comparing these results directly would have been, in our opinion, problematic. Note that Reference [48] (now [22]) was just published in the Journal of the Royal Society Interface.

We acknowledge that the methods are very similar to that used in [48]/[22], but that does not compromise the quality or novelty of our results themselves. Indeed, our paper is not a Methodology article. Reusing methods and general designs that have proven successful in past studies is not something to be condemned, we believe, but is how science progresses. We are firmly convinced that the novelty and interest of our work remains unaffected by the publications of [21] or [48]/[22].

To be transparent, we now further discuss similarities and differences between the current and previous works at several places in the text.

The authors are asked to address the two above points as well as the following minor comments that relate mostly to newly introduced content in this revision:

- Using "Accuracy" vertical axes does not seem very intuitive since higher values means lower accuracy; perhaps "Error" would be a more appropriate name.

The terms "collective accuracy" and "individual accuracy" have been used in the several papers by the same authors to describe the measures used here [20, 21, 48/22]. We think it is clearer to use the same terminology, for consistency and to facilitate comparisons between these studies, especially since Reviewer #4 and the Guest Editor ask us to emphasize the relations between these studies.

- A paragraph is needed before start of Dependence of Pg , mg , σg on τ subsection (page 10) to describe the purposes for each of the statistical analyses. Previous headings were much more intuitive.

We added a sentence to emphasize that after fitting the distributions of S with 3 parameters Pg, mg and sigma_g, we look at the dependence of these parameters on tau in each treatment, which is the natural continuation (line 276-277).

- Figure 2 differences do not really show "great improvement"; blue seems at least as good as red -- why not superimpose them on a larger graph? It may be a good idea to superimpose Figure 3 images as well. Blue and red fit curves look nearly identical.

Perhaps this is a misunderstanding, but Figure 2 does not show improvements. It shows the distributions of estimates before and after social information sharing, and incidentally the narrowing occurring there. Note that we do not talk about improvements at this stage (and we also do not suggest anywhere in our manuscript that our treatments lead to "great improvement"). Also, blue and red lines are not "fit curves", but model predictions.

As for superimposing graphs, we wish to argue against it, as one of the main goal of our manuscript is to compare the different treatments. Showing one aggregate panel per figure obscures both the similarities and differences between the various treatments. Note, however, that an aggregate distribution of all the Xp (which can be aggregated as they are treatment independent, contrary to Xs) is provided in Supplementary Figure S5.

Additionally, as a response to one of Reviewer #3's comments, we added Supplementary Figure S4 showing that the dispersion of estimates decreases after social information sharing in all 18 conditions. The decrease is significant in every treatment.

- Is S still defined as S = (X-Xp)/(M-Xp) , as in Revision 1? This was erased in Revision 2 and replaced simply with the statement "where S is defined as the weight subjects assign to M, that we call the sensitivity to social influence."

We did not merely replace the formula by the statement, but also provided the formula corresponding to the statement: Xs = (1-S) Xp + S M (line 241). We, however, followed Reviewer #4's suggestion, and reintroduced the explicit *equivalent* expression of S (line 243), as we agree that this may be easier to follow for the reader.

- The restriction on S to [−1,2] at this stage seems somewhat arbitrary. What was the quality of the results before this restriction? How detrimental were they to the new results?

This is not completely arbitrary. The distributions of S (Figure 3) show that this is a relevant interval of values for S, as the probability to find an S below -1 or above 2 is close to 0. It is partly arbitrary in the sense that we could indeed have chosen -0.9 or -1.5 instead of -1, and likewise for 2, but the results would have been very similar. The point of the restriction is to remove *extreme* values of S, which would distort the average of S in the following analyses. We explain in lines 247-252 why large values of S are meaningless and can affect measures based on S. This restriction *reveals* meaningful patterns, it does not hide them.

- What is X_{SI}? It is mentioned in page 11 without definition.

The definition was provided in the same line as X_{SI}: "the dispersion σ = ⟨|X_{SI} − M|⟩ **of the estimates X_{SI} received as social information**." The X_{SI} are the estimates received as social information. To clarify this point, we slightly modified the sentence to: "the dispersion of the estimates received as social information σ = ⟨|X_{SI} − M|⟩, where X_{SI} denote the estimates received as social information." (lines 304-306).

Reviewer #5

I appreciate the authors' work to improve their manuscript, and to address the comments of all of the reviewers. This version of the manuscript looks _significantly_ revised, so I have a few new comments to this version that have to do with the poor statistical treatment of the data.

1. "visual inspection confirms" (l.181) This is a very weak way to compare whether or not two sets of data are statistically different from one another. I suggest that the authors use a more rigorous statistical method to do this comparison.

We now added, in Figure 1, the standard errors of the slopes as well as the adjusted R^2 values, and performed a statistical analysis to support the statements that slopes (i) are significantly lower than 1 and (ii) are not significantly different from one another. Note that we also added the Adjusted R^2 in Figure 6, Figure 7, and Figure S2.

2. "we find narrower distributions after social information sharing" (l.196) This is really not obvious to me, especially if you ignore the lines (which are 'model simulations') and just look at the datapoints. Again, here a rigorous statistical method to make this claim is needed.

Newly added Supplementary Figure S4 shows that the dispersion of estimates decreases after social information sharing in all conditions. The decrease is significant in every treatment.

3. "the distributions of Xp (solid lines) are simulated by drawing the Xp from Laplace distributions" (l.198) This is odd. There is a closed-form expression for the PDF of the Laplace distribution, so simulating this distribution is unnecessary. Furthermore, it appears that the authors simulated the distribution once, and plotted the same simulation across all of the panels of figure 2, and all of the stochastic jaggedness is identical across the panels. Why not just plot the exact form of the distribution?

Laplace distributions are defined by 2 parameters: their centre c and width w. There is one Laplace distribution per question, such that the distributions shown in Figure 2 are NOT Laplace distributions (which would be linear on both sides of c), but the combined outcome of 36 different Laplace distributions, with different centres and widths (measured from the data for individual questions). The combination of multiple Laplace distributions with different centres and widths is generally not a Laplace distribution, and it is not so trivial that the model, drawing estimates from these simulated Laplace distributions, reproduces well the experimental distributions of Xp (especially in Supplementary Figure S5) and relatedly collective and individual accuracy before social information sharing. This is in strong support of the Laplace distributions assumption. Note that this procedure also reproduces the relation between the dispersion of estimates and tau (Figure 5) very well.

Also, for clarification, we did not plot the same simulations across the panels in Figure 2. The model simulations *before* social information sharing *have to* be the same (but not after social information sharing), because the estimates before social information sharing are condition independent. That the “stochastic jaggedness is identical across the panels” is therefore consistent and a necessity (the small “jaggedness” itself is due to the binning used to plot these distributions and the finite number of simulation runs – 10000).

4. "A similar pattern of social influence strength is observed at intermediate values of tau (tau = 3, 5, 7, or 9), where Pg and mg are substantially higher in the Median and Shifted-Median treatments than in the Random treatment. For sigma_g, we observe a higher value in the Random treatment than both other treatments at tau = 3 and 5, but not at higher levels of tau." (l.248-251) This appears to be a poor analysis of the data shown in figure 4. First, we can take the figure at face value. Doing so, we can see that Pg does not appear to be "substantially higher" in the Median and Shifted-Median treatments compared to the Random treatment (the blue and black error bars overlap quite a lot).

First, note that our sentence refers to intermediate values of tau (3, 5, 7, 9). At these values, the blue and black error bars overlap only for Pg at tau = 9. There is no overlap at all for mg. That being said, we now apply paired statistical analyses to evaluate the significance of the differences between treatments. The method shows that the differences between the Shifted-Median and the Median treatments on the one hand, and the Random treatment on the other hand, are significant (see Supplementary Figure S7).

Similarly, sigma_g does not look higher for the Random treatment compared to the other treatments at tau = 3 (the black error bar overlaps both the blue and red error bars).

We agree that the differences are less obvious for sigma_g. Our method shows, however, that the difference between the Random and the other treatments is significant (see Supplementary Figure S7), although to a lesser extent. We now clarified this (lines 294-295).

Worse, however, is the fact that the error bars represent ONE standard error. If we double the length of the error bars to approximate a 95% confidence interval, we can see that nearly all of the error bars will overlap with one another for most of the figure, rendering the authors

statements about the data in the text unsubstantiated by the data. The authors need to address this.

As mentioned above, we now carried out proper statistical analyses to quantify the significance of improvements in accuracy (see Supplementary Figures S14, S17 and S19) or differences between treatments (see Supplementary Figures S7, S15 and S16). One key point to understand is that we do not compare individual, unrelated values of tau, but *functions* of tau. For instance, while improvements in accuracy may indeed not always be significant at individual levels of tau, it becomes highly significant when considering all values of tau altogether (through the average improvement). Intuitively, while a single value of tau being lower after social information sharing than before can indeed be due to chance, it is unlikely that 6 of them being lower is still due to chance.

In addition, the fact that error bars/confidence intervals overlap for two quantities (for the same treatment and value of tau; like in each panel of Figure 12) could be sometimes misleading when the two quantities are correlated. Hence, despite a large variability between bootstrap runs for each of them, the distribution of the difference can be very significantly, say, positive (meaning that when one is above its average in a bootstrap run, the other is also above its own average). An illustrative example is two random variables A=g and B=g+1 when g is a Gaussian random variable of variance 1. Despite the fact that the error bars for A and B completely overlap, we have B-A=1, meaning that B>A with perfect statistical confidence.

Our paired statistical analyses of difference of relevant quantities indeed confirm the statistical significance of our main claims.

5. "we find that the center of the cusp relationship is located at D = D0 <0" (l. 292) However, in line 302, we find that "visual inspection was used to fix D0." Again, this is a surprisingly poor method for determining this, and the authors should use a statistically rigorous method instead.

We agree, and now fit D0 locally using an absolute value function (see updates in the Materials and Methods section). Note that the new values are very close to the ones using the previous method. But we fully agree this method is a better approach.
